# Testacosides A–D, glycoglycerolipids produced by *Microbacterium testaceum* isolated from *Tedania brasiliensis*

**DOI:** 10.1007/s00253-023-12870-0

**Published:** 2024-01-12

**Authors:** Jairo I. Quintana-Bulla, Luciane A. C. Tonon, Lamonielli F. Michaliski, Eduardo Hajdu, Antonio G. Ferreira, Roberto G. S. Berlinck

**Affiliations:** 1 Instituto de Química de São Carlos, Universidade de São Paulo, CP 780, CEP 13560-970, São Carlos, SP Brazil; 2 Museu Nacional, Universidade Federal Do Rio de Janeiro, Quinta da Boa Vista, S/N, CEP , Rio de Janeiro, RJ 20940-040 Brazil; 3 Departamento de Química, Universidade Federal de São Carlos, CEP , São Carlos, SP 13565-905 Brazil

**Keywords:** Marine bacteria, Glycoglycerolipids, Molecular networking

## Abstract

**Abstract:**

Marine bacteria living in association with marine sponges have proven to be a reliable source of biologically active secondary metabolites. However, no studies have yet reported natural products from *Microbacterium testaceum* spp. We herein report the isolation of a *M. testaceum* strain from the sponge *Tedania brasiliensis*. Molecular networking analysis of bioactive pre-fractionated extracts from culture media of *M. testaceum* enabled the discovery of testacosides A–D. Analysis of spectroscopic data and chemical derivatizations allowed the identification of testacosides A–D as glycoglycerolipids bearing a 1-[*α*-glucopyranosyl-(1 → 3)-(*α*-mannopyranosyl)]-glycerol moiety connected to 12-methyltetradecanoic acid for testacoside A (**1**), 14-methylpentadecanoic acid for testacoside B (**2**), and 14-methylhexadecanoic acid for testacosides C (**3**) and D (**4**). The absolute configuration of the monosaccharide residues was determined by ^1^H-NMR analysis of the respective diastereomeric thiazolidine derivatives. This is the first report of natural products isolated from cultures of *M. testaceum*.

**Key points:**

• *The first report of metabolites produced by Microbacterium testaceum*.

• *1-[α-Glucopyranosyl-(1 → 3)-(α-mannopyranosyl)]-glycerol lipids isolated and identified*.

• *Microbacterium testaceum strain isolated from the sponge Tedania brasiliensis*.

**Graphical abstract:**

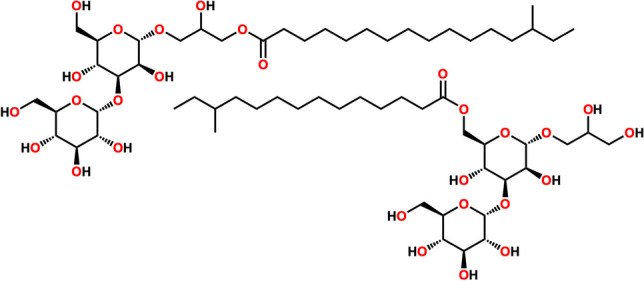

**Supplementary Information:**

The online version contains supplementary material available at 10.1007/s00253-023-12870-0.

## Introduction

Marine sponges (Porifera) are the oldest animals on Earth. Widely distributed all over the world, marine sponges harbor highly diverse and dense microbial communities, which may represent up to 60% of sponge biomass (Webster and Thomas 2016; Hentschel et al. 2012; Van Soest et al. 2012). Although the ecological roles of such microbe-sponge associations are yet poorly known, associated microorganisms are fundamental to maintain sponge health and defense against predators, since it has been shown that many sponge-associated bacteria and cyanobacteria produce various bioactive secondary metabolites (Balskus 2014). In return, sponge-associated microorganisms grow in a nutrient-rich environment (Abdelmohsen et al. 2014; Flórez et al. 2015). Evidences demonstrating the microbial origin of secondary metabolites isolated from marine sponges (Nakashima et al. 2016; Storey et al. 2020; Tianero et al. 2019; Piel et al. 2004; Agarwal et al. 2017) confer to these microorganisms the potential for biotechnological applications towards a sustainable and reliable source of pharmacologically bioactive molecules (Radjasa et al. 2011; Blockley et al. 2017; Brinkmann et al. 2017).

For example, bacteria isolated from marine sponges have been reported to produce antimicrobial agents (Almeida et al. 2023), including in fish aquacultures (Duan et al 2020), as well as several bioactive agents of medical importance (Karthikeyan et al 2022). Also, many metabolites produced by sponge-associated bacteria are of biotechnological interest to the cosmetic industry, such as carotenoids, lipids, melanin, anti-oxidant secondary metabolites, and as biosurfactants (Esposito et al 2021). Therefore, marine sponge-associated bacteria are currently considered of significant biotechnological potential for the production and development of several biotechnological products using green processes, positively impacting bioeconomy (Laport 2017; Romano S, 2018; Brinkmann et al 2017).

Traditional methods for the investigation of bacterial secondary metabolites are labor-intensive and time-consuming, often leading to the re-isolation of known compounds (Berlinck et al. 2019, 2022). Consequentially, an array of new approaches and strategies that enables reliable dereplication of microbial metabolic output have been developed (Gaudêncio and Pereira 2015; Ito and Masubuchi 2014; Wolfender et al. 2019; Beniddir et al. 2021). LC–MS-based metabolomic tools have been applied as effective strategies for the early stage prioritization of microbial bioactive metabolites (Bauermeister et al. 2022; Wang et al. 2016; Quinn et al. 2017).

We have previously investigated the chemistry of the marine sponge *Tedania brasiliensis* and discovered a series of bromopyrrole derivatives (Parra et al. 2018). We then decided to perform an extensive isolation of *T. brasiliensis*-associated cultivable bacteria aiming to obtain bacterial strains to yield media extracts potentially presenting brominated compounds. While the details of this screening searching for brominated metabolites will be reported elsewhere, few bacterial strains were detected to produce bioactive metabolites, among which a strain of the bacterium *Microbacterium testaceum*.

**Fig. 1:**
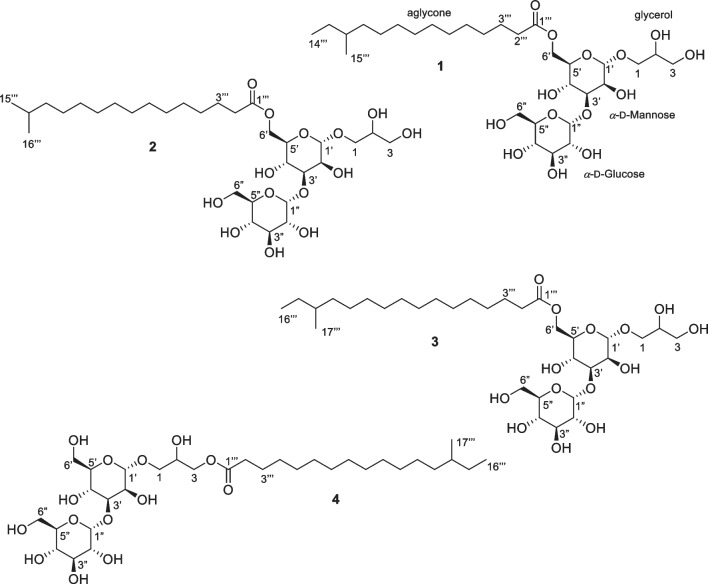
Structures of testacosides **1**–**4**

Thus, the aim of the present report was to investigate the bioactive compounds produced by *M. testaceum* isolated from the sponge *T. brasiliensis*. Analysis of *M. testaceum* media extracts by LC-HRMS/MS indicated a series of related but previously unreported compounds, present in a bioactive fraction. Further scaling up the bacterial culture enabled the MS-guided isolation of four new glycoglycerolipids, named testacosides A–D (**1**–**4**) (Fig. 1). Herein, we report the isolation, identification, and absolute configuration of testacosides A–D, the first metabolites isolated from cultures of a *M. testaceum* strain.

## Materials and methods

### General experimental procedures

Optical rotations were recorded on a Polartronic H Schmidt + Haensch polarimeter. Ultraviolet spectra were recorded on a Shimadzu UV-3600 spectrophotometer. Infrared spectra were recorded on a Shimadzu IRAffinnity-1 Fourier transform infrared spectrophotometer on a silicon plate. NMR spectra were obtained at 25 °C on a Bruker AvanceIII of 14.1 T equipment with a 5-mm cryoprobe, operating at 600.23 MHz for ^1^H and 150.94 MHz for ^13^C, using the residual signal solvent as internal standard. High resolution mass spectrometry (HRMS) data were acquired on a Waters Xevo G2-XS Q-TOF Mass Spectrometer, connected to a Waters Acquity UPLC-H Class system, equipped with a Waters Acquity UPLC BEH C_18_ column (dimensions 2.1 × 100 mm, 1.7 μm). The mobile phase consisted of a gradient of MeCN (Sigma) (+ 0.1% HCO_2_H) in H_2_O (Sigma) (+ 0.1% HCO_2_H), starting from 10% of MeCN to 100% MeCN in 7 min, maintained in 100% MeCN for 2 min, returning to 10% MeCN in 0.1 min, and reconditioning for 0.9 min, with a flow rate of 0.500 mL min^−1^. The column was maintained at 40 °C, and the samples were maintained at 15 °C. Mass spectrometry data acquisition was performed in centroid MS mode, positive resolution mode from 0 to 10 min, with an electrospray ionization (ESI) source and mass range from 100 to 2000 Da, with a scan time of 0.2 s^−1^. The ESI positive mode conditions were 1.2 kV capillary voltage, 30 V cone voltage, 100 °C source temperature, 450 °C desolvation temperature, 50 L h^−1^ cone gas flow, and 750 L h^−1^ desolvation gas flow. A 200 pg mL^−1^ solution of leucine enkephalin (Sigma) was used for internal calibration, infused by the lock-mass probe with a flow rate of 10 μL min^−1^. HPLC–PDA-ELSD-MS analyses were performed on a Waters Alliance 2695 control system coupled online with a Waters 2996 photodiode array detector (PDA), connected sequentially to a Waters 2424 evaporative light scattering detector (ELSD) and a Waters Micromass ZQ2000 low resolution mass spectrometry (MS) detector equipped with a ESI interface. PDA detector was set to detect in the wavelength range from 190 to 600 nm. The ELSD was operated with gain 100, nebulizer heating mode at 60%, drift tube temperature of 80 ± 5 °C, and N_2_ gas pressure of 50 psi. The MS detector was adjusted with the following parameters: capillary voltage 3.00 kV, source temperature 100 °C, desolvation temperature 350 °C, and simultaneous positive and negative ion detection from 100 to 1500 Da. Cone and desolvation gas flow rates are 50 and 350 L h^−1^, respectively. Chromatographic analyses were performed employing a Waters C_18_ X-Terra column (dimensions 250 × 4.6 mm; 5.0 μm), using a mobile phase with a flow rate of 1 mL min^−1^ consisting of (A) Milli-Q grade H_2_O (+ 0.1% HCO_2_H) and (B) 1:1 MeOH/MeCN (+ 0.1 HCO_2_H) as follows: 0–1.0 min hold at 10% B, then a linear gradient to 100% B from 1.0 to 18.0 min, hold at 100% B from 18 to 22 min, followed to 10% B at 22–23 min, and finally column equilibration from 23 to 30 min at 10% B. Volume injection of 20 μL of 2.0 mg mL^−1^ sample solutions was employed. Data acquisition and processing were performed employing the Empower 2.0 software.

### Bacterial strain isolation and identification

The bacterial strain *M. testaceum* was isolated from specimens of the marine sponge *T. brasiliensis* collected between 9 and 38-m depth by SCUBA diving at Arraial do Cabo, state of Rio de Janeiro in January 2017. The strain *M. testaceum* was identified by 16S gene sequencing. The 16S rRNA gene sequencing analyses were performed by the University of Illinois at Chicago Research Resources Center, Sequencing Core, Chicago, USA. The partial 16S rRNA gene sequence (1063 nucleotides [nt]) was submitted to the Basic Local Alignment Search Tool (https://blast.ncbi.nlm.nih.gov) and EzTaxon server (http://eztaxon-e.ezbiocloud.net/; Kim et al. 2012), aligned with 16S rRNA gene sequences of the most closely related *Microbacterium* species using CLUSTAL W and phylogenetic trees were constructed by using MEGA 7.0 (Kumar et al. 2016). Phylogenetic trees were generated using the maximum-parsimony, minimum-evolution (Rzhetsky and Nei 1992), and neighbor-joining (Saitou and Nei 1987) algorithms drawn from the MEGA 7; an evolutionary distance matrix for the neighbor-joining analysis was prepared using the p-distance method (Nei and Kumar 2000) and is in the units of the number of base differences per site. The robustness of the inferred tree topologies was evaluated after 1000 bootstrap replicates (Felsenstein 1985). The analysis involved 12 nucleotide sequences. All positions with less than 95% site coverage were eliminated. That is, fewer than 5% alignment gaps, missing data, and ambiguous bases were allowed at any position. There were a total of 1029 positions in the final dataset. Evolutionary analyses were conducted in MEGA7. The percentage of trees in which the associated taxa clustered together is shown next to the branches. Gene sequences of the identified bacteria were obtained from BLASTN search in the GenBank database with the highest sequence similarities against type strains. GenBank accession number is OQ134920. A reference sample of the strain identified as *M. testaceum* has been deposited in the Brazilian Collection of Environmental and Industrial Microorganisms (CBMAI) under the accession number CBMAI 2824.

### Extraction and isolation

*M. testaceum* strain was reactivated from frozen stocks in marine agar plates (Difco 2216) incubated at 28 °C for 2 days. A seed culture was inoculated by pouring individual colonies into 250 mL Schott flasks containing 100 mL of YEME medium (4 g yeast, 4 g glucose, and 10 g malt extract per liter of artificial sea water ASW) at 28 °C in a rotary shaker at 180 rpm for 3 days. Artificial sea water was prepared as follows: 30.0 g NaCl, 9.68 g MgCl_2_⋅6H_2_O, 3.47 g Na_2_SO_4_, 1.36 g CaCl_2_⋅2H_2_O, 0.610 KCl, 0.170 g NaHCO_3_, 0.1 g KBr, 0.030 g H_3_BO_3_, 0.040 g SrCl_2_⋅6H_2_O, and 0.140 g Na_2_HPO_4_ in 1 L of deionized water. Schott flasks (500 mL, 300 × 200 mL) containing YEME medium and 10 g of Diaion® HP-20 resin were inoculated with 2 mL aliquots of the seed culture and shaken at 28 °C and 180 rpm for 7 days. The resin was separated by vacuum filtration through cheesecloth, washed with deionized water, and extracted with MeOH (4 × 1 L) and acetone (4 × 1 L), then concentrated under reduced pressure to yield 14.3 g of a dry extract. Separately, the culture broth was extracted with an equal volume of EtOAc. Then, the EtOAc fraction was concentrated in vacuum to yield 4.6 g of an organic extract. Both HP-20 resin and EtOAc extracts showed similar chemical profiles by HPLC-MS and were pooled together. The pooled extract was fractionated by C_18_ reversed-phase column chromatography using a step gradient of H_2_O/MeOH from 20% MeOH to 100% MeOH to give five fractions. Fraction 5 (1.0 g) was further fractionated by chromatography on a Sephadex LH-20 column eluted with MeOH, yielding fractions 5A to 5H. Fraction 5C (185.1 mg) was purified by reversed-phase HPLC chromatography using a semipreparative C_18_ InertSustain column (250 × 10 mm, 5 μm) eluted in isocratic mode with H_2_O/MeCN 47:53 (0.1% formic acid) at flow rate of 4 mL min^−1^ and ELS detection, to give fraction 5C2 (testacoside A, 3.2 mg), fraction 5C3 (testacoside B, 2.5 mg), fraction 5C7 (testacoside C, 5.4 mg), and fraction 5C8 (testacoside D, 2.2 mg). Fraction 5D (668.9 mg) was purified by reversed-phase HPLC using a semipreparative C_18_ InertSustain column (250 × 10 mm, 5 μm) eluted in isocratic mode with H_2_O/MeCN 18:82 (0.1% formic acid) at flow rate of 4 mL min^−1^ and ELS detection, to give fraction 5D2 (testacoside A, 16.8 mg), fraction 5D3 (testacoside B, 0.8 mg), and 5D6 (testacoside C, 12.6 mg). NMR data of compounds **1**-**4** are provided in Tables 1 and 2 and NMR spectra in the supplementary information Figures S1-S30.

Testacoside A (**1**): clear colorless glass (0.0200 g); [*α*]^25^_D_ 62.0 (c 0.002, CHCl_3_); IR (neat) *v*_max_ 3360, 1676 cm^−1^; ^1^H and ^13^C NMR data in Table 1; HRESIMS *m*/*z* 641.3733 [M + H]^+^ (calcd for C_30_H_57_O_14_, 641.3748).

Testacoside B (**2**): clear colorless glass (0.0033 g); [*α*]^25^_D_ 72.9 (c 0.004, CHCl_3_); IR (neat) *v*_max_ 3344, 1678 cm^−1^; ^1^H and ^13^C NMR data in Table 1; HRESIMS *m*/*z* 655.3882 [M + H]^+^ (calcd for C_31_H_59_O_14_, 655.3905).

Testacoside C (**3**): clear colorless glass (0.0180 g); [*α*]^25^_D_ + 72.4 (c 0.004, CHCl_3_); IR (neat) *v*_max_ 3369, 1678 cm^−1^; ^1^H and ^13^C NMR data in Table 1; HRESIMS *m*/*z* 669.4041 [M + H]^+^ (calcd for C_32_H_61_O_14_, 669.4061).

Testacoside D (**4**): clear colorless glass (0.0022 g); [*α*]^25^_D_ 62.6 (c 0.002, CHCl_3_); IR (neat) *v*_max_ 3381, 1678 cm^−1^; ^1^H and ^13^C NMR data in Table 2; HRESIMS m/z 691.3864 [M + Na]^+^ (calcd for C_32_H_60_NaO_14_, 691.3881).

### Acetylation of testacosides A–D (1–4)

Approximately 0.5 mg of each compound was dissolved in 1.5 mL of freshly distilled pyridine and 1.5 mL of freshly distilled acetic anhydride at room temperature for 18 h. The reaction mixture was evaporated under a N_2_ stream and the residue was partitioned between H_2_O and EtOAc. The organic phase was washed with H_2_O three times, dried under reduced pressure to give a yellow solid, for further analyses by NMR and HRMS. Data are provided in Tables S1-S2 and spectra in the supplementary information Figures S31-S56.

Testacoside A peracetate (**5**): clear yellow glass (0.0014 g); [*α*]^25^_D_ 43.7 (c 0.001, CHCl_3_); IR (neat) *v*_max_ 1747 cm^−1^; ^1^H and ^13^C NMR data in Table S1; HRESIMS *m*/*z* 999.4453 [M + Na]^+^ (calcd for C_46_H_72_NaO_22_, 999.4407).

Testacoside B peracetate (**6**): clear yellow glass (0.0010 g); [*α*]^25^_D_ 36.9 (c 0.001, CHCl_3_); IR (neat) *v*_max_ 1749 cm^−1^; ^1^H and ^13^C NMR data in Table S1; HRESIMS *m*/*z* 1013.4573 [M + Na]^+^ (calcd for C_47_H_74_NaO_22_, 1013.4564).

Testacoside C peracetate (**7**): clear yellow glass (0.0017 g); [α]^25^_D_ 45.7 (c 0.002, CHCl_3_); IR (neat) *v*_max_ 1747 cm^−1^; ^1^H and ^13^C NMR data in Table S1; HRESIMS *m*/*z* 1027.4727 [M + Na]^+^ (calcd for C_48_H_76_NaO_22_, 1027.4720).

Testacoside D peracetate (**8**): clear yellow glass (0.0010 g); [*α*]^25^_D_ + 38.0 (c 0.001, CHCl_3_); IR (neat) *v*_max_ 1749 cm^−1^; ^1^H and ^13^C NMR data in Table S2; HRESIMS *m*/*z* 1027.4731 [M + Na]^+^ (calcd for C_48_H_76_NaO_22_, 1027.4720).

### Determination of the absolute configuration of sugar residues of 2 and 4

Separately, thiazolidine derivatives of D-glucose and D-mannose standards were prepared by weighting about 2.0 mg of each D-monosaccharide and 4.0 mg of L-cysteine methyl ester hydrochloride, dissolved in 120 μL of pyridine-*d*_5_ in NMR tubes. Reaction mixtures were heated at 60 °C for 1 h and then left at room temperature overnight before ^1^H NMR analysis. Additionally, a reaction with a 1:1 mixture of D-glucose and D-mannose was carried out under the abovementioned conditions. Testacosides B (**2**) and D (**4**) (1.0 mg each) were dissolved in 2 M trifluoroacetic acid (0.5 mL) and stirred at 100 °C for 8 h. After the completion of hydrolysis, the TFA was evaporated in vacuo to obtain the hydrolysate, redissolved in deionized H_2_O (3 mL), and partitioned with CHCl_3_ (3 mL, × 3). The aqueous phase containing the sugar residues was dissolved in 120 μL of pyridine-*d*_5_ and mixed with 1.0 mg of L-cysteine methyl ester hydrochloride to prepare the thiazolidine derivatives from the isolated compounds, as described above. Comparison of ^1^H NMR shifts and *J* coupling constants with standard derivatives was performed to establish the absolute configuration of glucose and mannose as the D-enantiomers.

### GNPS molecular networking analysis

Tandem MS data were acquired on a data-dependent acquisition (DDA) mode with a ramp collision energy (CE) of low CE from 6 to 9 eV and high CE from 60 to 80 eV, for fragmentation of the three most abundant ions. The raw data were converted to mzXML data format using MSConvert (ProteoWizard) (Chambers et. al. 2012; Holman et al. 2014). A molecular network was created following the Classical Molecular Networking protocol on the GNPS platform (Aron et al. 2020). Consensus spectra were generated using the MS-Cluster algorithm with both precursor mass and fragment ion tolerance of 0.02 Da. The network was created with a minimum cosine score of 0.7, a Network TopK of 10, minimum 4 matched fragment ions, and minimum cluster size of 4. Library spectra search options for input data were applied with a score threshold of 0.7 and a minimum of 4 shared fragment ions to be considered as a match. Cytoscape 3.7.0 was used to visualize the network (Shannon et al. 2003). GNPS library spectra search was applied with the same parameters as the input data.

### Antiplasmodial *in vitro* assay against *Plasmodium facilparum* parasites

*Plasmodium falciparum* 3D7 strain parasites (chloroquine sensitive) were cultured as previously described (Trager and Jensen 1976). Freshly sorbitol synchronized ring stages of the parasites (Lambros and Vanderberg 1979) were incubated with the samples at 50 μg mL^−1^ for non-purified fractions and a twofold serial dilution from 10 μM to 0.156 μM for pure compounds, previously solubilized in 0.05% DMSO. Sodium artesunate was used as antiplasmodial positive control. Each assay was performed in triplicate. The activity was measured using the SYBR green assay (Smilkstein et al. 2004). Briefly, the plates were centrifuged at 700 g for 5 min at room temperature to remove the culture medium, washed once with PBS, and incubated for 30 min with lysis buffer solution (20 mM Tris base, 5 mM EDTA, 0.0008% v/v Triton X-100, 0.008% w/v saponin, pH 7.5) and SYBR green DNA stain 0.002% v/v. Plates were incubated at room temperature for 30 min. The fluorescence of uninfected erythrocytes was considered as background. Fluorescence was measured on a SpectraMAX Gemini EM plate reader fluorimeter (485-nm excitation, 535-nm emission). Antiplasmodial activity is calculated relative to the parasite growth control (no compound added, 100% viability) and positive control (0% viability). Results are reported as percent inhibition for fractions and percent viability for compounds.

### Cytotoxicity assay

Cancer cell line MCF-7 (breast adenocarcinoma) and normal breast epithelial cell line MCF-10A were seeded in complete medium containing DMEM/HAM’s F10 medium (1:1, v/v), supplemented with 10% fetal bovine serum and the antibiotic mixture penicillin/streptomycin (10 mL L^−1^) plus kanamycin sulfate (10 mg mL^−1^) in 96-well plates (5000 cells well^−1^) at 37 °C under 5% CO_2_ for 24 h to allow cell attachment. Fractions were added to the cell cultures at concentrations ranging from 0.25 to 250 μg mL^−1^. After 24 h of incubation, the cell plate was washed with PBS; then, a DMEM without phenol red plus XTT/electron solution was added to each well and incubated for 4 h, after which the absorbance was measured at 492 nm and 690 nm. Cell viability was directly proportional to absorbance based on the cleavage tetrazolium XTT by metabolically active cells forming an orange formazan dye, and was compared with the negative control. The inhibitory concentration (IC_50_) was calculated by a nonlinear regression analysis using GraphPad Prism (3.0).

### Anti-leishmania *in vitro* assays

Anti-leishmania activity was evaluated in *Leishmania* (*L*.) *infantum* and *Leishmania* (*V*.) *braziliensis* promastigotes, as previously described (Parra et al. 2018). The anti parasitic activity was assessed using an MTT viability assay, after 24-h incubation with the fractions at concentrations of 50 μg mL^−1^ and 100 μg mL^−1^, and inhibition percentage values were calculated.

### Antibacterial assay

The antibacterial activity was tested against *Escherichia coli* 25,922, *Escherichia coli* 35,218, *Staphylococcus aureus* 33,591, *S. aureus* 25,923, 700,603, *Klebsiella pneumoniae* id-146/19, *Acinetobacter baumannii* 19,606, *Acinetobacter baumannii* 261/16, *Pseudomonas aeruginosa* 27,853, *Pseudomonas aeruginosa* S.6065/06, *Enterococcus faecalis* 51,299, *Enterococcus faecalis* 29,212, and *Enterobacter cloacae* 003/21 (NDM +), and determined by the serial microdilution method in 96-well plates, accordingly with the Clinical and Laboratory Standards Institute protocols (CLSI 2018). Briefly, bacterial strains were initially cultivated in triptone soy agar (TSA) plates at 35 °C for 24 h. Subsequently, a standardized microbial suspension adjusted to 0.5 McFarland (1.5 × 10^8^ CFU mL^−1^) scale was prepared in sterile saline solution and diluted 1:150 in cation-adjusted Mueller Hinton broth. Stock solutions of the compounds were prepared in DMSO at an initial concentration of 100 μM, from which twofold serial dilutions were prepared diluting with cation-adjusted Mueller Hinton broth in the range of 100 to 0.8 μM. Fifty microliters of the bacterial suspension was added to each well of the 96-well microtiter plate. The culture plates were incubated at 35 °C for 20 h. Optical density measurements were made at 630 nm with a microplate reader to obtain growth inhibition values. Vancomycin and polymyxin were used as positive controls. Cell viability was determined by MTT assay.

## Results

### Extraction and isolation of compounds 1–4

*M. testaceum* media were first adsorbed onto HP-20 and then extracted with EtOAc. Both extracts were analyzed by HPLC–MS and showed similar chemical profiles. The extracts were pooled and fractionated by C_18_ reversed-phase column chromatography to give five fractions. The fractions were screened for in vitro antiplasmodial activity against *P. falciparum*, anti-leishmanial activity against *Leishmania infantum* and *L. braziliensis* promastigotes, as well as cytotoxic activity against human cancer cell lines MCF-7 (breast cancer) and the non-cancerous breast cell line MCF-10A. The fraction eluted with 100% MeOH from the C_18_ chromatography column was active against *P. falciparum* with a 99% inhibition at 50 μg mL^−1^, moderately active at 100 μg mL^−1^ against *L. infantum* and *L. braziliensis* promastigotes with cell viability values of 44.6% and 29.6%, respectively, and displayed moderate cytotoxic activity against MCF-7 and MCF-10A cell lines, with IC_50_ values of 32.9 ± 2.2 μg mL^−1^ and 55.4 ± 2.8 μg mL^−1^, respectively.

**Fig. 2:**
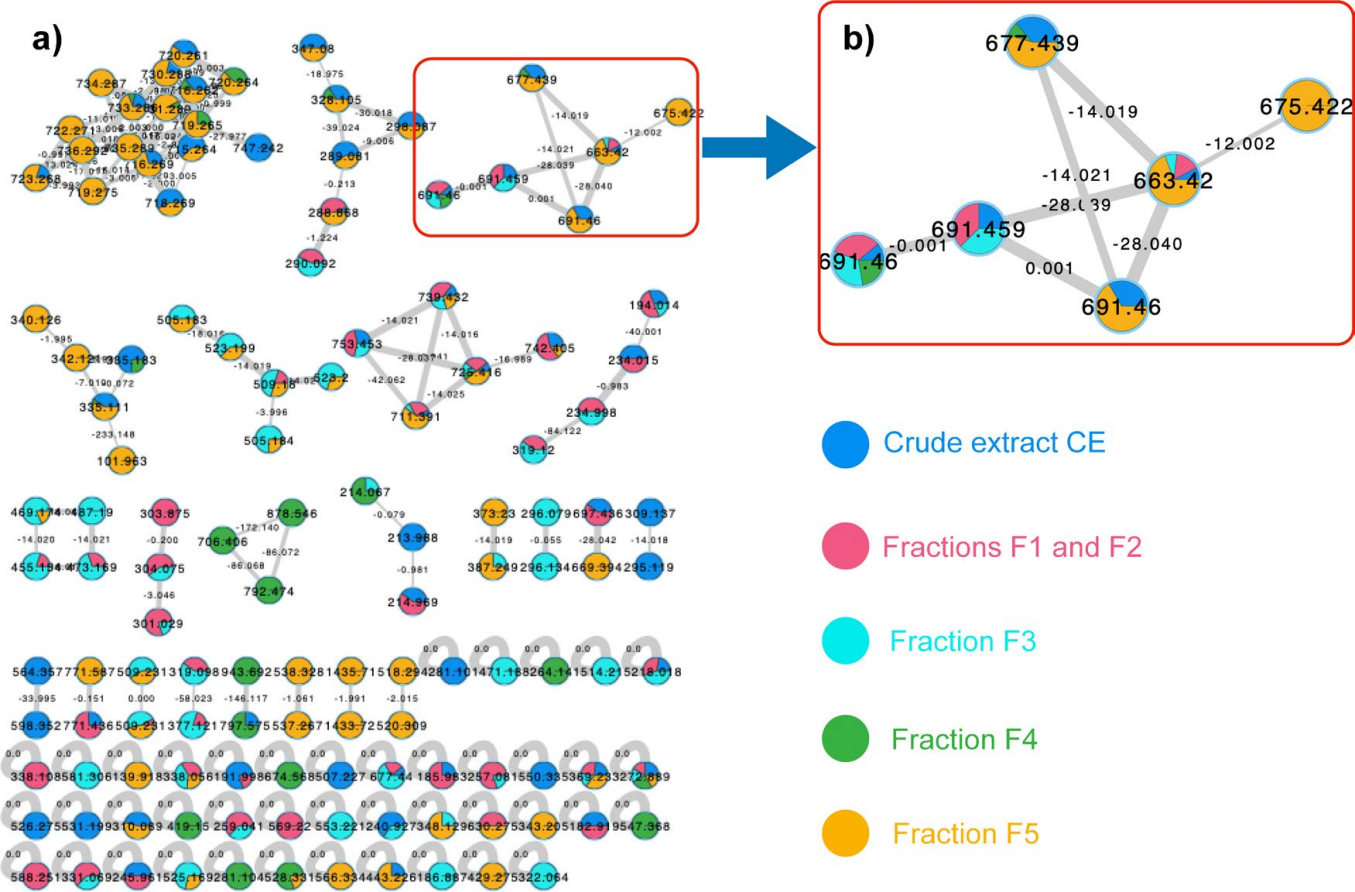
Molecular networking analysis of *Microbacterium testaceum* media extract and C_18_ column fractions. **a** Molecular network of all nodes present only in media extract and C_18_ fractions. Pie charts represent the distributions of the compounds in the fractions. **b** Cluster corresponding to the compounds present in the most active C_18_ fraction F5 (eluted with 100% MeOH)

The C_18_ column chromatography fractions were subjected to molecular networking (MN) analysis (Fig. 2a) (Aron et al. 2020). Our attention was directed to a cluster of ions with *m/z* 663.42, 677.44, and 691.46, present in the most active fraction (F5, Fig. 2b). Mass differences of 14 and 28 Da between the MN nodes indicated that these were homologous compounds. Automated comparison of ions *m/z* 663.42, 677.44, and 691.46 with spectral libraries at the GNPS platform did not annotate any known compound with our experimental data. Manual dereplication was then performed with the Dictionary of Natural Products database, resulting in no matches. These results directed the efforts towards the isolation of the compounds highlighted in the network, guided by MS analyses and leading to the isolation of testacosides A–D (**1**–**4**).

### Structural elucidation of testacosides A–D (1–4)

Testacoside A (**1**) was obtained as a clear colorless glass, with a molecular formula of C_30_H_56_O_14_ deduced by HRESIMS, which showed a [M + H]^+^ ion at *m/z* 641.3733 and a [M + Na]^+^ ion at *m/z* 663.3556 (Fig. S1), indicating 3 degrees of unsaturation. The ^1^H NMR spectrum of testacoside A (**1**) (Table 1, Fig. S2) revealed a group of resonances between *δ*_H_ 3.2 and 5.1, typical of oxymethine protons, and a series of aliphatic methylene and methyl proton resonances between *δ*_H_ 0.9 and 2.4, suggesting an aliphatic hydrocarbon fragment. Analysis of ^13^C NMR, DEPT-135 and HSQC spectra (Figs. S3-S5) indicated two sugar moieties based on typical anomeric signals at *δ*_C_ 102.4 (*δ*_H_ 4.74) and *δ*_C_ 102.2 (*δ*_H_ 5.13), in addition to thirteen oxygenated methine and methylene carbon resonances between *δ*_C_ 62.9 and 82.0. A signal at *δ*_C_ 175.7 was assigned to a carbonyl carbon, completing the unsaturation number calculated by the molecular formula. These NMR features indicated that testacoside A was a glycolipid.

**Fig. 3:**
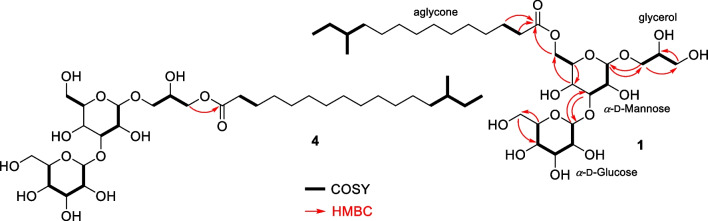
Key ^1^H-.^1^H COSY and HMBC correlations of testacosides A (**1**) and D (**4**)

Analysis of HSQC, COSY, and HMBC (Figs. S5-S7) spectra allowed the assignment of the planar structure of the sugar and aliphatic units (Fig. 3). Two spin systems were identified for the sugar residue 1, from H-1′ (*δ*_H_ 4.74) to H-3′ (*δ*_H_ 3.84), the second from H-5′ (*δ*_H_ 3.78) to H_2_-6′ (*δ*_H_ 4.40/4.23) and connected through C-4′ (*δ*_H_ 3.86, *δ*_C_ 67.7) by HMBC correlations ^3^*J*_CH_ H-4′/C-6′ and H_2_-6′/C-4′, and ^2^*J*_CH_ H-4′/C-5′ and H-5′/C-4′. In the case of sugar residue 2, spin systems from H-1″ (*δ*_H_ 5.13) to H-5″ (*δ*_H_ 3.87) and the other corresponding to methylene H_2_-6″ protons (*δ*_H_ 3.85/3.64) were connected by HMBC ^2^*J*_CH_ H-5″/C-6″, and ^3^*J*_CH_ H_2_-6″/C-4″ and H-4″/C-6″ correlations. The chemical shifts of protons H-5′ (*δ*_H_ 3.78) and H-5″ (*δ*_H_ 3.87), and long-range couplings ^3^*J*_CH_ H-1′/C-5′, H-1″/C-5″ and H-5″/C-1″, confirmed both sugar residues as hexopyranoses. Correlations between H-3′ (*δ*_H_ 3.84)/C-1″ (*δ*_C_ 102.2) and H-1″ (*δ*_H_ 5.13)/C-3′ (*δ*_C_ 81.7) unambiguously confirmed the 1″ → 3′-glycosidic linkage between sugar residues.

Vicinal couplings between oxymethine H-2 (*δ*_H_ 3.80, *δ*_C_ 72.3) and diasterotopic oxymethylenes H_2_-1 (*δ*_H_ 3.77/3.42, *δ*_C_ 70.3) and H_2_-3 (*δ*_H_ 3.58/3.53, *δ*_C_ 64.4) indicated the presence of a glycerol unit, connected to the anomeric carbon C-1′ by observing ^3^*J*_CH_ H-1′/C-1 and H_2_-1/C-1′ correlations. The structure of the acyl moiety of testacoside A (**1**) was established as follows. The carbonyl carbon C1‴ at *δ*_C_ 175.7 showed ^2^*J*_CH_ and ^3^*J*_CH_ correlations with methylenes at *δ*_H_ 2.36 and *δ*_H_ 1.63, respectively, confirming the presence of fatty acid chain which, in agreement with the un-assigned signals in the ^13^C NMR spectrum, consisted of 15 carbon atoms. The HSQC spectrum showed a triplet methyl group (*δ*_H_ 0.88, *δ*_C_ 11.9) and a doublet methyl group (*δ*_H_ 0.86, *δ*_C_ 19.8), typical of a branched *anteiso* chain, corresponding to the 12-methyltetradecanoid acid. Finally, correlation of diasterotopic protons’ H_2_-6′ with the ester carbonyl carbon C-1‴ confirmed the connection of the fatty acid moiety at C6′.

Analysis of vicinal ^3^*J*_HH_ coupling constants of testacoside A (**1**) indicated large coupling constants of ca. 9.5 Hz between *J*_H2″-H3″_, *J*_H3″-H4″_, and *J*_H4″-H5″_, in addition to the *J*_H1″-H2″_ of 3.8 Hz, indicating that sugar 2 was an *α*-glucopyranose. The small coupling constant *J*_H1′-H2′_ of 1.6 Hz typical of an equatorial-equatorial arrangement established an *α*-anomeric configuration for the sugar moiety attached to the glycerol portion. However, the overlapping of ^1^H signals prevented the measurement of the other coupling constants, hampering to establish the relative configuration of this sugar residue. Then, testacoside A peracetate derivative **5** was prepared with the aim to resolve the ^1^H signals of the two sugar moieties (Table S1). The coupling constants *J*_H2′-H3′_ of 3.8 Hz, *J*_H3′-H4′_ of 9.7 Hz, and *J*_H4′-H5′_ of 10.0 Hz indicated an equatorial-axial, axial-axial, and axial-axial arrangement, respectively, which, in addition to the small coupling constant of the anomeric proton *J*_H1′-H2′_ of 1.5 Hz, typical of an equatorial-equatorial orientation, proved that the sugar residue connected to glycerol in **1** was *α*-mannopyranose. Analysis of NMR and HRESIMS data of testacoside A peracetate derivative **5** (Figs. S31-S37) confirmed the identification of **1** as 1-[*α*-glucopyranosyl-(1 → 3)-(6-*O*-acyl-*α*-mannopyranosyl)]-glycerol.

Analysis of the MS/MS data of **1** showed the fragment ions [M − glycerol]^+^ at *m/z* 549.3217, [M − glucose + H_2_O]^+^ at *m/z* 479.3220, [M − glucose]^+^ at *m/z* 461.3117, and [M − glucose − glycerol]^+^ at *m/z* 387.2766 (Fig. S8).

**Table 1:** ^1^H (600 MHz) and ^13^C (150 MHz) NMR data for testacosides A–C (**1**–**3**) in MeOH-*d*_*4*_

	Testacoside A (**1**)		Testacoside B (**2**)		Testacoside C (**3**)	
Position	*δ*_C_, type	*δ*_H_ (*J* in Hz)	*δ*_C_, type	*δ*_H_ (*J* in Hz)	*δ*_C_, type	*δ*_H_ (*J* in Hz)
Gly						
1	70.3, CH_2_	3.77 (m) 3.42 (m)	70.3, CH_2_	3.77 (m) 3.42 (m)	70.3, CH_2_	3.77 (m) 3.42 (m)
2	72.3, CH	3.80 (m)	72.3, CH	3.79 (m)	72.3, CH	3.79 (m)
3	64.4, CH_2_	3.58 (dd, 5.2–11.1) 3.53 (dd, 5.6–11.1)	64.4, CH_2_	3.58 (dd, 5.0–11.1) 3.52 (dd, 5.7–11.1)	64.4, CH_2_	3.58 (dd, 5.1–11.0) 3.53 (dd, 5.6–11.1)
Man						
1′	102.4, CH	4.74 (d 1.6)	102.4, CH	4.74 (d, 1.4)	102.4, CH	4.74 (d, 1.30)
2′	71.2, CH	4.12 (bt)	71.2, CH	4.12 (bt)	71.2, CH	4.12 (bt)
3′	81.7, CH	3.84 (m)	81.7, CH	3.84 (m)	81.6, CH	3.84 (m)
4′	67.7, CH	3.86 (m)	67.7, CH	3.86 (m)	67.7, CH	3.86 (m)
5′	72.3, CH	3.78 (m)	72.3, CH	3.78 (m)	72.3, CH	3.78 (m)
6′	65.0, CH_2_	4.40 (dd, 2.1–11.8) 4.23 (dd, 6.2–11.8)	64.9, CH_2_	4.40 (dd, 1.8–11.7) 4.23 (dd, 6.2–11.7)	65.0, CH_2_	4.40 (dd, 1.7–11.7) 4.23 (dd, 6.2–11–7)
Glu						
1″	102.2, CH	5.13 (d, 3.8)	102.2, CH	5.13, (d, 3.9)	102.2, CH	5.13 (d, 3.8)
2″	74.2, CH	3.41 (dd, 3.8–9.7)	74.2, CH	3.41 (dd, 3.8–9.5)	74.2, CH	3.41 (dd, 3.8–9.2)
3″	75.3, CH	3.70 (t, 9.3)	75.3, CH	3.69 (t, 9.3)	75.2, CH	3.70 (t, 9.3)
4″	72.1, CH	3.25 (t, 9.5)	72.0, CH	3.25 (t, 9.5)	72.0, CH	3.25 (t, 9.5)
5″	74.2, CH	3.87 (m)	74.2, CH	3.87 (m)	74.2, CH	3.87 (m)
6″	62.9, CH_2_	3.85 (m) 3.64 (dd, 6.7–11.9)	62.9, CH_2_	3.85 (m) 3.64 (dd, 6.7–11.9)	62.9, CH_2_	3.85 (m) 3.64 (dd, 6.7–12.0)
Aglyc						
1‴	175.7, C		175.7, C		175.7, C	
2‴	35.1, CH_2_	2.36 (t, 7.4)	35.1, CH_2_	2.36 (t, 7.4)	35.1, CH_2_	2.36 (t, 7.5)
3‴	26.2, CH_2_	1.63 (m)	26.2, CH_2_	1.63 (m)	26.2, CH_2_	1.63 (m)
4–10‴	28.3–31.2, CH_2_	1.30	28.7–31.2, CH_2_	1.29	28.7–31.2, CH_2_	1.29
11‴	37.9, CH_2_	1.30 1.10	30.4, CH_2_	1.32	30.4, CH_2_	1.34
12‴	35.8, CH	1.31	30.8, CH_2_	1.30	30.6, CH_2_	1.30
13‴	30.7, CH_2_	1.35 1.14	40.4, CH_2_	1.17	38.0, CH_2_	1.30 1.10
14‴	11.9, CH_3_	0.88 (t, 7.2)	29.3, CH	1.52	35.8, CH	1.30 (m)
15‴	19.8, CH_3_	0.86 (d 6.4)	23.8, CH_3_	0.88 (d, 6.7)	30.8, CH_2_	1.35 1.14
16‴			23.8, CH_3_	0.88 (d, 6.7)	11.9, CH_3_	0.88 (t, 7.4)
17‴					19.8, CH_3_	0.86 (d, 6.3)

Analysis of NMR data of testacosides B (**2**) and C (**3**) (Table 1, Figs. S9-S23), as well as of their respective peracetylated derivatives **6** and **7** (Table S1, Figs. S38-S50), indicated almost the same structural features to those of testacoside A, with subtle differences in their acyl moieties. In the case of testacoside B (**2**), the HSQC spectrum (Fig. S12) disclosed a methyl doublet (*δ*_H_ 0.88, *δ*_C_ 23.8) characteristic of a branched *iso* fatty acid chain, indicating its structure as 14-methylpentadecanoic acid. As for testacoside C (**3**) (Fig. S20), a methyl triplet (*δ*_H_ 0.88, *δ*_C_ 11.9) and a methyl doublet (*δ*_H_ 0.86, *δ*_C_ 19.8) were observed, indicating an *anteiso* 14-mehtylhexadecanoic acid. Testacoside B (**2**) displayed a [M + H]^+^ ion at *m/z* 655.3882 and a [M + Na]^+^ ion at *m/z* 677.3707 (Fig. S14), consistent with the molecular formula C_31_H_58_O_14_ according to its HRMS analysis. Testacoside C (**3**) presented the molecular formula C_32_H_60_O_14_ based on its HRMS analysis, which showed a [M + H]^+^ ion at *m/z* 669.4041 and an [M + Na]^+^ ion at *m/z* 691.3864 (Fig. S22). These results confirmed testacosides B and C as analogs of testacoside A with one and two additional CH_2_ carbons, respectively, in their fatty acid chains. Such structures were validated by analysis of HRMS/MS data, which showed the same fragmentation pattern of testacoside A with + 14 Da for testacoside B (**2**) (Fig. S15) and + 28 Da for and testacoside C (**3**) (Fig. S23).

Testacoside D (**4**) presented the molecular formula C_32_H_60_O_14_, determined from its [M + Na]^+^ ion at *m/z* 691.3864, isomeric to testacoside C (**3**) (Fig. S24). Analysis of its NMR data (Table 2, Figs. S25-S29) and of its peracetylated derivative **8** (Table S2, Figs. S51-S56) indicated the same sugar, glycerol, and fatty acid units. The strong long-range correlation ^3^*J*_CH_ H_2_-3/C-1‴ demonstrated that the fatty acid chain was connected to the C-3 position of the glycerol unit, instead of the C-6′ position of the mannose residue, establishing the structure of testacoside D (**4**) as 1-[*α*-glucopyranosyl-(1 → 3)-(*α*-mannopyranosyl)]-3-*O*-acylglycerol (Fig. 3). The chemical shift of the geminal protons H_2_-6′ (*δ*_H_ 3.84/3.73) indicated that C-6′ was not substituted. Analysis of the HRMS/MS spectrum confirmed the linkage of the fatty acid chain by means of the fragment ion [M − glucose − mannose]^+^ at *m/z* 345.3005 (Fig. S30).

**Table 2:** ^1^H (600 MHz) and ^13^C (150 MHz) NMR data for testacoside D (**4**) in MeOH-*d*_4_

	Testacoside D (**4**)	
Position	*δ*_C_, type	*δ*_H_ (*J* in Hz)
Gly		
1	69.8, CH_2_	3.78 (dd, 4.8–10.3) 3.46 (dd, 6.2–10.3)
2	69.6, CH	3.98 (m)
3	66.6, CH_2_	4.16 (dd, 4.4–11.4) 4.08 (dd, 6.0–11.4)
Man		
1′	102.3, CH	4.77 (d, 1.8)
2′	71.3, CH	4.12 (dd, 1.9–2.9)
3′	82.0, CH	3.82 (m)
4′	67.5, CH	3.85(m)
5′	74.9, CH	3.59 (m)
6′	62.9, CH_2_	3.84 (m) 3.73 (dd, 5.7–11.8)
Glu		
1″	102.2, CH	5.11 (d, 3.9)
2″	74.2, CH	3.41 (dd, 3.9–9.8)
3″	75.3, CH	3.69 (t, 9.4)
4″	72.1, CH	3.24 (dd, 9.1–9.8)
5″	74.2, CH	3.88 (m)
6″	62.9, CH_2_	3.86 (m) 3.64 (dd, 6.5–11.6)
Aglyc		
1‴	175.6, C	
2‴	35.1, CH_2_	2.36 (t, 7.6)
3‴	26.1, CH_2_	1.62 (m)
4‴	30.4, CH_2_	1.33
5–12‴	28.4–31.2, CH_2_	1.29
13‴	38.0, CH_2_	1.30 1.10
14‴	35.8, CH	1.30 (m)
15‴	30.8, CH_2_	1.35 1.14
16‴	11.9, CH_3_	0.88 (t, 7.4)
17‴	19.8, CH_3_	0.86 (d, 6.6)

### Determination of the absolute configuration of sugar residues of 2 and 4

**Fig. 4:**
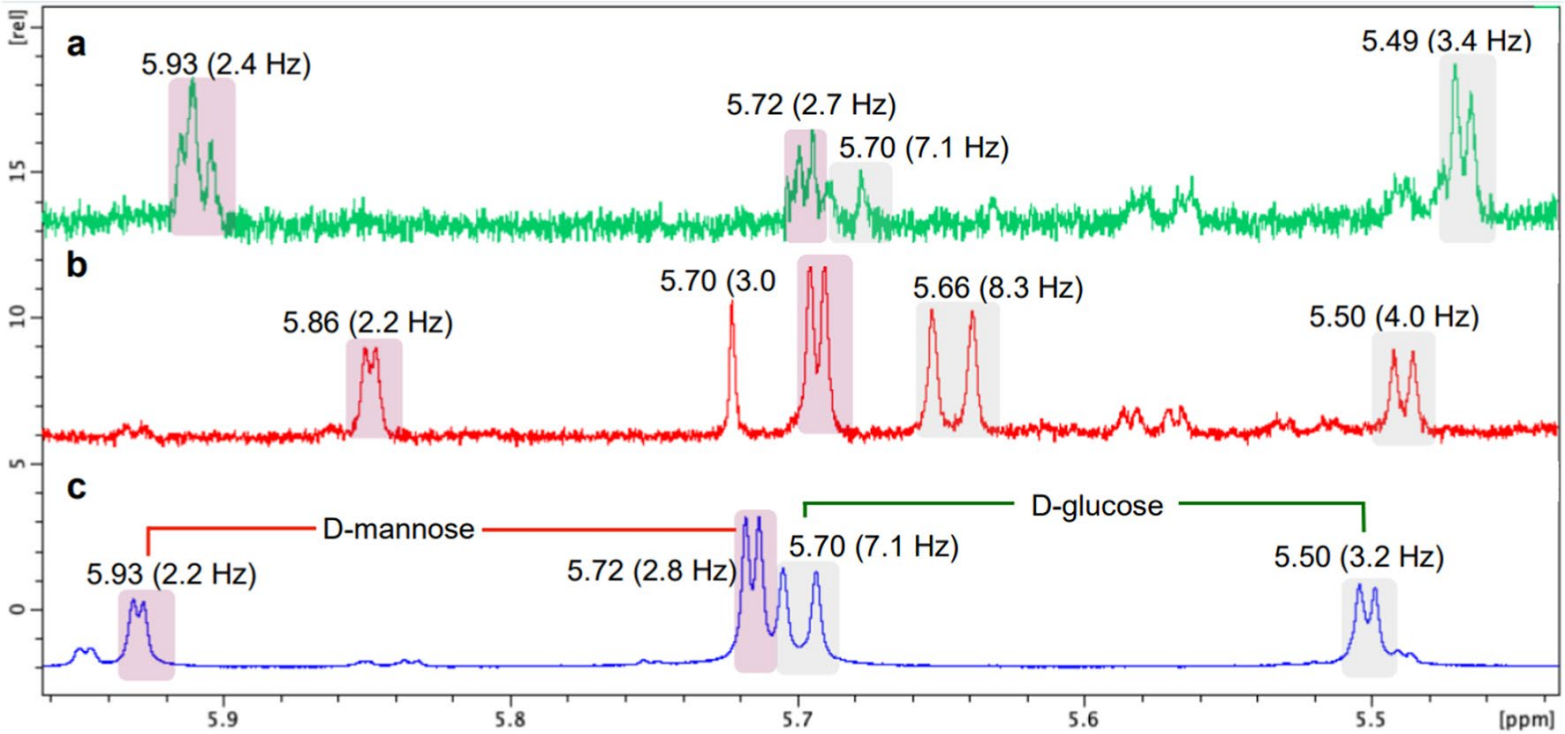
^1^H NMR spectra (600 MHz, in pyridine-*d*_5_) of thiazolidine derivatives of **2** (**a**), **4** (**b**) and the mixture of D-mannose and D-glucose standards (**c**)

A recent method reported the determination of the absolute stereochemistry of sugar enantiomers by ^1^H NMR (Inagaki et al. 2021). In applying this procedure, acid hydrolysis of testacosides B (**2**) and D (**4**) yielded the free monosaccharides, which were subsequently reacted with L-cysteine methyl ester hydrochloride, directly in the NMR tube in pyridine-*d*_5_, to give their corresponding thiazolidine derivatives. The ^1^H-NMR measured chemical shifts and *J*_HH_ coupling constants of the relevant enantio-dependent proton were consistent with those of the enantiomerically pure D-glucose and D-mannose thiazolidine derivatives (Fig. 4). The absolute configuration for testacosides A (**1**) and C (**3**) could not be established by this method because their corresponding thiazolidine derivatives’ sugar moieties degraded during the ^1^H NMR analysis, for no obvious reason. However, considering that NMR data of sugar moieties of **1** and **3** were practically identical to those of **2** and **4**, as well as similar values of optical rotation were recorded, we propose the D-configuration for both sugar residues of **1** and **3**.

## Discussion

Testacosides A–C were inactive against *P. falciparum* at concentration of 10 μM, as well as when tested in antimicrobial activity against a panel of multi-resistant ESKAPE bacterial strains at concentration of 60 μM.

Testacosides A–D (**1**–**4**) are similar to glycoglycerolipids isolated from cultures of the gram-negative bacterium *Flavobacterium marinotypicum* (Yagi and Maruyama 1999) and from cultures of the sponge associated bacterium *Microbacterium* sp. (Wicke et al. 2000a, b). Reports of secondary metabolites produced by *Microbacterium* spp. are scarce and include the cytotoxic peptaibols microbacterins A and B produced in culture by the deep sea bacterium *M. sediminis* sp. nov. YLB-01(T) (Liu et al. 2015). The α- and γ-pyrone micropyrones A and B produced in culture by an endophytic *Microbacterium* sp. did not display antibacterial activity against *S. aureus* and methicillin-resistant *S. aureus* (Xu et al. 2021). Other studies reported the production of carotenoids by *Microbacterium* spp. (Reis-Mansur et al. 2019; Mandakovic et al. 2020).

Glycoglycerolipids related to testacosides have been isolated from cultures of a wide variety of bacteria obtained from different sources, such as the pathogen *Rothia mucilaginosa* (Pasciak et al. 2004), *Bacillus pumilus* (Ramm et al. 2004), *Microccocus luteus* (Pakkiri et al. 2004), and the marine-derived *Bacillus licheniformis* 09IDYM23 (Tareq et al. 2015). Unusual glycoglycerolipids diacylated at both 3 and 6′ positions have already been isolated from bacteria. Such are the cases of lutoside from the sponge-associated bacterium *M. luteus* (Bultel-Poncé, et al. 1995) and the polar glycolipid 1-[*α*-mannopyranosyl-(1 → 3)-(6-O-acyl-*α*-mannopyranosyl)]-3-*O*-acylglycerol from *Arthrobacter atrocyaneus* (Niepel, et al. 1997). Glycoglycerolipids were also reported from cyanobacteria (Shirahashi et al. 1993), algae (Morimoto, et al. 1995), lichens (Sassaki et al. 1999), and marine organisms (Cheng-Sánchez and Sarabia 2018).

The glycoglycerolipid di-*O*-12-methyl-tetradecanoyl-3- *O*-beta-d-galactopyranosyl-sn-glycerol has been previously reported from *Microbacterium* sp. M874 and has been related to avoid cell damage by radicalar oxygen and heat (Nakata 2000). Lipids comprising of 1-*O*-acyl-3-[α-glucopyranosyl-(1–3)-(6-*O*-acyl-α-mannopyranosyl)]glycerol connected to 14-methyl-hexadecanoic acid and 12-methyl-tetradecanoic acid moieties at C-6 of the mannose unit and glycerol have been isolated from cultures of *Microbacterium* sp. DSM 12583 obtained from the marine sponge *Halichondria panicea* (Wicke et al 2000a, b). Antiviral activity against herpes simplex virus types 1 and 2 have been reported for synthetic monoglycosyl diglycerides (Janwitayanuchit et al. 2003) as well as very mild antimicrobial activity (Cateni et al 2007, 2008). The chemical structure of glycoglycerolipids significantly impacts the biological activity and functions of these compounds, which are of biological and medical importance (Pagano et al. 2016). Glycoglycerolipids are some of the most abundant glycolipids in plants, animals, and bacteria, exerting essential roles in cell membranes and as chemical protecting agents against biological pathogens and physical damage (Jala et al. 2022). Thus, glycoglycerolipids, such as testacosides, are of considerable biological importance for maintenance of cell integrity under healthy and stressed conditions, being biotechnologically relevant agents such as biosurfactants, biomaterials, and biologically active compounds (Orive-Milla et al 2020).

In summary, a series of four new glycoglycerolipids were isolated from cultures produced by *T. brasiliensis*-associated bacterium *M. testaceum* (Figure S57). Dereplication with molecular networking along with biological activity results enabled us to direct the isolation of these compounds, demonstrating the feasibility of combining chemical and biological information at the early stages of the biodiscovery process. This is the first report of secondary metabolites produced in culture by a *M. testaceum* species.

## Supplementary Information

Below is the link to the electronic supplementary material.

Supplementary file1 (PDF 1828 KB)

## Data Availability

All data generated and analyzed are included in the supplementary information file.
