# Supplementary material for: Testacosides A–D, glycoglycerolipids produced by Microbacterium testaceum isolated from Tedania brasiliensis
Source: Appl Microbiol Biotechnol. 2024 Jan 12;108:112. doi: 10.1007/s00253-023-12870-0 (PMC10786734; doi:10.1007/s00253-023-12870-0)
Supplement: Supplementary file 1 [file 253_2023_12870_MOESM1_ESM.pdf]

Supplementary Material

for

Testacosides A-D, glycoglycerolipids produced by *Microbacterium testaceum* isolated from *Tedania brasiliensis*

Jairo I. Quintana-Bulla,[1] Luciane A. C. Tonon,[1] Lamonielli F. Michaliski,[1] Eduardo Hajdu,[2] Antonio G. Ferreira,[3] Roberto G. S. Berlinck[1]

[1]Instituto de Química de São Carlos, Universidade de São Paulo, CP 780, CEP 13560-970, São Carlos, SP, Brazil

[2]Museu Nacional, Universidade Federal do Rio de Janeiro, Quinta da Boa Vista, s/n, CEP 20940-040, Rio de Janeiro, RJ, Brazil

[3]Departamento de Química, Universidade Federal de São Carlos, CEP 13565-905, São Carlos, SP, Brazil

✉ Roberto G. S. Berlinck rgsberlinck@iqsc.usp.br

Table of contents

Table S1 ^{1}H (600 MHz) and ^{13}C (150 MHz) NMR data for testacosides A-C peracetate derivatives (**5-7**) in $CDCl_3$

Table S2 ^{1}H (600 MHz) and ^{13}C (150 MHz) NMR data for testacoside D peracetate derivative (**8**) in $CDCl_3$

Figure S1 HRESIMS spectrum of testacoside A (**1)**

Figure S2 ^{1}H NMR spectrum of **1** (MeOH-d_4; 600 MHz)

Figure S3 ^{13}C NMR spectrum of **1** (MeOH-d_4; 150MHz)

Figure S4 DEPT-135 spectrum of **1** (MeOH-d_4; 150MHz)

Figure S5 HSQC spectrum of **1** (MeOH-d_4; 600 MHz)

Figure S6 COSY spectrum of **1** (MeOH-d_4; 600 MHz)

Figure S7 HMBC spectrum of **1** (MeOH-d_4; 600 MHz) HRESIMS spectrum of **1**

Figure S8 MS fragmentation spectrum of **1**

Figure S9 ^{1}H NMR spectrum of testacoside B (**2**) (MeOH-d_4; 600 MHz)

Figure S10 ^{13}C NMR spectrum of **2** (MeOH-d_4; 150 MHz)

Figure S11 COSY spectrum of **2** (MeOH-d_4; 600 MHz)

Figure S12 HSQC spectrum of **2** (MeOH-d_4; 600 MHz)

Figure S13 HMBC spectrum of **2** (MeOH-d_4; 600 MHz)

Figure S14 HRESIMS spectrum of **2**

Figure S15 MS fragmentation spectrum of **2**

Figure S16 ^{1}H NMR spectrum of testacoside C (**3**) (MeOH-d_4; 600 MHz)

Figure S17 ^{13}C NMR spectrum of **3** (MeOH-d_4; 150 MHz)

Figure S18 DEPT-135 spectrum of **3** (MeOH-d_4; 150 MHz)

Figure S19 COSY spectrum of **3** (MeOH-d_4; 600 MHz)

Figure S20 HSQC spectrum of **3** (MeOH-d_4; 600 MHz)

Figure S21 HMBC spectrum of **3** (MeOH-d_4; 600 MHz)

Figure S22 HRESIMS spectrum of **3**

Figure S23 MS fragmentation spectrum of **3**

Figure S24 HRESIMS spectrum of testacoside D (**4**)

Figure S25 ^{1}H NMR spectrum of **4** (MeOH-d_4; 600 MHz)

Figure S26 ^{13}C NMR spectrum of **4** (MeOH-d_4; 150MHz)

Figure S27 COSY spectrum of **4** (MeOH-d_4; 600 MHz)

Figure S28 HSQC spectrum of **4** (MeOH-d_4; 600 MHz)

Figure S29 HMBC spectrum of **4** (MeOH-d_4; 600 MHz)

Figure S30 MS fragmentation spectrum of **4**

Figure S31 ^{1}H NMR spectrum of testacoside A peracetate (**5**) ($CDCl_3$; 600 MHz)

Figure S32 ^{13}C NMR spectrum of **5** ($CDCl_3$; 150 MHz)

Figure S33 DEPT-135 spectrum of **5** ($CDCl_3$; 150 MHz)

Figure S34 COSY spectrum of **5** ($CDCl_3$; 600 MHz)

Figure S35 HSQC spectrum of **5** ($CDCl_3$; 600 MHz)

Figure S36 HMBC spectrum of **5** ($CDCl_3$; 600 MHz)

Figure S37 HRESIMS spectrum of **5**

Figure S38 ^{1}H NMR spectrum of testacoside B peracetate (**6**) ($CDCl_3$; 600 MHz)

Figure S39 ^{13}C NMR spectrum of **6** ($CDCl_3$; 150 MHz)

Figure S40 DEPT-135 spectrum of **6** ($CDCl_3$; 150 MHz)

Figure S41 COSY spectrum of **6** ($CDCl_3$; 600 MHz)

Figure S42 HSQC spectrum of **6** ($CDCl_3$; 600 MHz)

Figure S43 HMBC spectrum of **6** ($CDCl_3$; 600 MHz)

Figure S44 ^{1}H NMR spectrum of testacoside C peracetate (**7**) ($CDCl_3$; 600 MHz)

Figure S45 ^{13}C NMR spectrum of **7** ($CDCl_3$; 150 MHz)

Figure S46 DEPT-135 spectrum of **7** ($CDCl_3$; 150 MHz)

Figure S47 COSY spectrum of **7** ($CDCl_3$; 600 MHz)

Figure S48 HSQC spectrum of **7** ($CDCl_3$; 600 MHz)

Figure S49 HMBC spectrum of **7** ($CDCl_3$; 600 MHz)

Figure S50 HRESIMS spectrum of **7**

Figure S51 ^{1}H NMR spectrum of testacoside D peracetate (**8**) ($CDCl_3$; 600 MHz)

Figure S52 ^{13}C NMR spectrum of **8** ($CDCl_3$; 150 MHz)

Figure S53 DEPT-135 spectrum of **8** ($CDCl_3$; 150 MHz)

Figure S54 COSY spectrum of **8** ($CDCl_3$; 600 MHz)

Figure S55 HSQC spectrum of **8** ($CDCl_3$; 600 MHz)

Figure S56 HMBC spectrum of **8** ($CDCl_3$; 600 MHz)

Figure S57 Neighbour-joining tree based on partial 16S rRNA gene sequences (1063 nt) showing relationships between isolate *Microbacterium testaceum* **J55** and closely related *Microbacterium* species. Asterisks indicate branches of the tree that were also found using the maximum-parsimony and minimum-evolution tree-making algorithms. Numbers at the nodes are percentage bootstrap values based on 1,000 resampled datasets. Bar 0.002 substitutions per nucleotide position.

Table S1 ^{1}H (600 MHz) and ^{13}C (150 MHz) NMR data for testacosides A-C peracetate derivatives (**5-7**) in $CDCl_3$

	Testacoside A peracetate (**5**)		Testacoside B peracetate (**6**)		Testacoside C peracetate (**7**)	
Position	δ_C, type	δ_H (J in Hz)	δ_C, type	δ_H (J in Hz)	δ_C, type	δ_H (J in Hz)
Gly						
1	66.4, CH_2	3.80 (dd, 4.6-10.9)<br>3.64 (dd, 6.0-10.9)	66.4, CH_2	3.80 (dd, 5.1-11.1)<br>3.64 (dd, 6.0-11.1)	66.4, CH_2	3.80 (dd, 4.8-11.0)<br>3.64 (dd, 6.2-11.0)
2	70.0, CH	5.21 (m)	70.0, CH	5.21 (m)	70.0, CH	5.21 (m)
3	62.4, CH_2	4.32 (dd, 4.1-12.0)<br>4.15 (dd, 5.8-11.9)	62.4, CH_2	4.32 (dd, 4.1-12.0)<br>4.15 (dd, 5.8-12.0)	62.4, CH_2	4.32 (dd, 4.0-12.3)<br>4.14 (dd, 6.0-12.1)
Man						
1’	97.8, CH	4.84 (d, 1.5)	97.8, CH	4.84 (bs)	97.8, CH	4.84 (bs)
2’	70.6, CH	5.20 (dd, 1.6-3.8)	70.6, CH	5.21 (m)	70.6, CH	5.20 (dd, 1.4-3.5)
3’	72.7, CH	4.18 (dd, 3.6-9.7)	72.7, CH	4.18 (dd, 3.6-9.8)	72.7, CH	4.18 (dd, 3.7-9.7)

4’	68.2, CH	5.29 (t, 10.0)	68.3, CH	5.30 (t, 9.9)	68.2, CH	5.29 (t, 10.0)
5’	69.1, CH	3.83 (m)	69.1, CH	3.84 (m)	69.1, CH	3.83 (m)
6’	62.4, CH_2	4.19 (dd, 5.6-12.2)<br>4.08 (dd, 2.8-12.3)	62.4, CH_2	4.19 (m)<br>4.08 (m)	62.4, CH_2	4.19 (dd, 5.6-12.3)<br>4.08 (dd, 2.3-12.6)
Glu						
1’’	96.7, CH	5.26 (d, 3.8)	96.7, CH	5.26 (d, 3.6)	96.7, CH	5.26 (d, 3.7)
2’’	70.9, CH	4.78 (dd, 3.6-9.7)	71.0, CH	4.78 (dd, 3.7-10.3)	70.9, CH	4.78 (dd, 3.8-10.4)
3’’	69.4, CH	5.35 (t, 10.0)	69.4, CH	5.35 (t, 9.9)	69.4, CH	5.35 (t, 10.0)
4’’	68.2, CH	5.04 (t, 9.9)	68.2, CH	5.04 (t, 9.9)	68.2, CH	5.04 (t, 9.9)
5’’	68,4, CH	4.10 (m)	68.4, CH	4.10 (m)	68.4, CH	4.10 (m)
6’’	61.9, CH_2	4.30 (dd, 4.4-12.3)<br>4.05 (dd, 2.3-12.3)	61.9, CH_2	4.31 (dd, 4.1-12.5)<br>4.06 (dd, 1.9-12.5)	61.9, CH_2	4.30 (dd, 4.3-12.4)<br>4.05 (dd, 2.0-12.4)

Aglyc						
1’’’	173.6, C		173.6, C		173.6, C	
2’’’	34.2, CH_2	2.35 (m)	34.2, CH_2	2.35 (m)	34.2, CH_2	2.35 (m)
3’’’	25.0, CH_2	1.64 (q, 7.4)	25.0, CH_2	1.63 (m)	25.0, CH_2	1.63 (m)
4’’’	29.4, CH_2	1.32 (m)	29.4, CH_2	1.32	29.3, CH_2	1.32
5-10’’’	27.3-30.2, CH_2	1.26	27.6-31.2, CH_2	1.26	27.6-31.2, CH_2	1.26
11’’’	36.8, CH_2	1.26<br>1.08	30.3, CH_2	1.31	30.4, CH_2	1.31
12’’’	34.6, CH	1.30 (m)	30.7, CH_2	1.30	30.8, CH_2	1.30
13’’’	29.8, CH_2	1.33<br>1.12	39.2, CH_2	1.16 (m)	36.8, CH_2	1.28<br>1.08
14’’’	11.6, CH_3	0.86 (t, 7.3)	28.2, CH	1.52 (m)	34.6, CH	1.30 (m)
15’’’	19.4, CH_3	0.84 (d, 6.3)	22.8, CH_3	0.87 (d, 6.9)	30.0, CH_2	1.33<br>1.13

16’’’	22.8, CH_3	0.87 (d, 6.9)	11.6, CH_3	0.86
17’’’			19.4, CH3	0.84

Table S2 ^{1}H (600 MHz) and ^{13}C (150 MHz) NMR data for testacoside D peracetate derivative (**8**) in $CDCl_3$

Testacoside D peracetate (**8**)		
Position	δ_C, type	δ_H (*J* in Hz)
Gly		
1	66.6, CH_2	3.81 (dd, 4.3-10.9)<br>3.65 (dd, 6.0-10.9)
2	70.1, CH	5.21 (m)
3	62.2, CH_2	4.33 (dd, 4.1-12.0)<br>4.15 (dd, 5.9-12.0)
Man		
1'	97.9, CH	4.84 (d, 1.32)
2'	70.6, CH	5.21 (m)
3'	72.7, CH	4.18 (dd, 3.6-9.7)
4'	68.2, CH	5.30 (t, 10.0)
5'	69.0, CH	3.84 (m)
6'	62.6, CH_2	4.21 (dd, 5.6-12.3)<br>4.06 (dd, 2.7-12-3)
Glu		
1''	96.7, CH	5.26 (d, 3.8)
2''	71.0, CH	4.78 (dd, 3.8-10.3)
3''	69.4, CH	5.35 (t, 10.0)
4''	68.2, CH	5.04 (t, 10.0)
5''	68.4, CH	4.10 (m)
6''	61.9, CH_2	4.30 (dd, 4.4-12.4)<br>4.05 (dd, 2.3-12.4)

Aglyc		
1’’’	173.4, C	
2’’’	34.2, CH_2	2.32
3’’’	25.0, CH_2	1.62
4’’’	29.3, CH_2	1.32
5-12’’’	28.4-31.2, CH_2	1.29
13’’’	37.3, CH_2	1.27<br>1.08
14’’’	34.6, CH	1.30
15’’’	29.7, CH_2	1.34<br>1.13
16’’’	11.6, CH_3	0.86
17’’’	19.4, CH_3	0.84

Figure S1 HRESIMS spectrum of testacoside A (**1**)

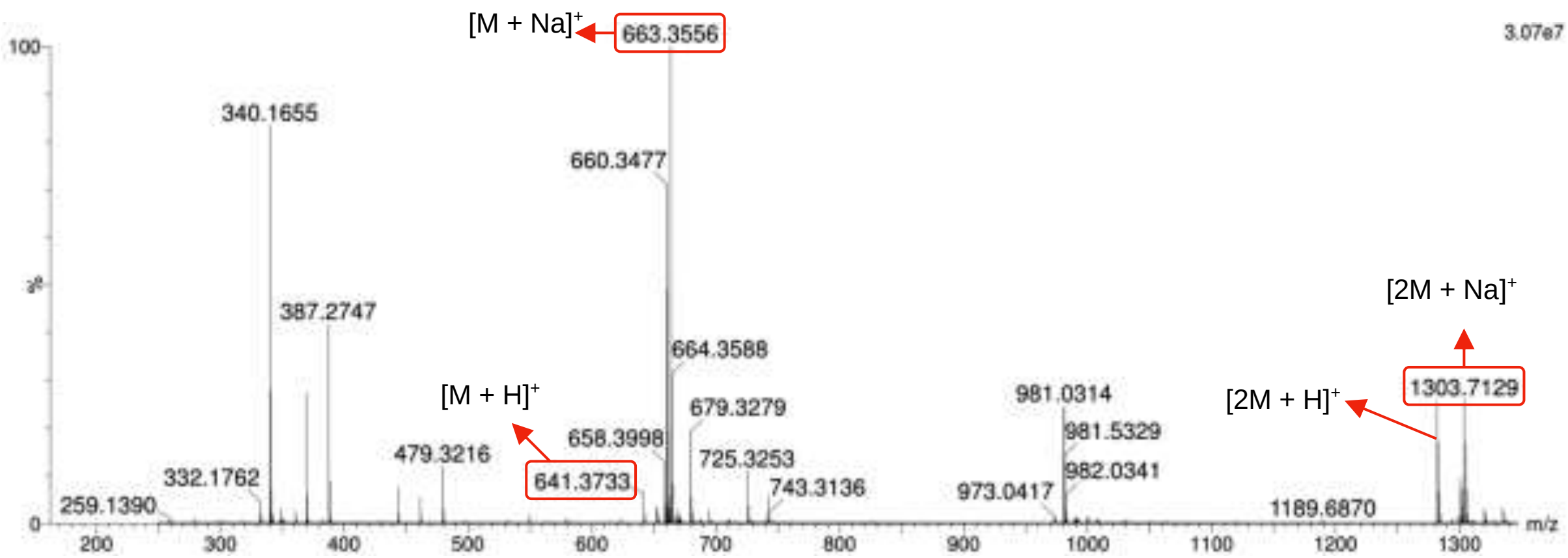

Figure S2 ^{1}H NMR spectrum of **1** (MeOH-d_4; 600 MHz)

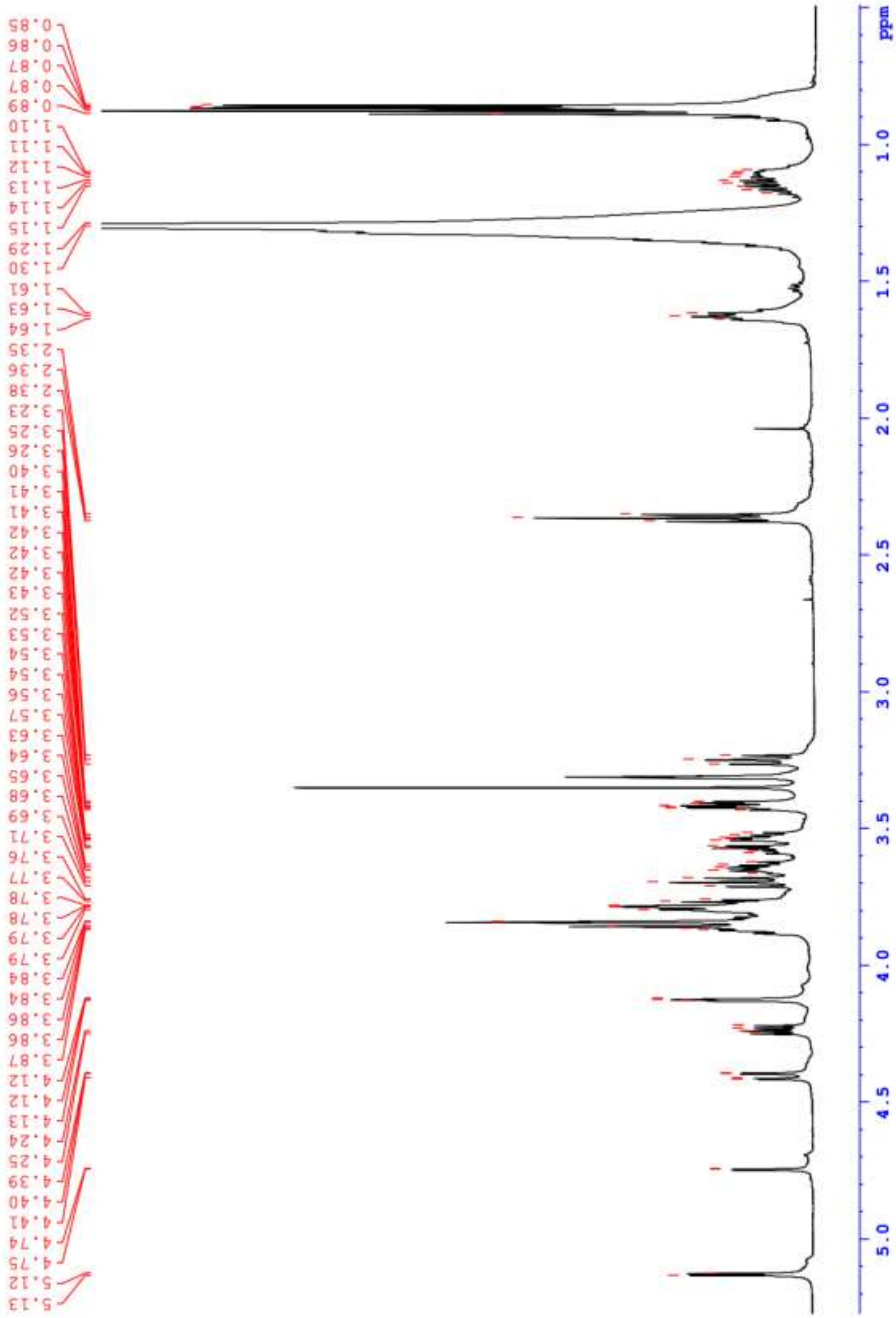

Figure S3 ^{13}C NMR spectrum of **1** (MeOH-d_4; 150 MHz)

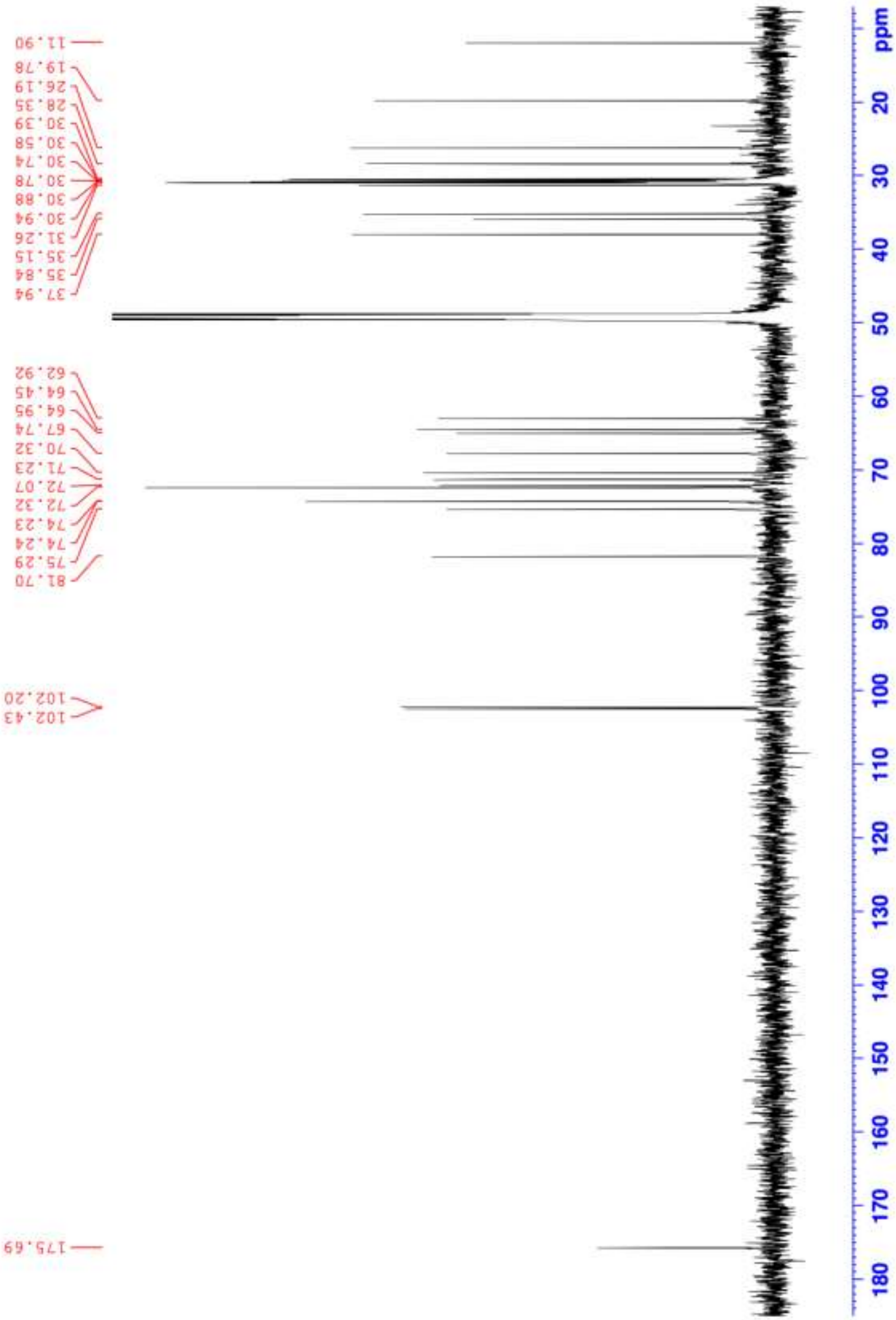

Figure S4 DEPT-135 spectrum of **1** (MeOH-d_4; 150 MHz)

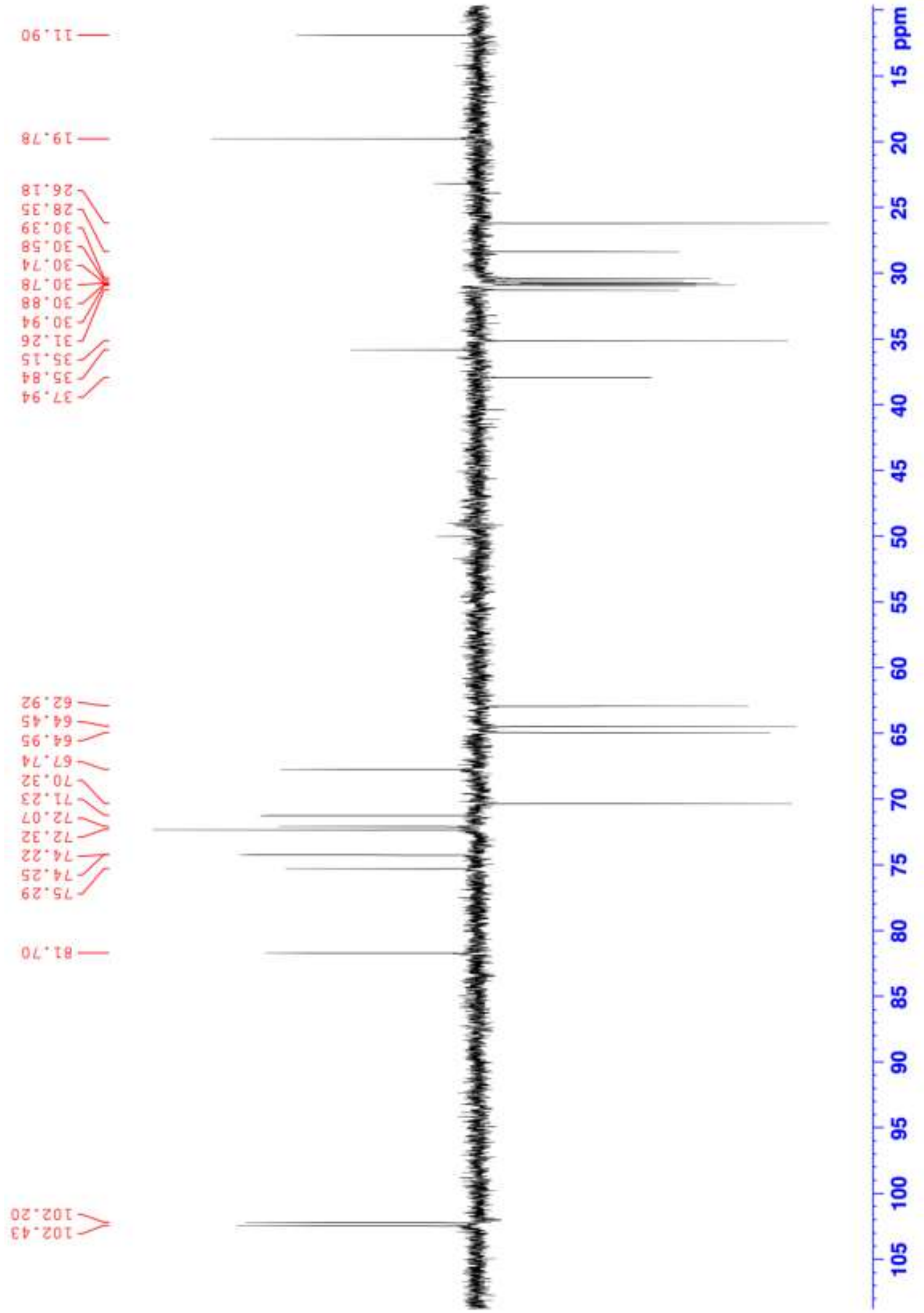

Figure S5 HSQC spectrum of **1** (MeOH-d_4; 600 MHz)

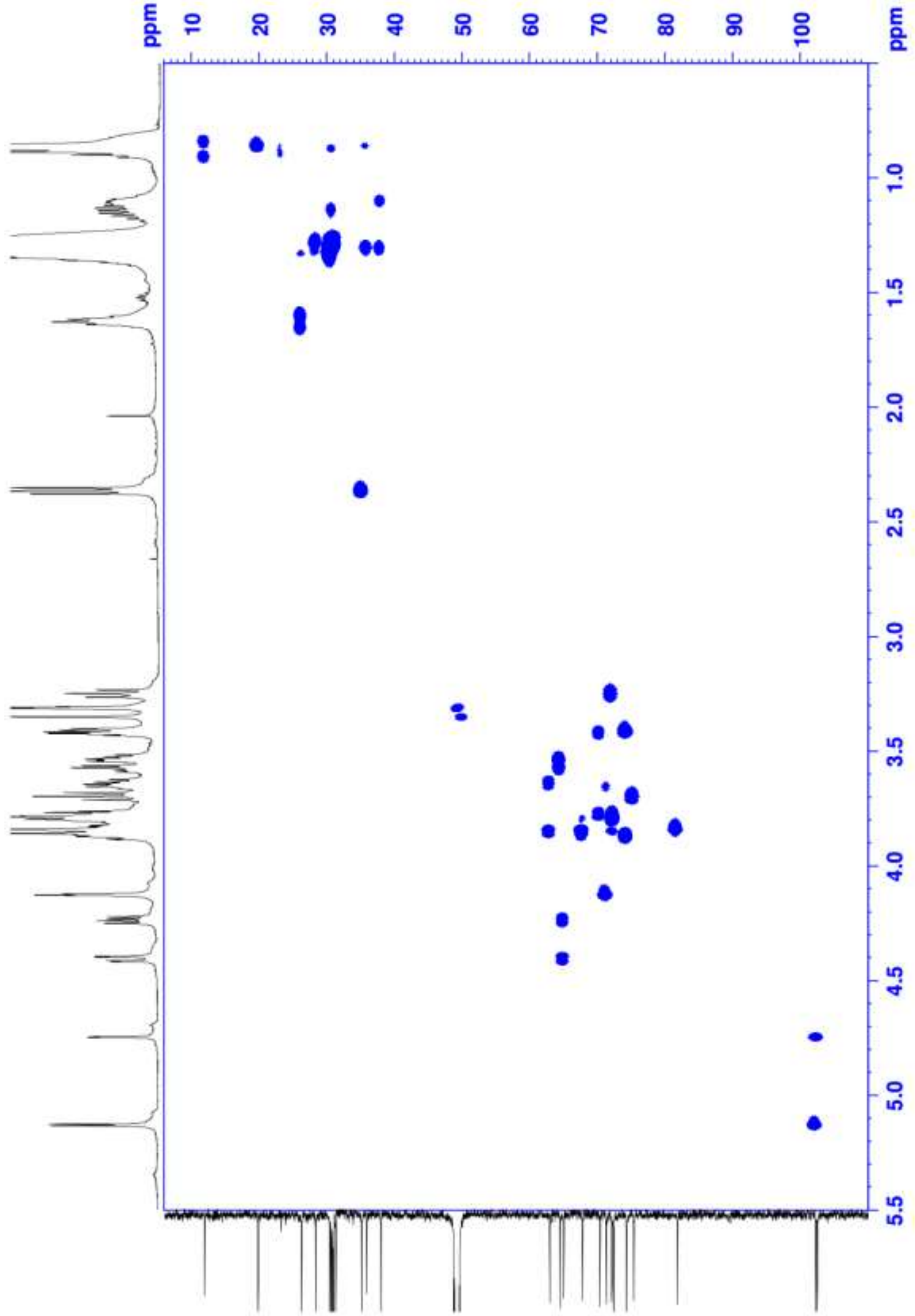

Figure S6 COSY spectrum of **1** (MeOH-d_4; 600 MHz)

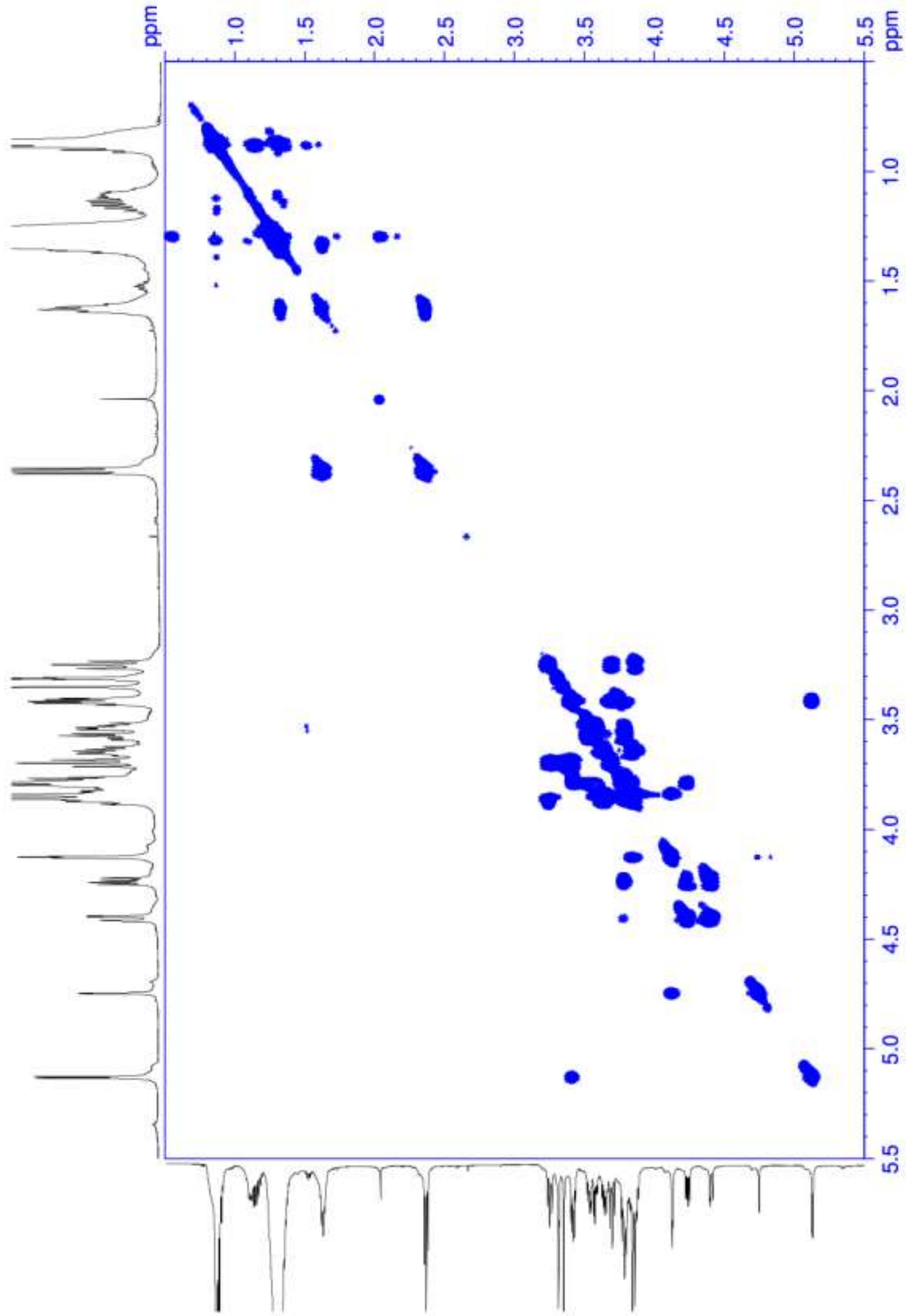

Figure S7 HMBC spectrum of **1** (MeOH-d_4; 600 MHz)

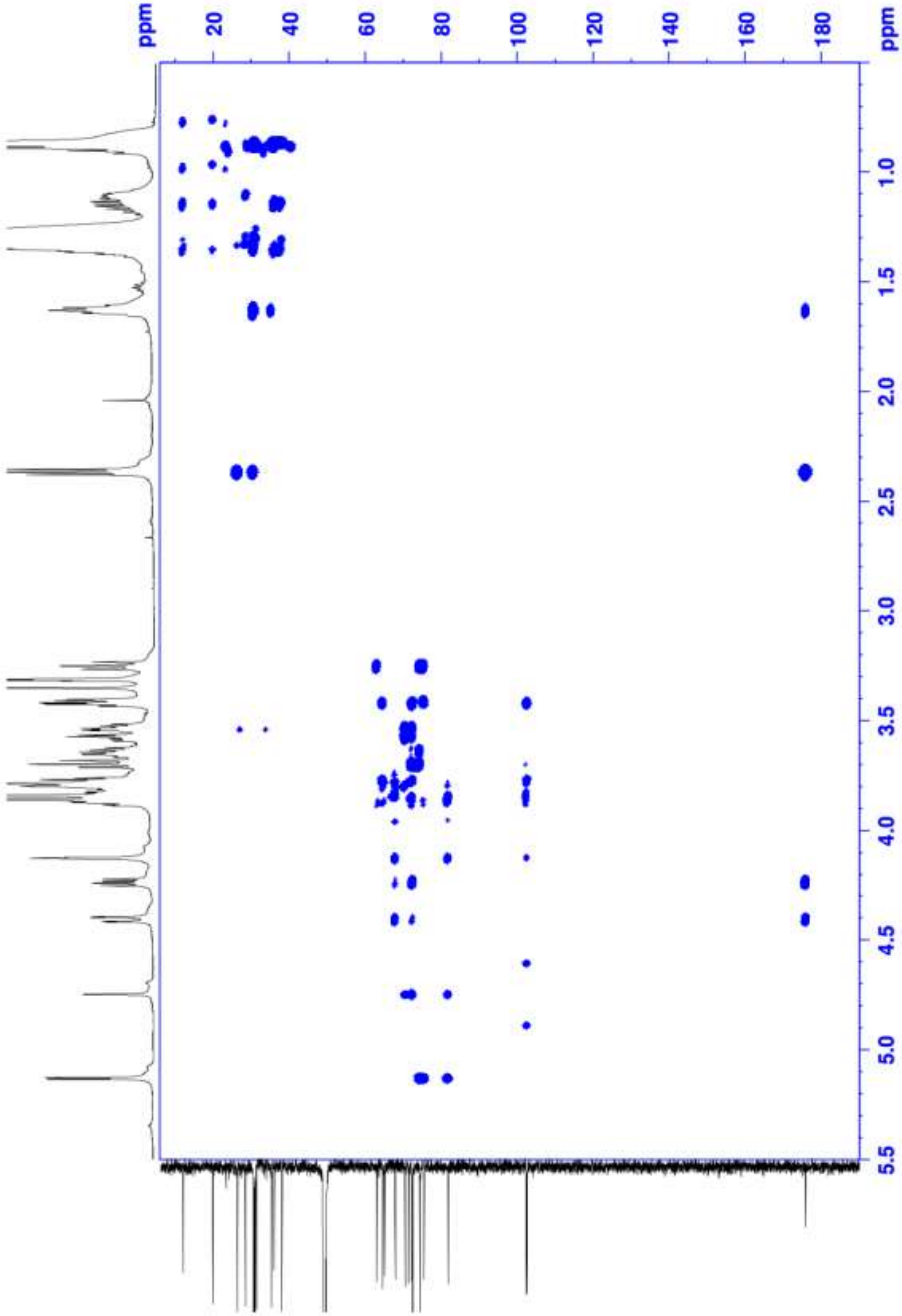

Figure S8 MS fragmentation spectrum of **1**

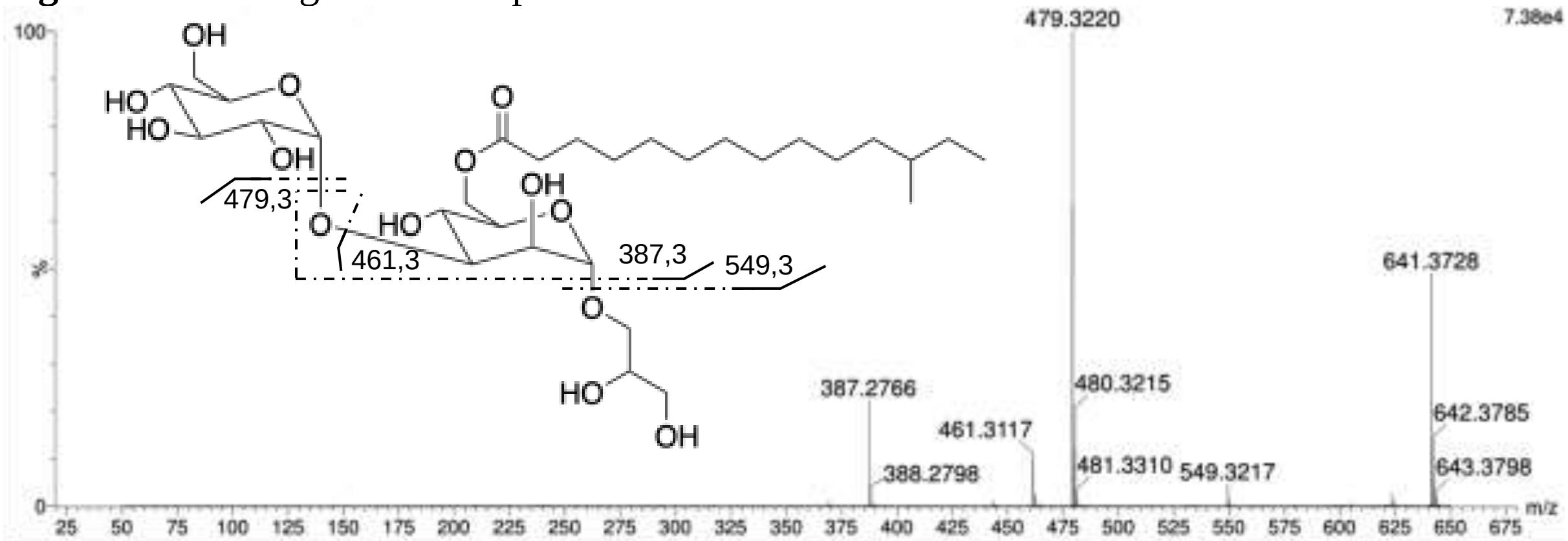

Figure S9 ^{1}H NMR spectrum of testacoside B (**2**) (MeOH-d_4; 600 MHz)

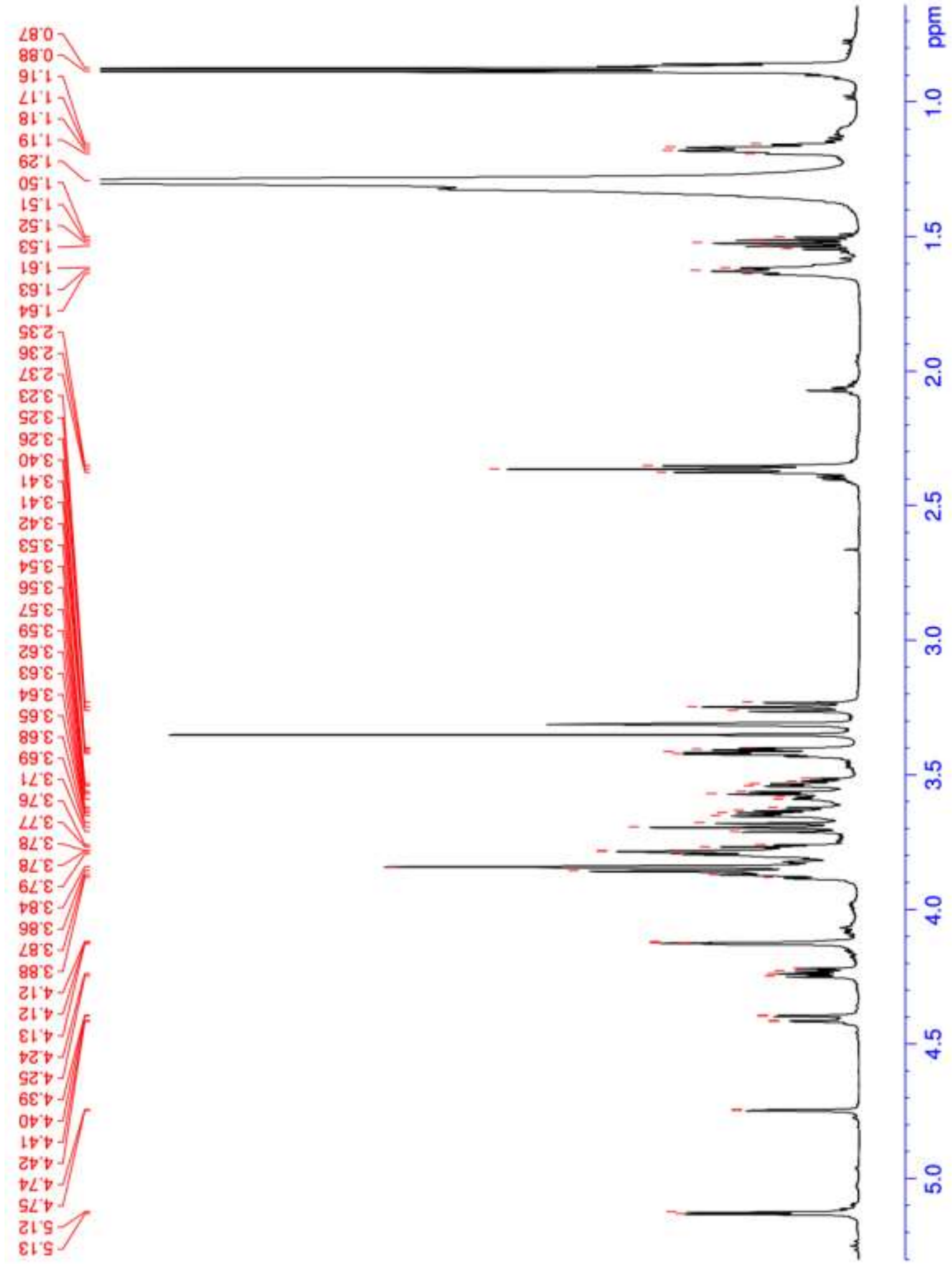

Figure S10 ^{13}C NMR spectrum of **2** (MeOH-d_4; 150 MHz)

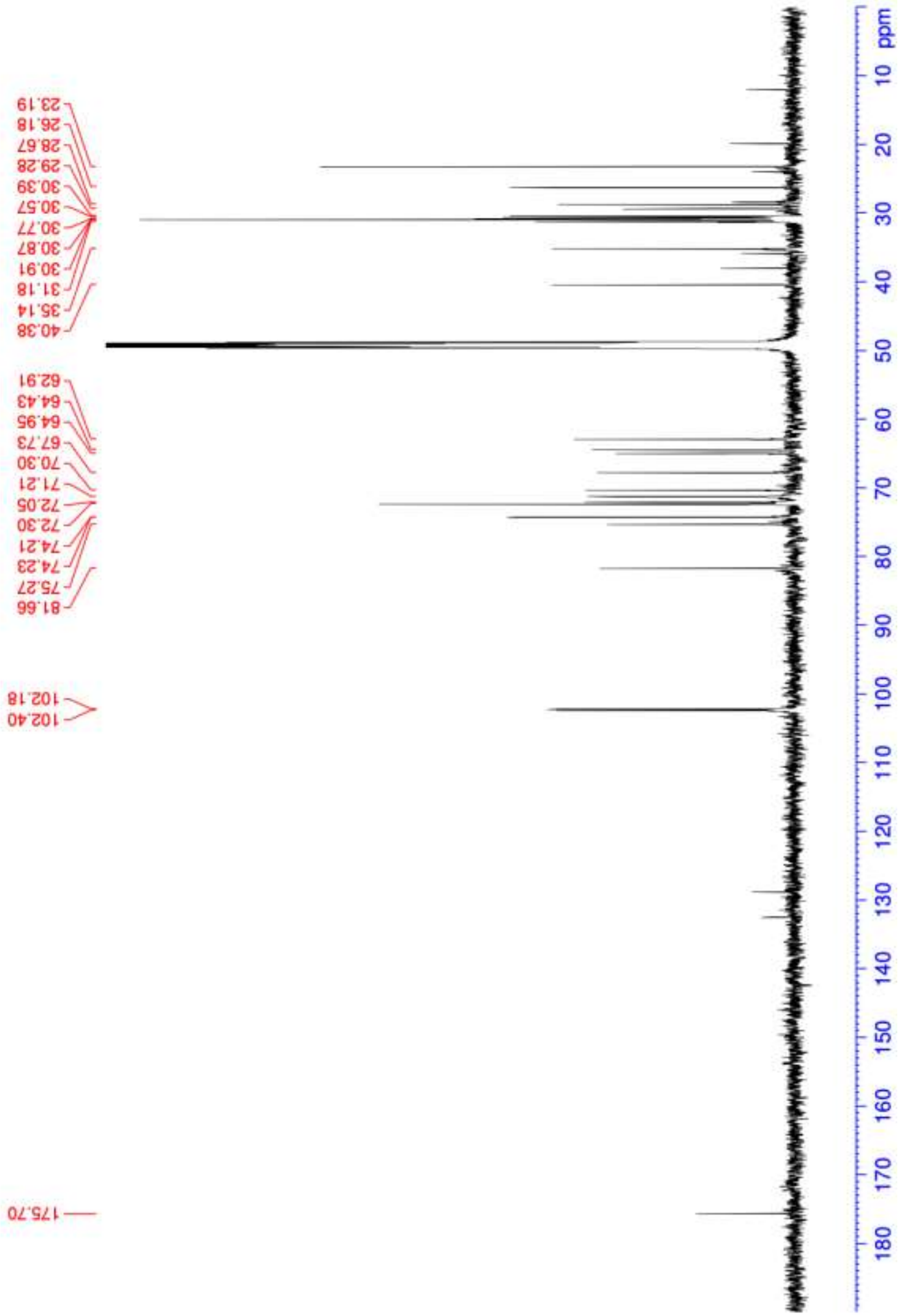

Figure S11 COSY spectrum of **2** (MeOH-d_4; 600 MHz)

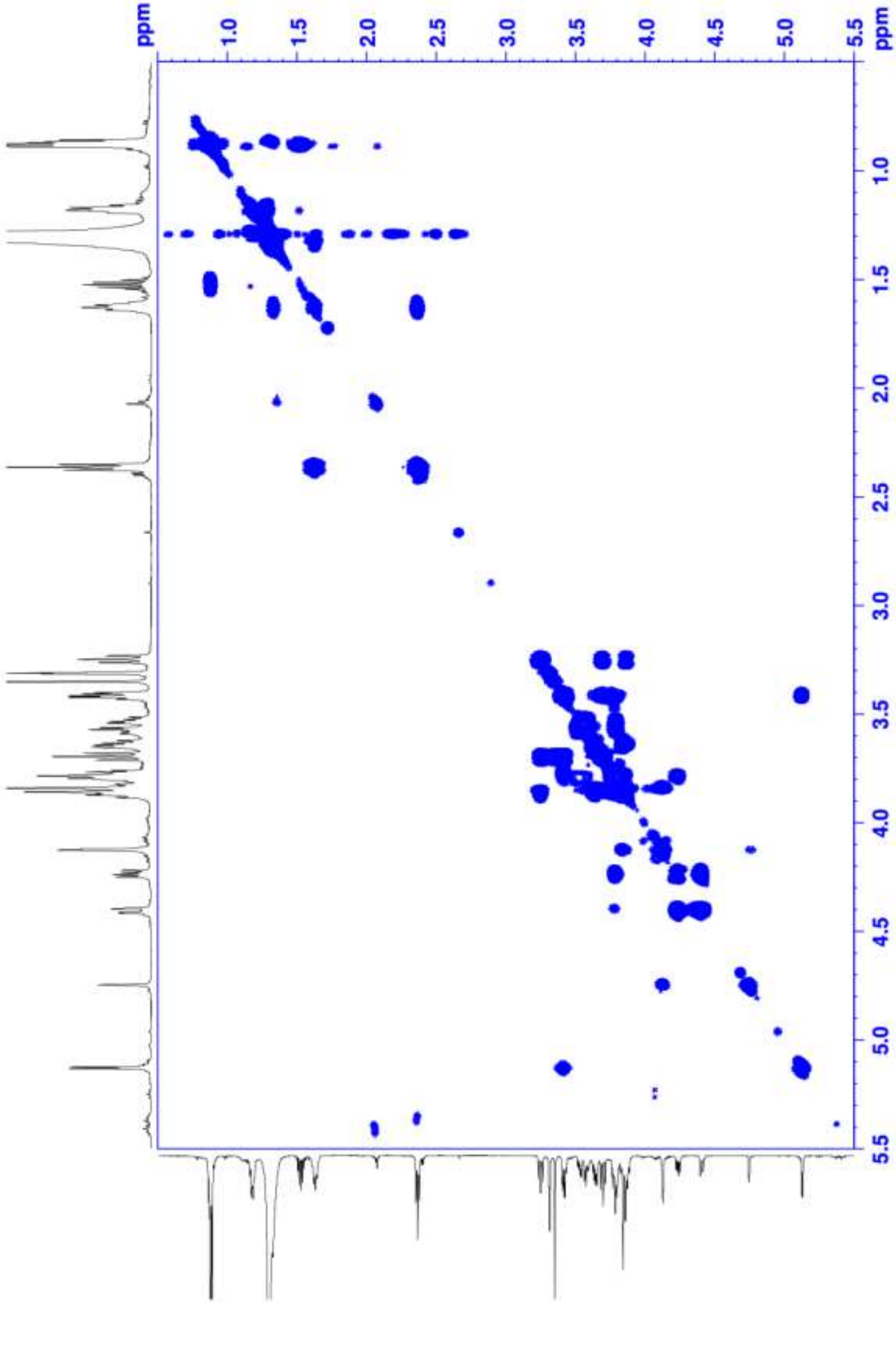

Figure S12 HSQC spectrum of **2** (MeOH-d_4; 600 MHz)

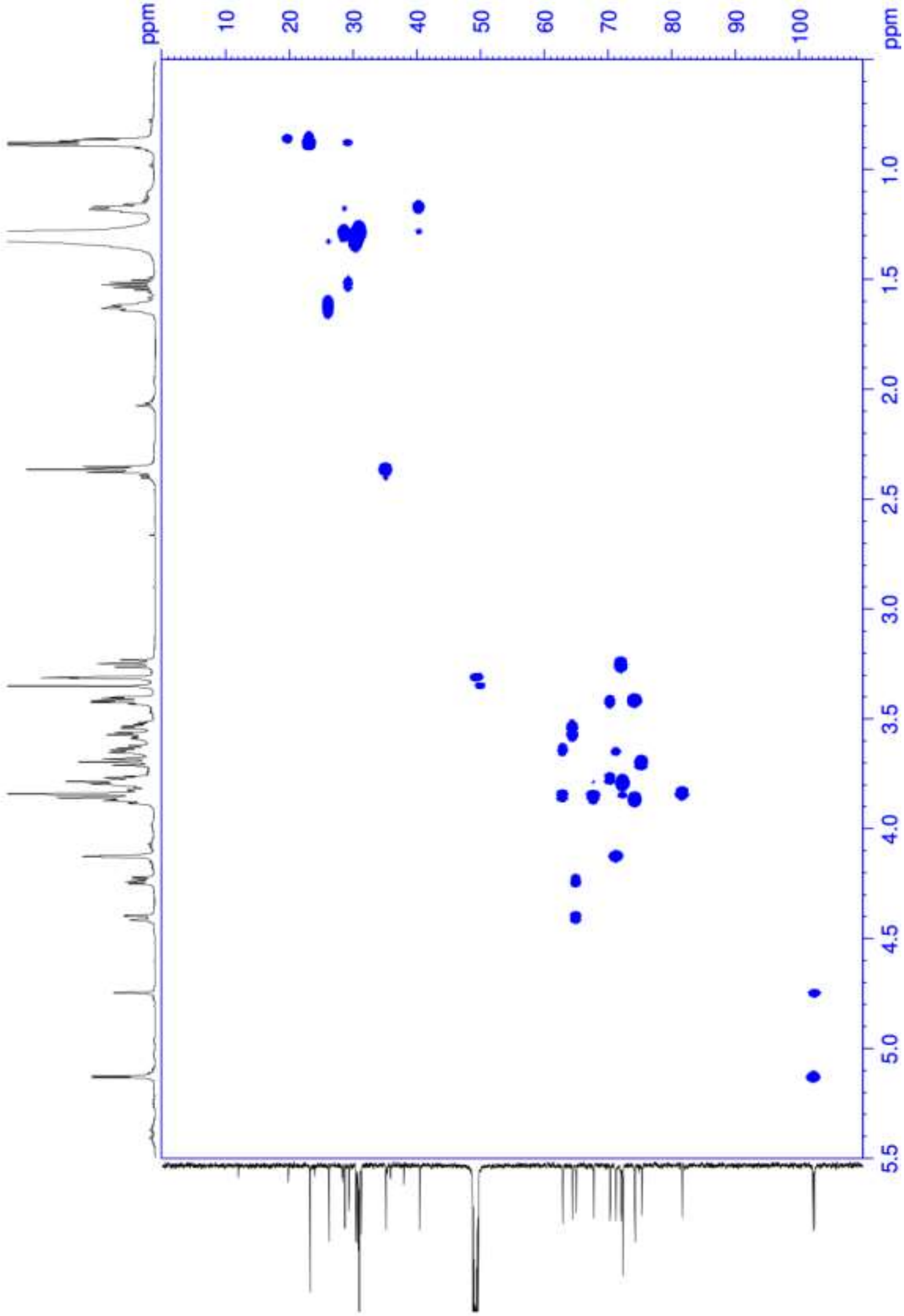

Figure S13 HMBC spectrum of **2** (MeOH-d_4; 600 MHz)

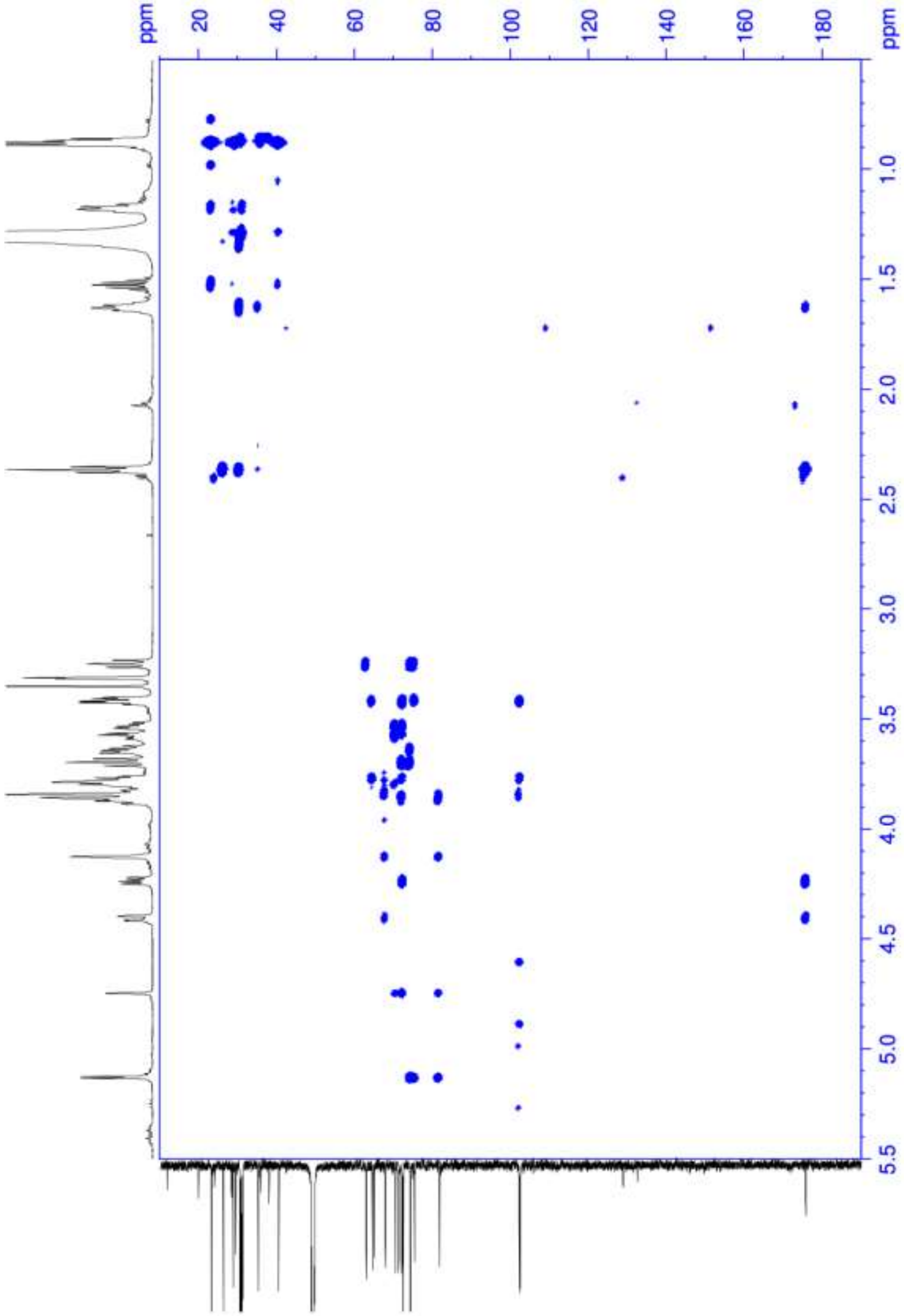

Figure S14 HRESIMS spectrum of **2**

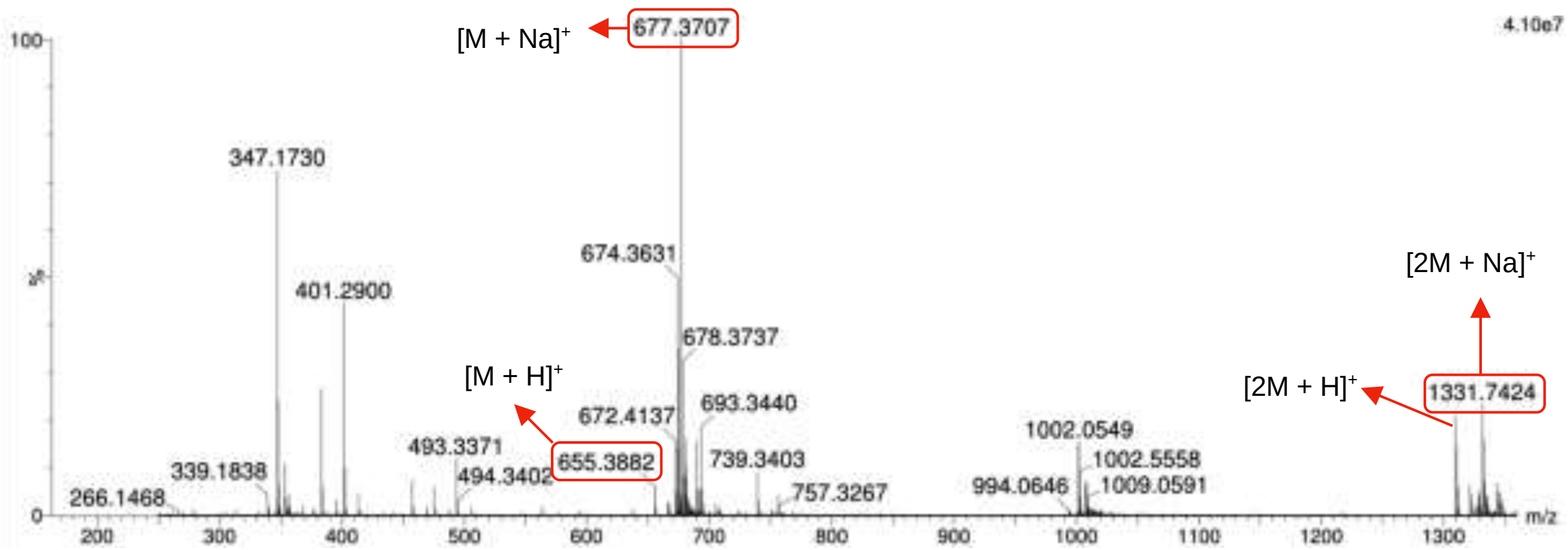

Figure S15 MS fragmentation spectrum of **2**

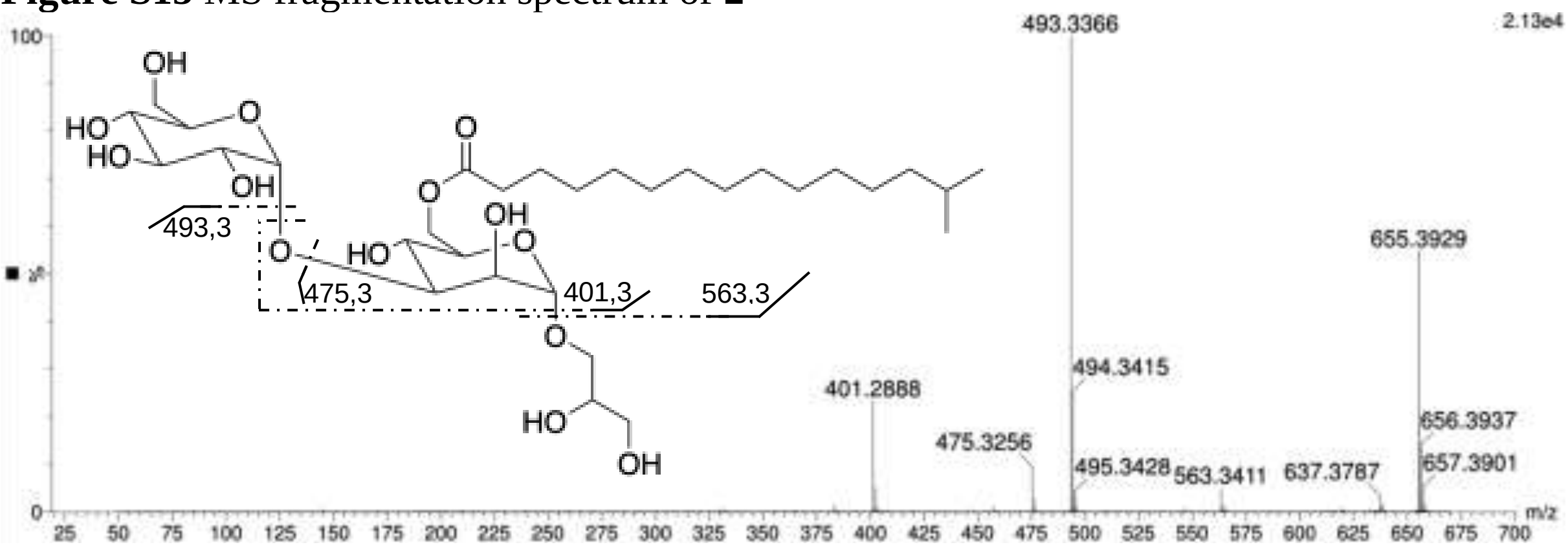

Figure S16 ^{1}H NMR spectrum of testacoside C (**3**) (MeOH-d_4; 600 MHz)

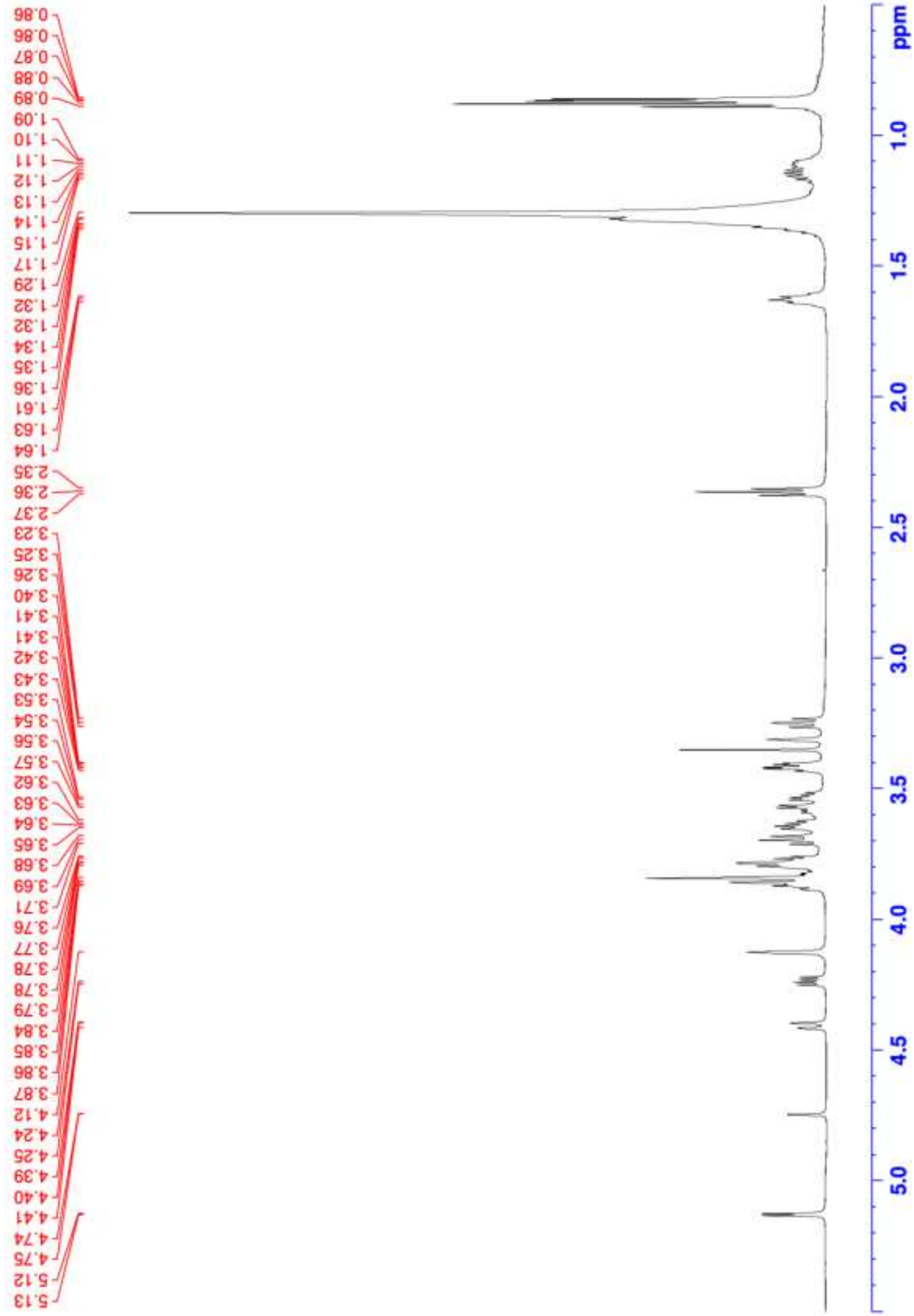

Figure S17 ^{13}C NMR spectrum of **3** (MeOH-d_4; 150 MHz)

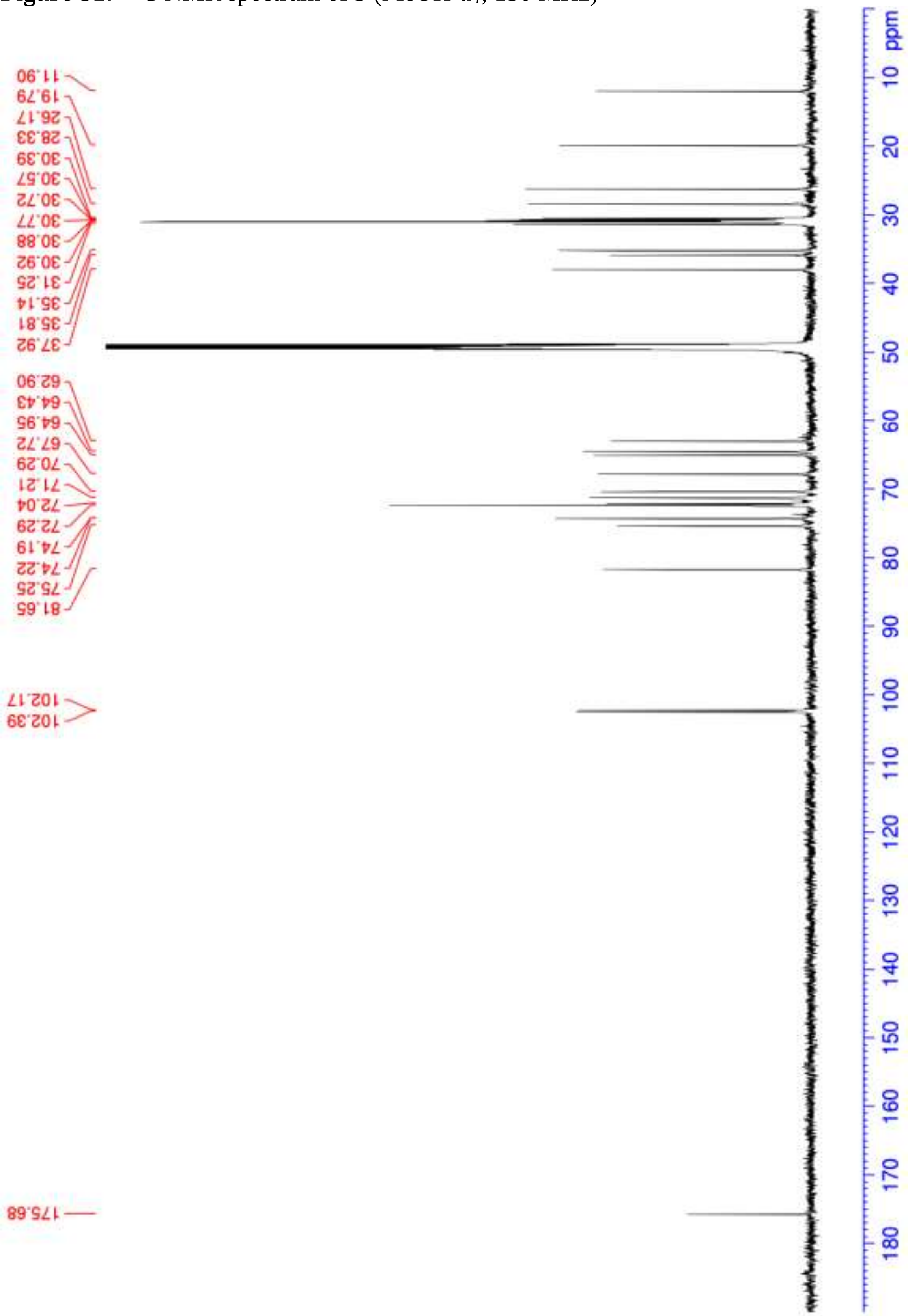

Figure S18 DEPT-135 spectrum of **3** (MeOH-d_4; 150 MHz)

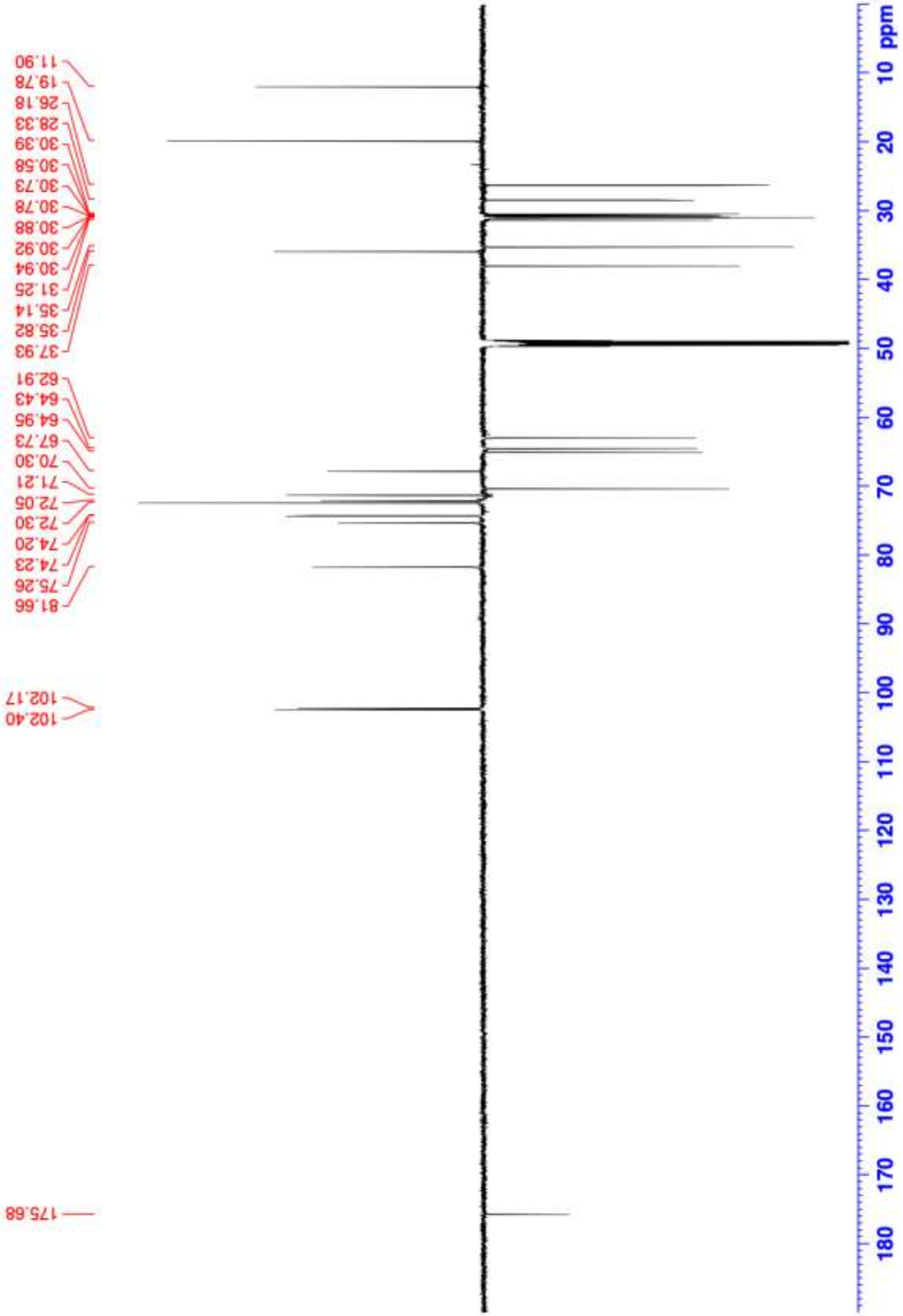

Figure S19 COSY spectrum of **3** (MeOH-d_4; 600 MHz)

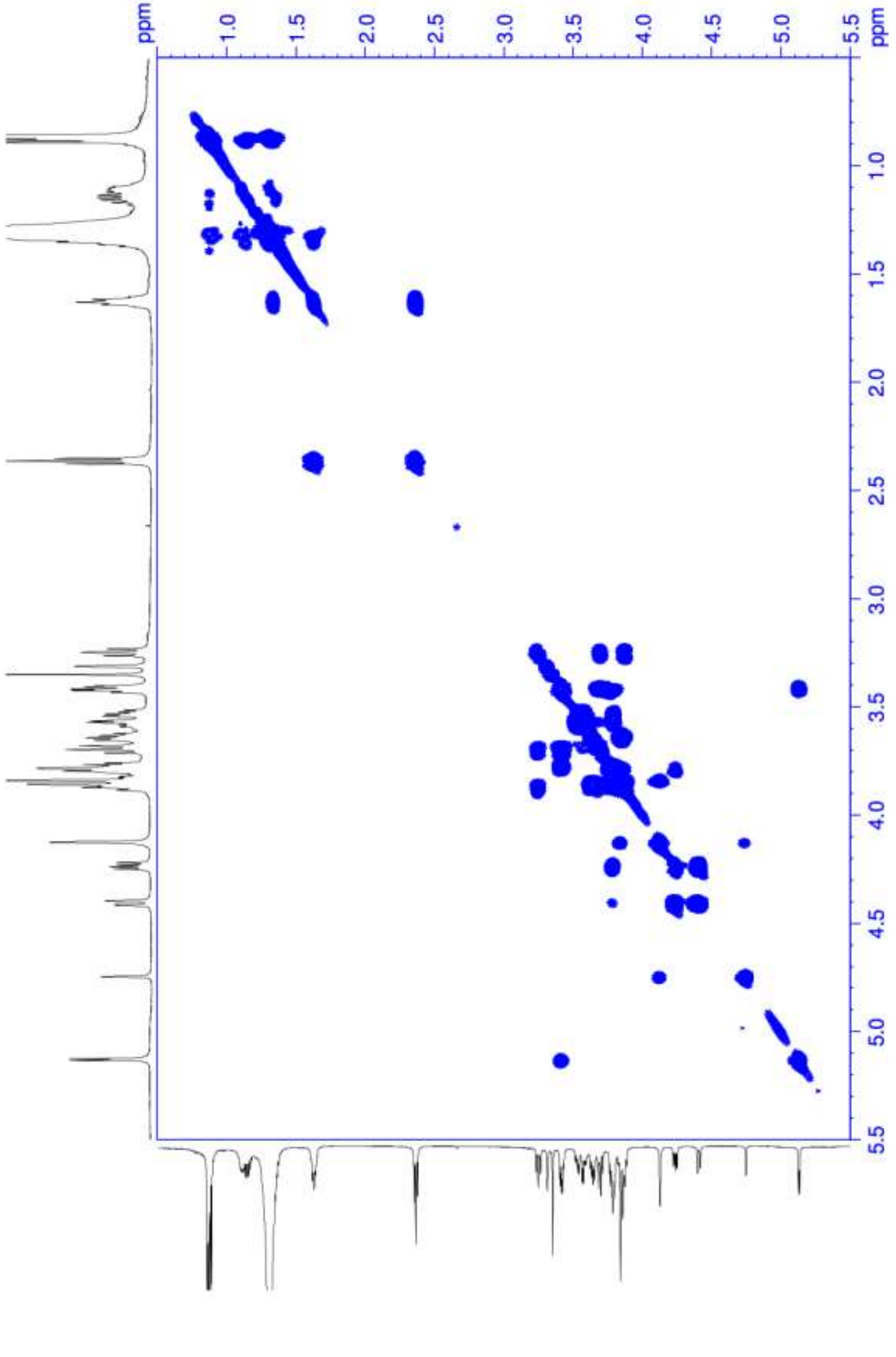

Figure S20 HSQC spectrum of **3** (MeOH-d_4; 600 MHz)

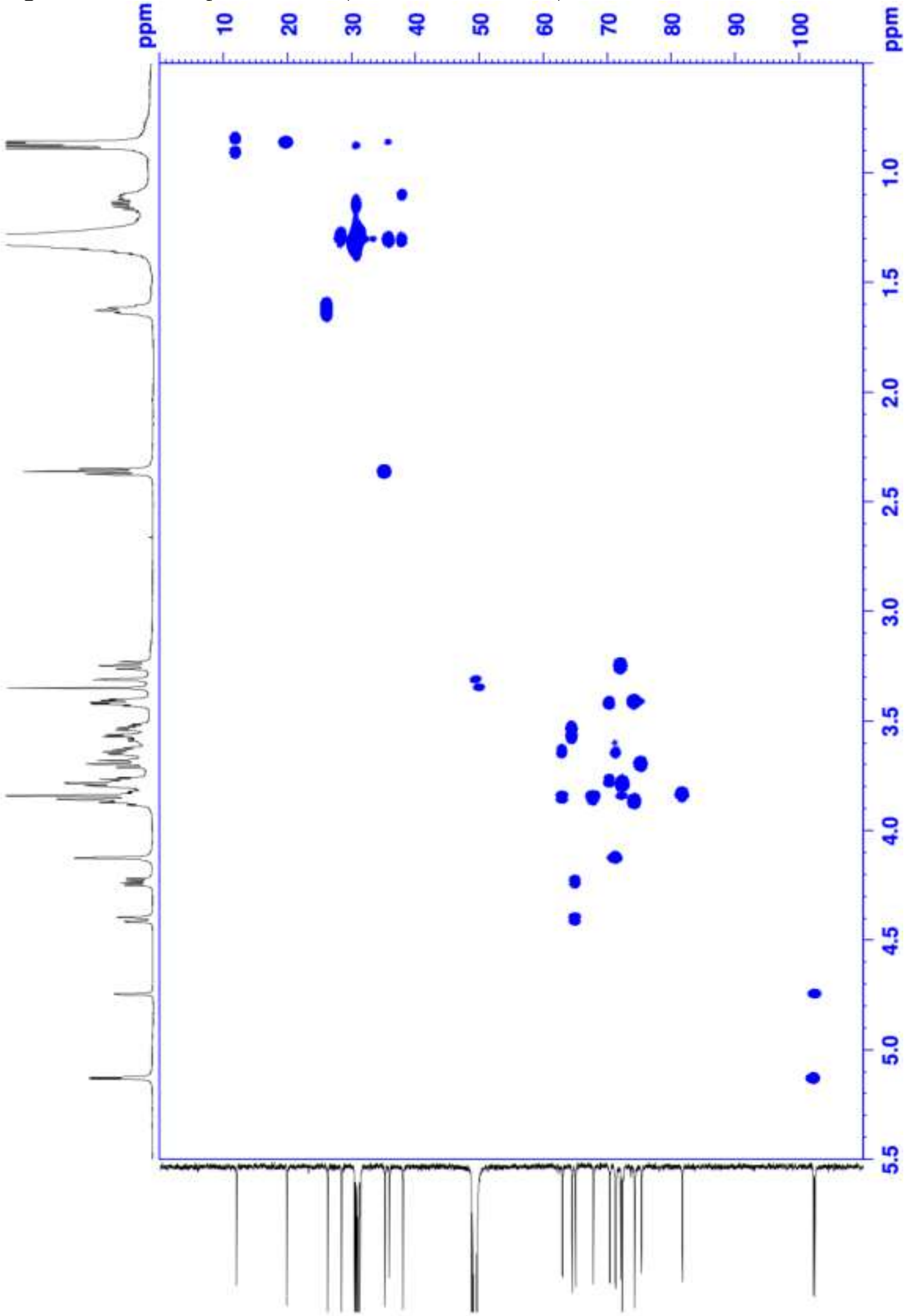

Figure S21 HMBC spectrum of **3** (MeOH-d_4; 600 MHz)

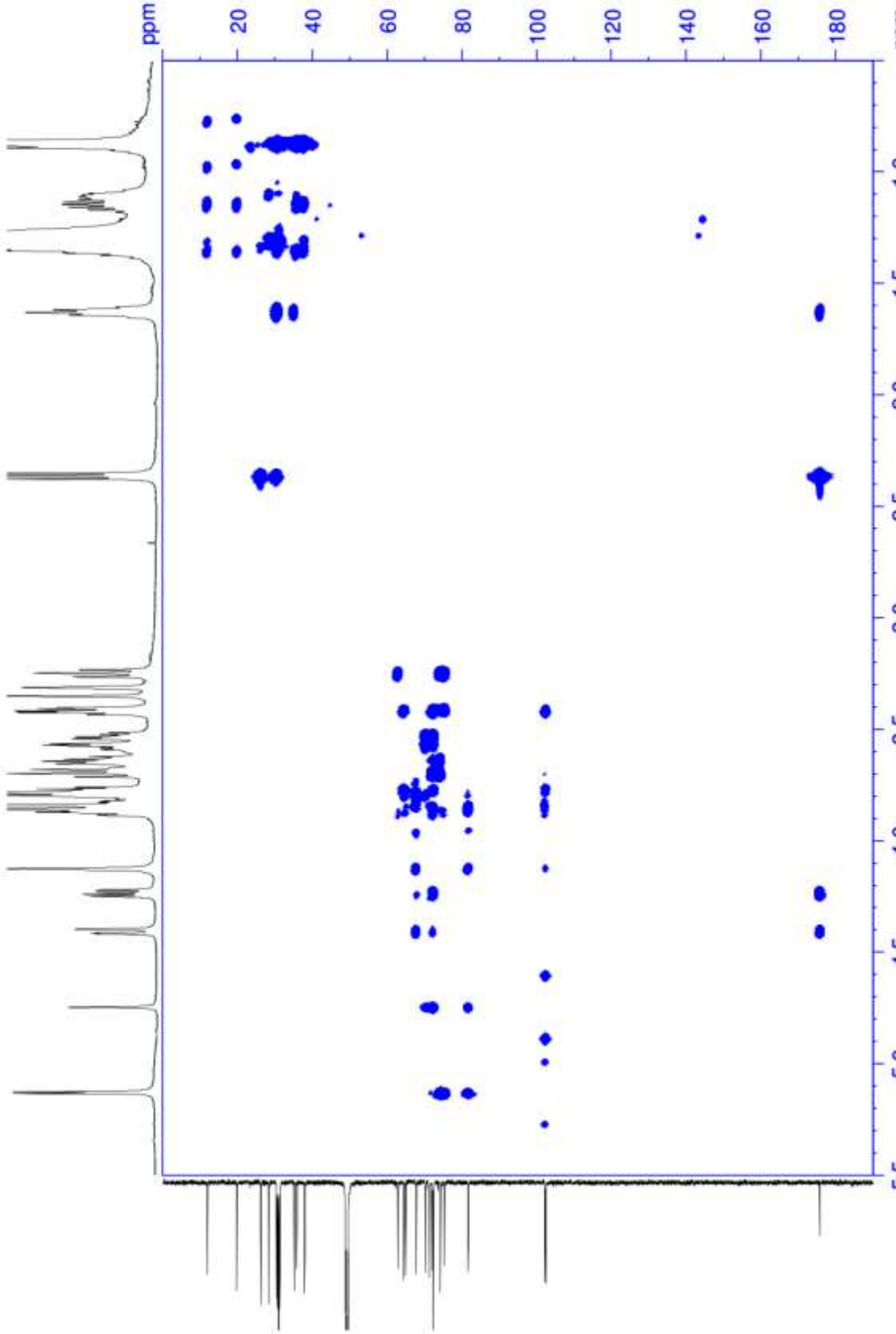

Figure S22 HRESIMS spectrum of **3**

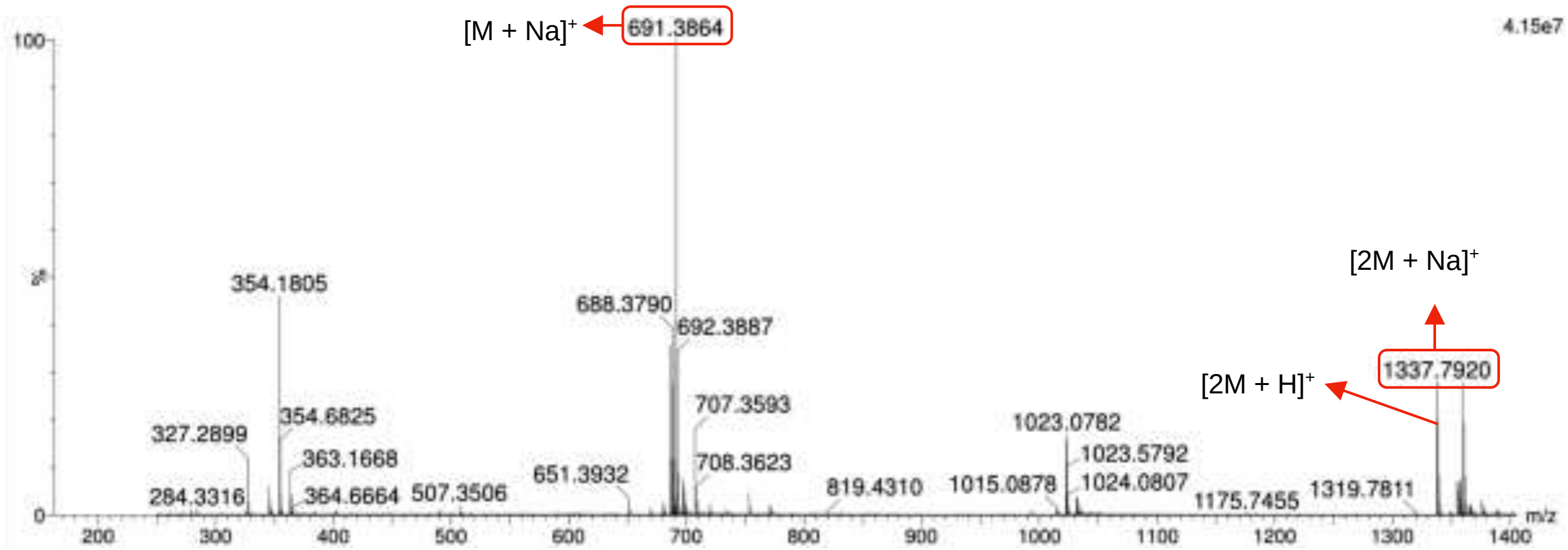

Figure S23 MS fragmentation spectrum of **3**

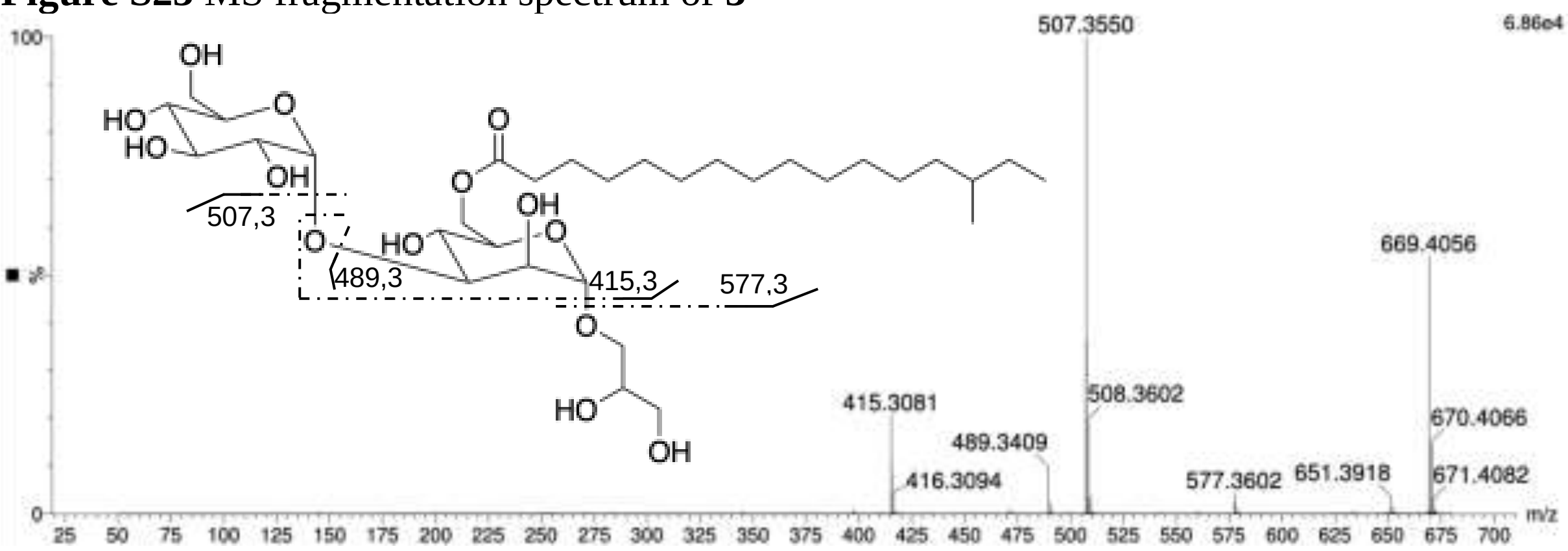

Figure S24 HRESIMS spectrum of testacoside D (**4**)

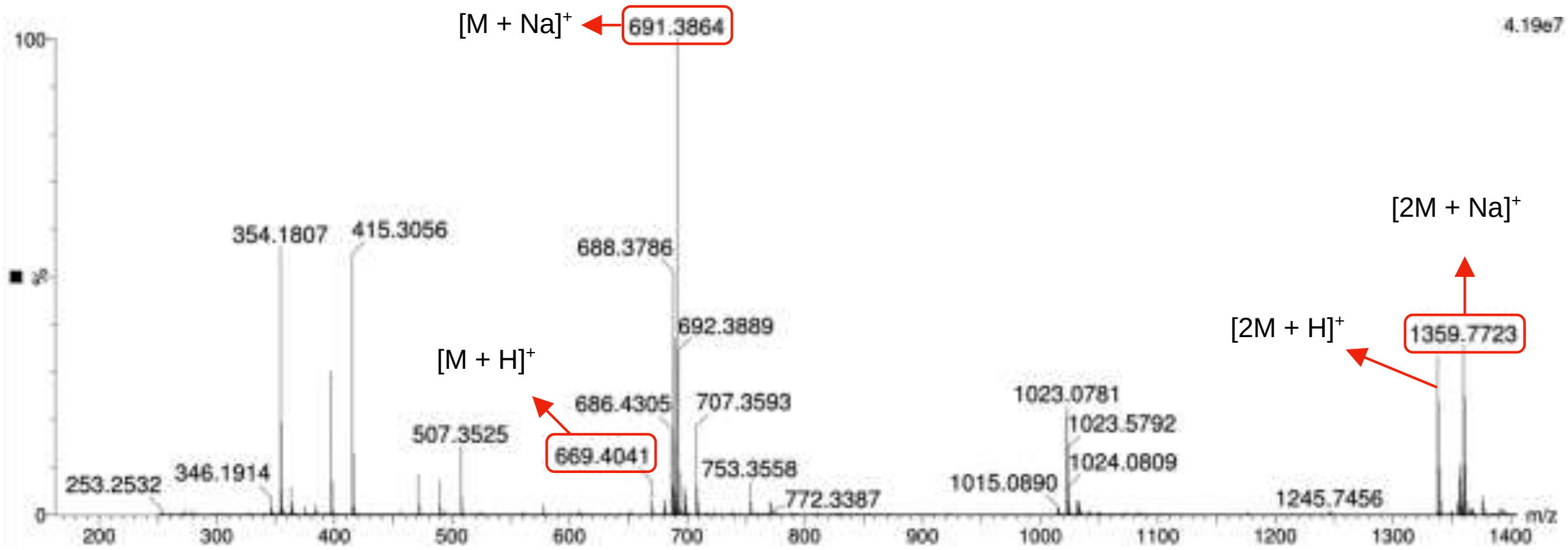

Figure S25 ¹H NMR spectrum of **4** (MeOH-d_4; 600 MHz)

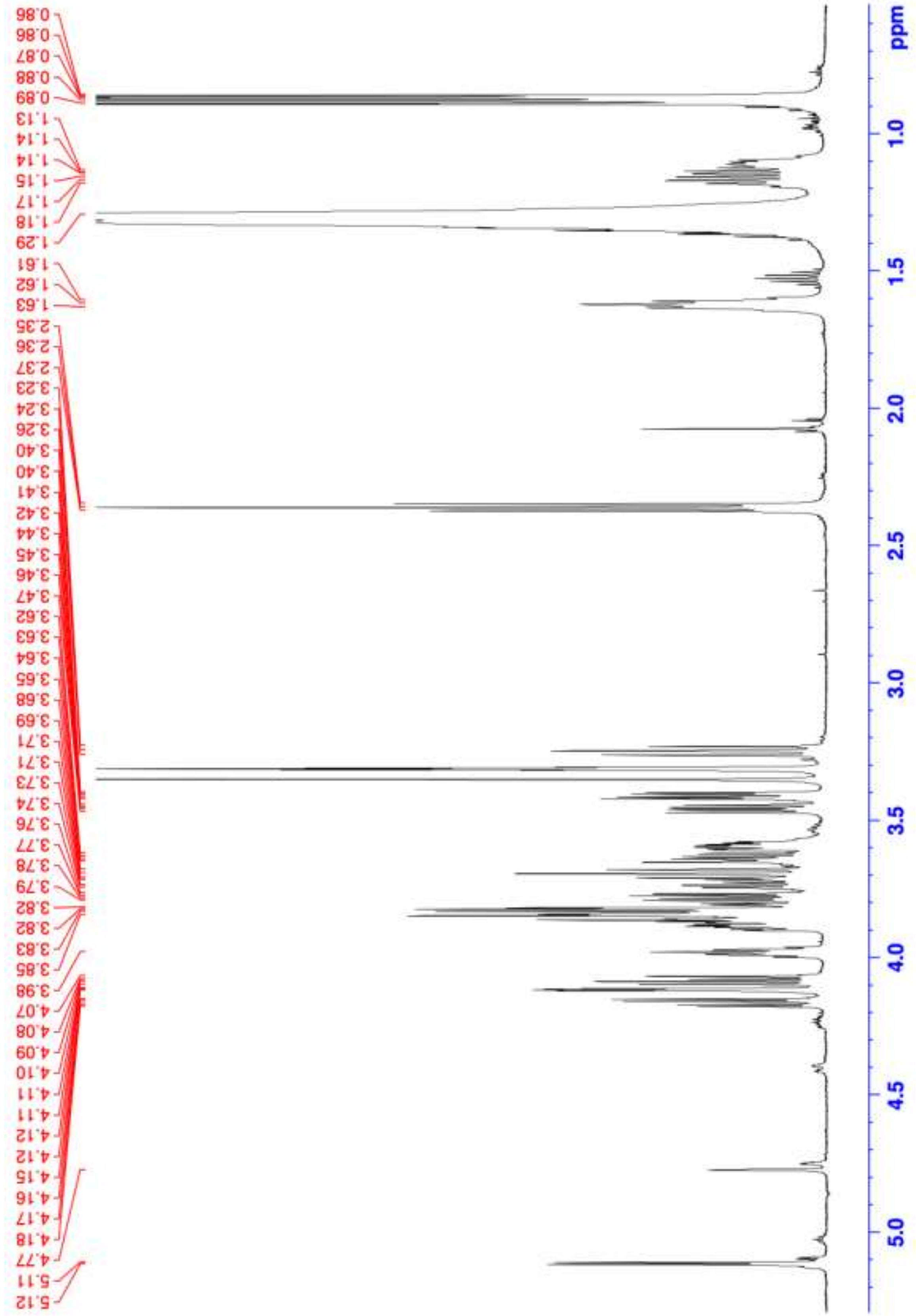

Figure S26 ^{13}C NMR spectrum of **4** (MeOH-d_4; 150 MHz)

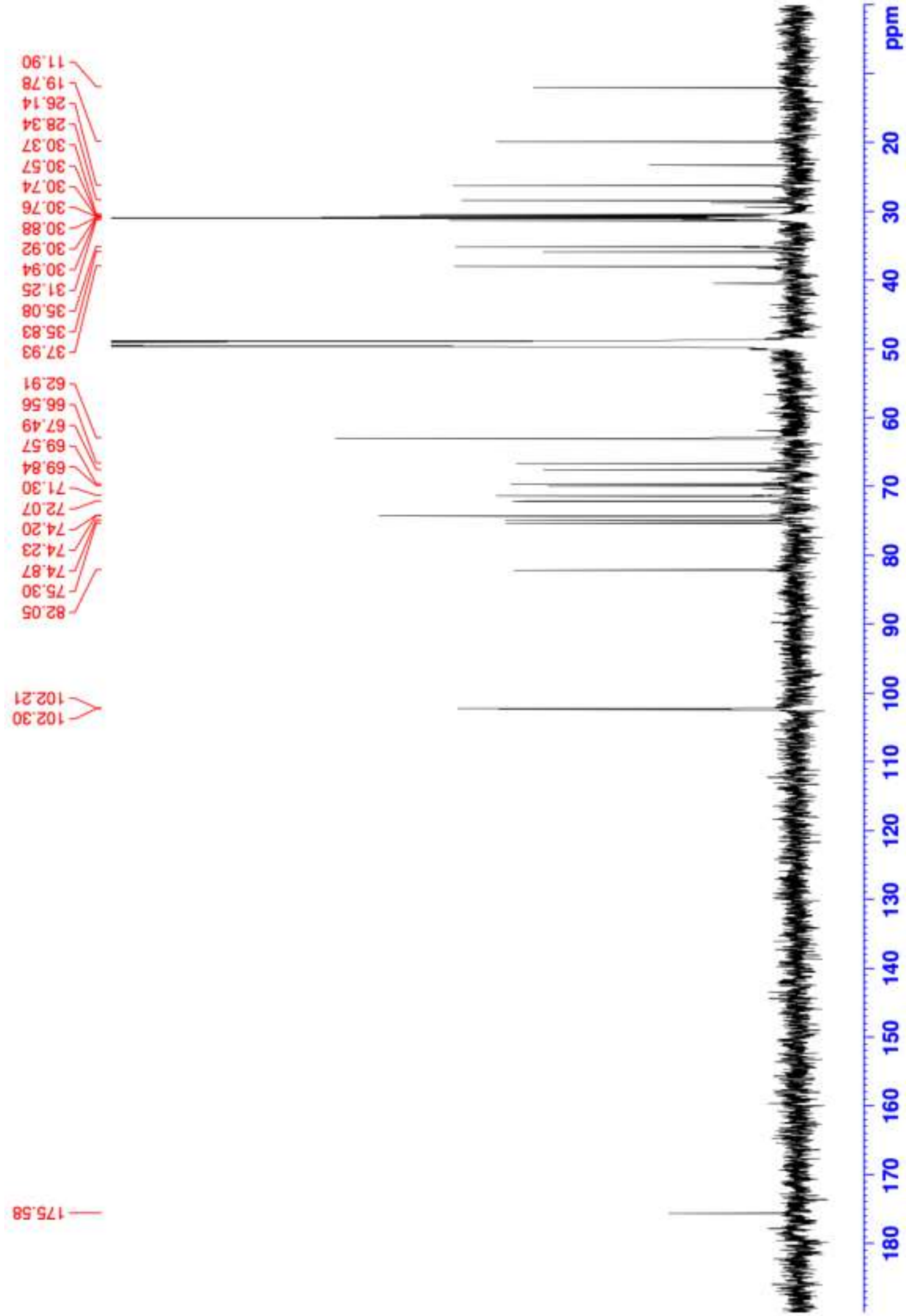

Figure S27 COSY spectrum of **4** (MeOH-d_4; 600 MHz)

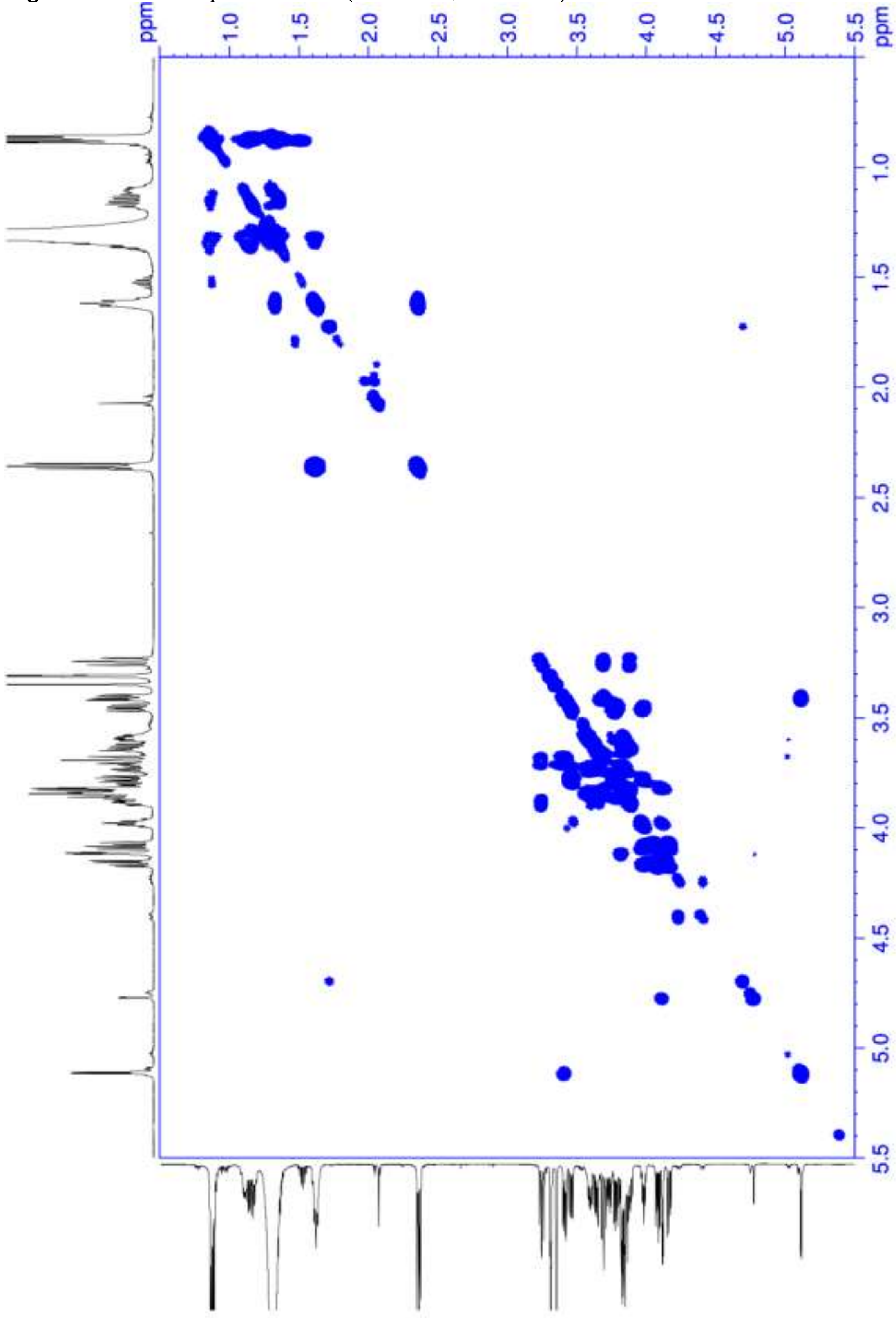

Figure S28 HSQC spectrum of **4** (MeOH-d_4; 600 MHz)

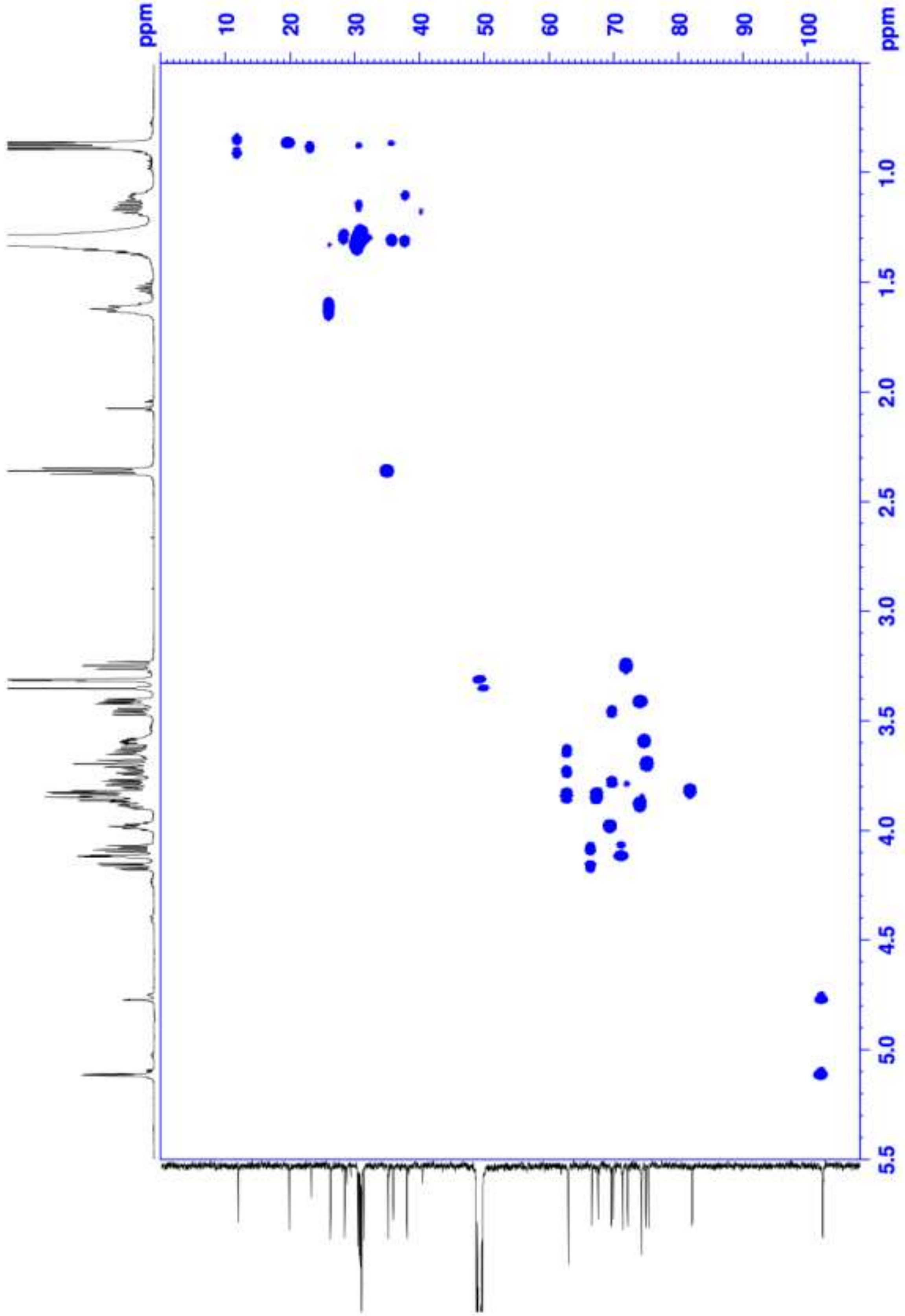

Figure S29 HMBC spectrum of **4** (MeOH-d_4; 600 MHz)

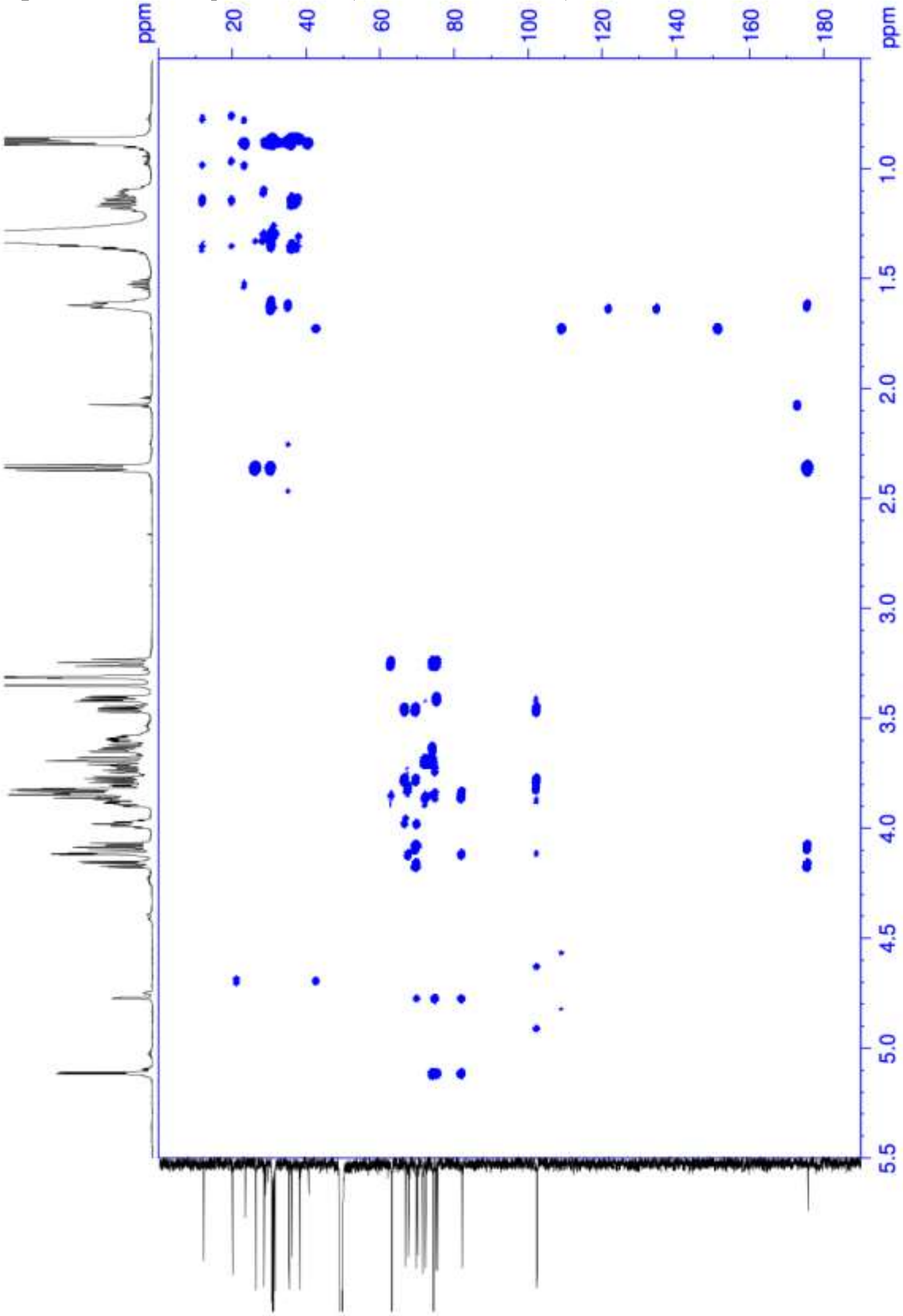

Figure S30 MS fragmentation spectrum of **4**

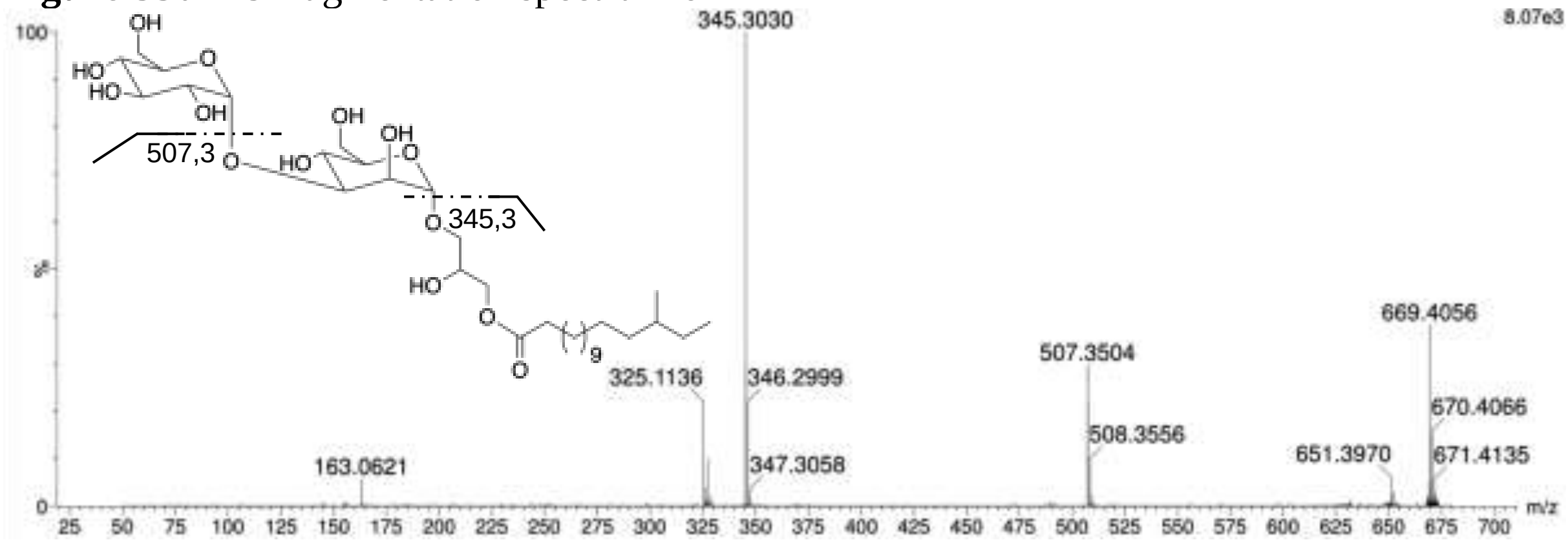

Figure S31 ¹H NMR spectrum of testacoside A peracetate (**5**) ($CDCl_3$; 600 MHz)

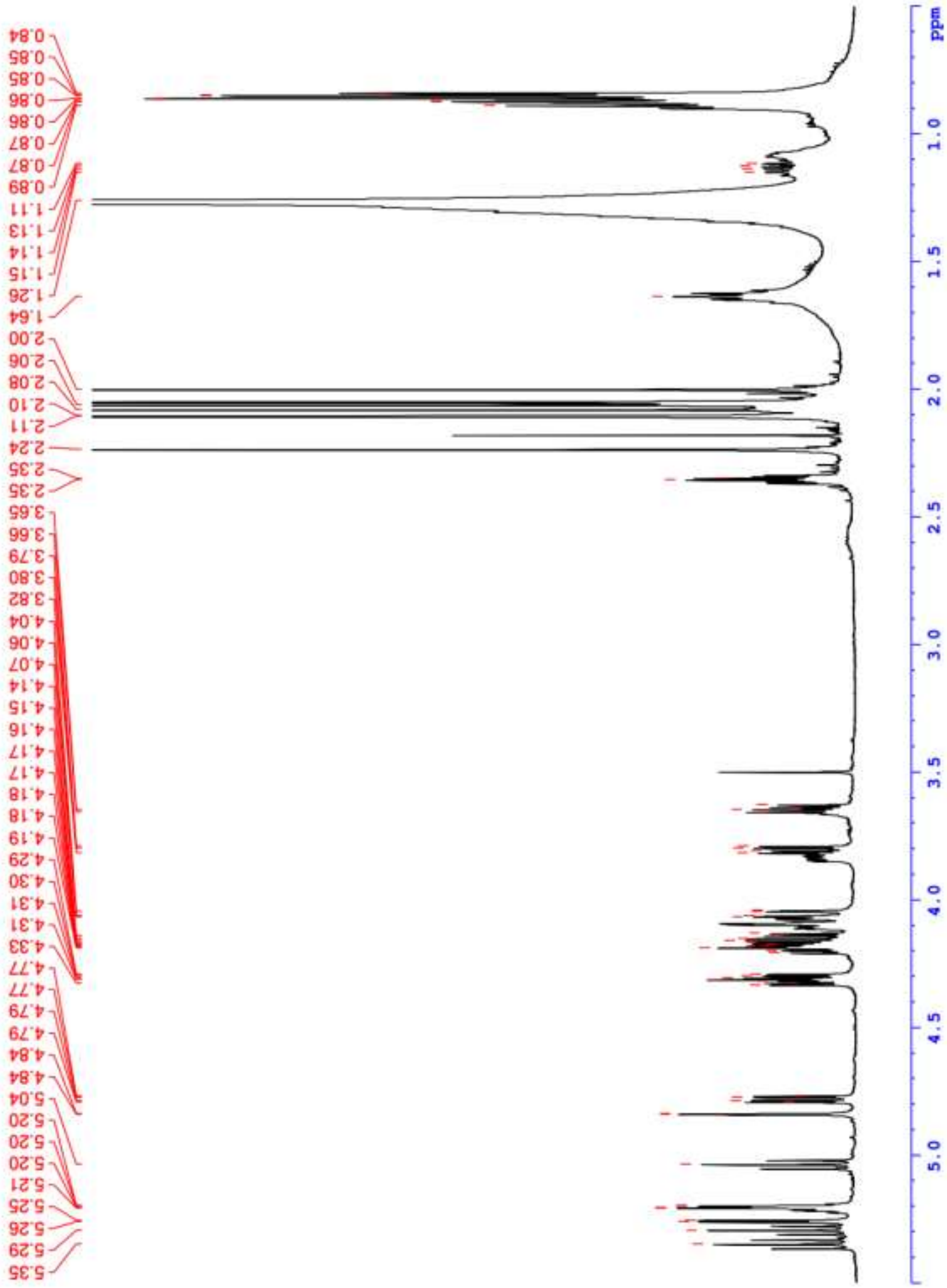

Figure S32 ^{13}C NMR spectrum of **5** ($CDCl_3$; 150 MHz)

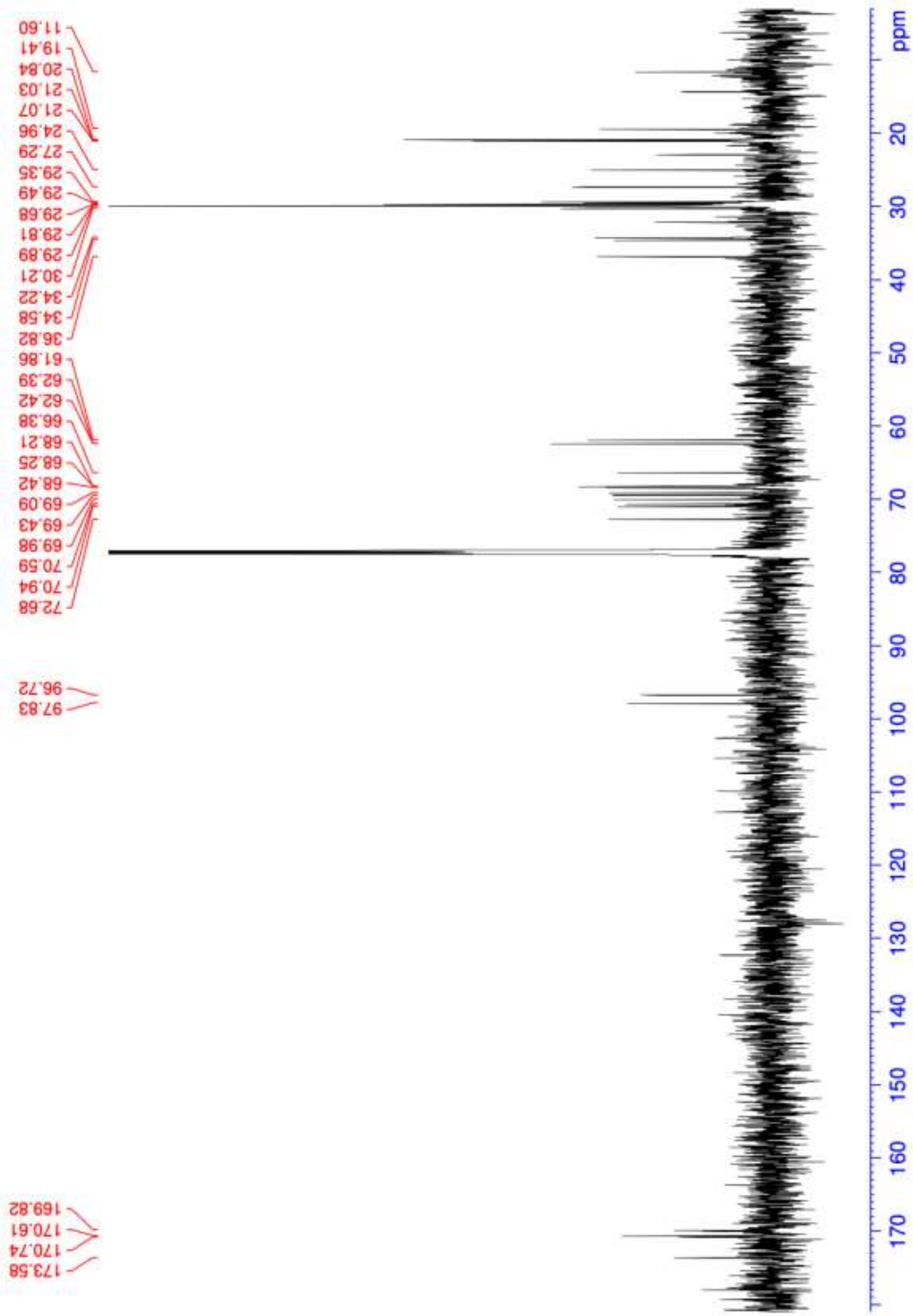

Figure S33 DEPT-135 spectrum of **5** ($CDCl_3$; 150 MHz)

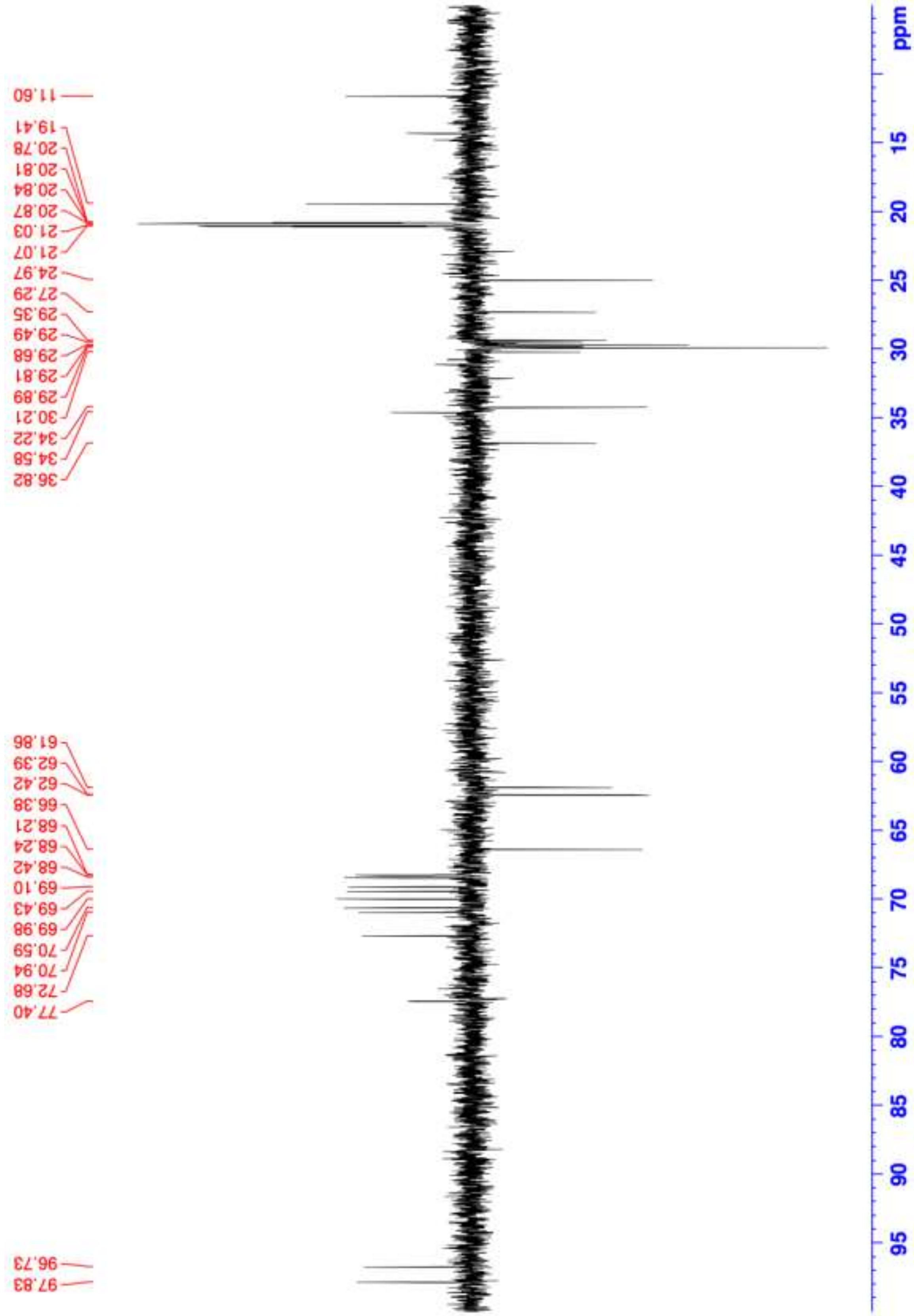

Figure S34 COSY spectrum of **5** ($CDCl_3$; 600 MHz)

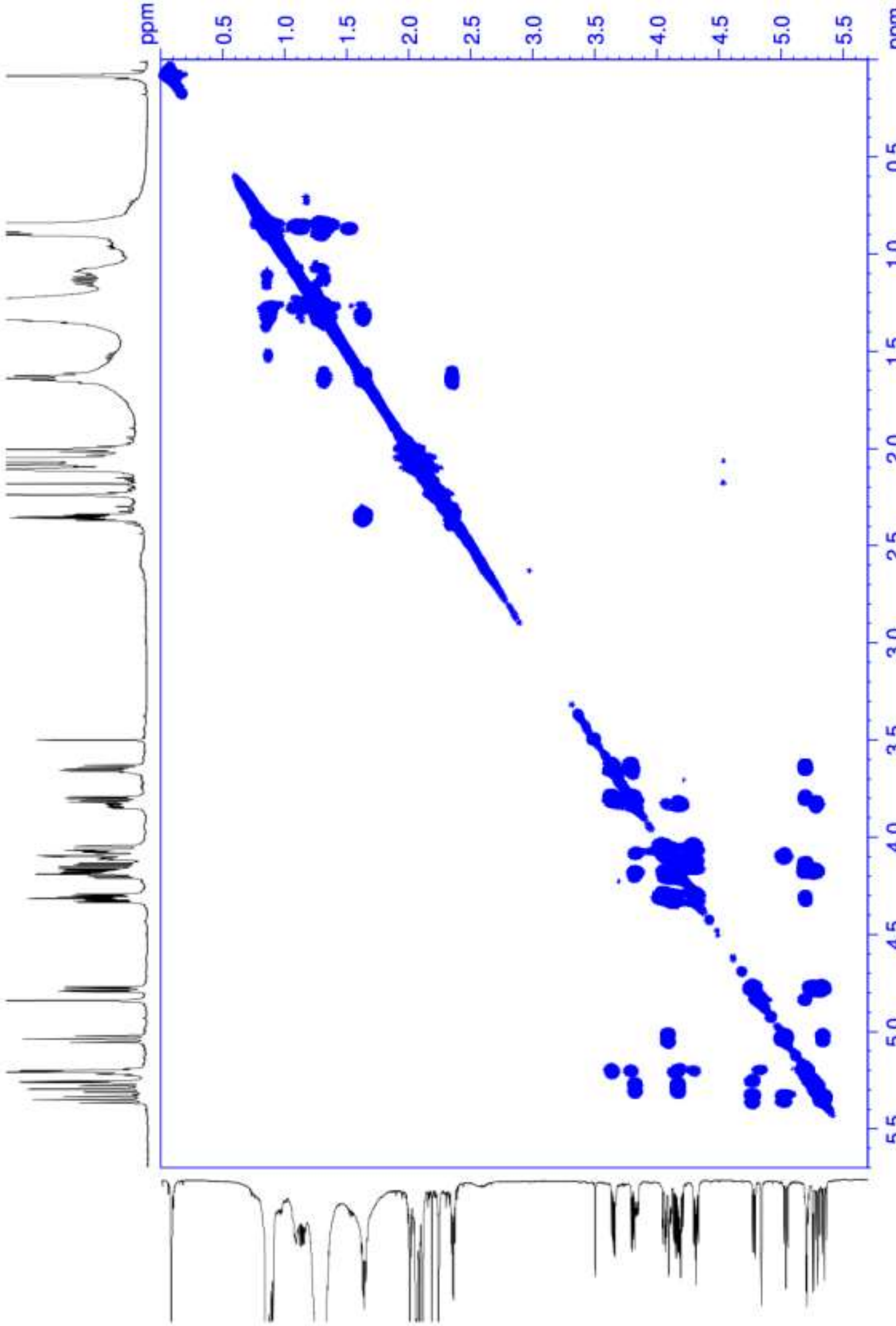

Figure S35 HSQC spectrum of **5** ($CDCl_3$; 600 MHz)

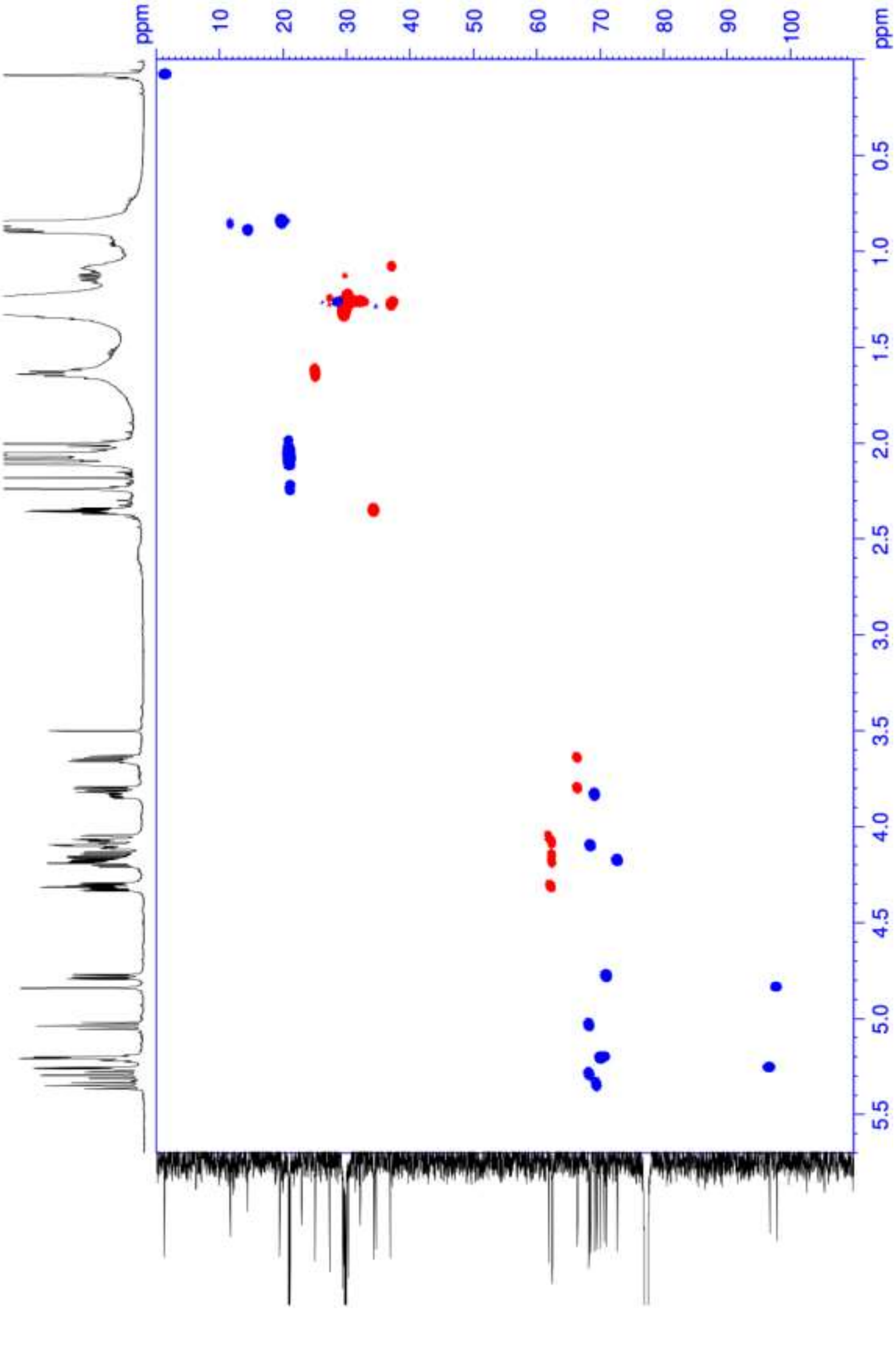

Figure S36 HMBC spectrum of **5** ($CDCl_3$; 600 MHz)

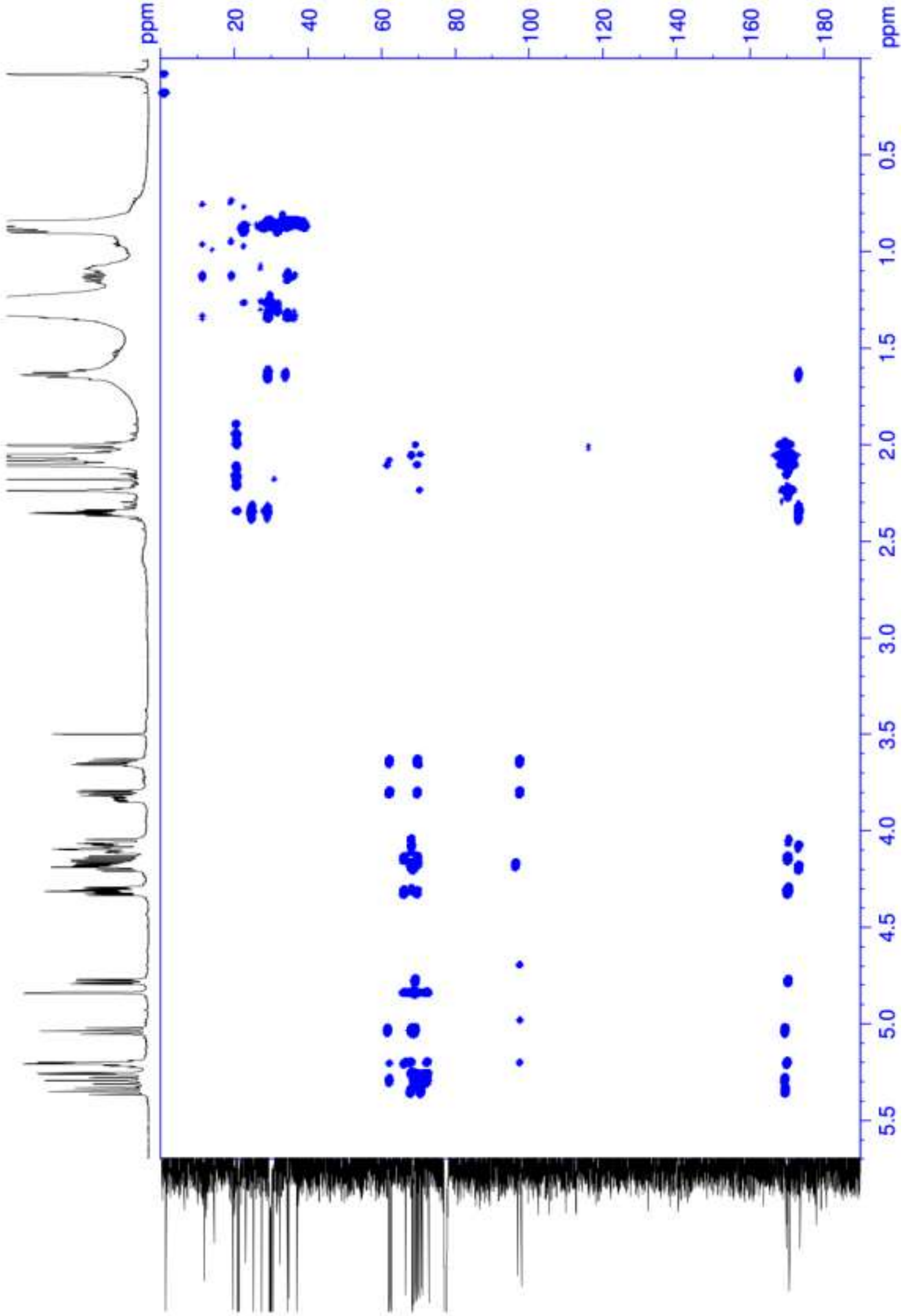

Figure S37 HRESIMS spectrum of **5**

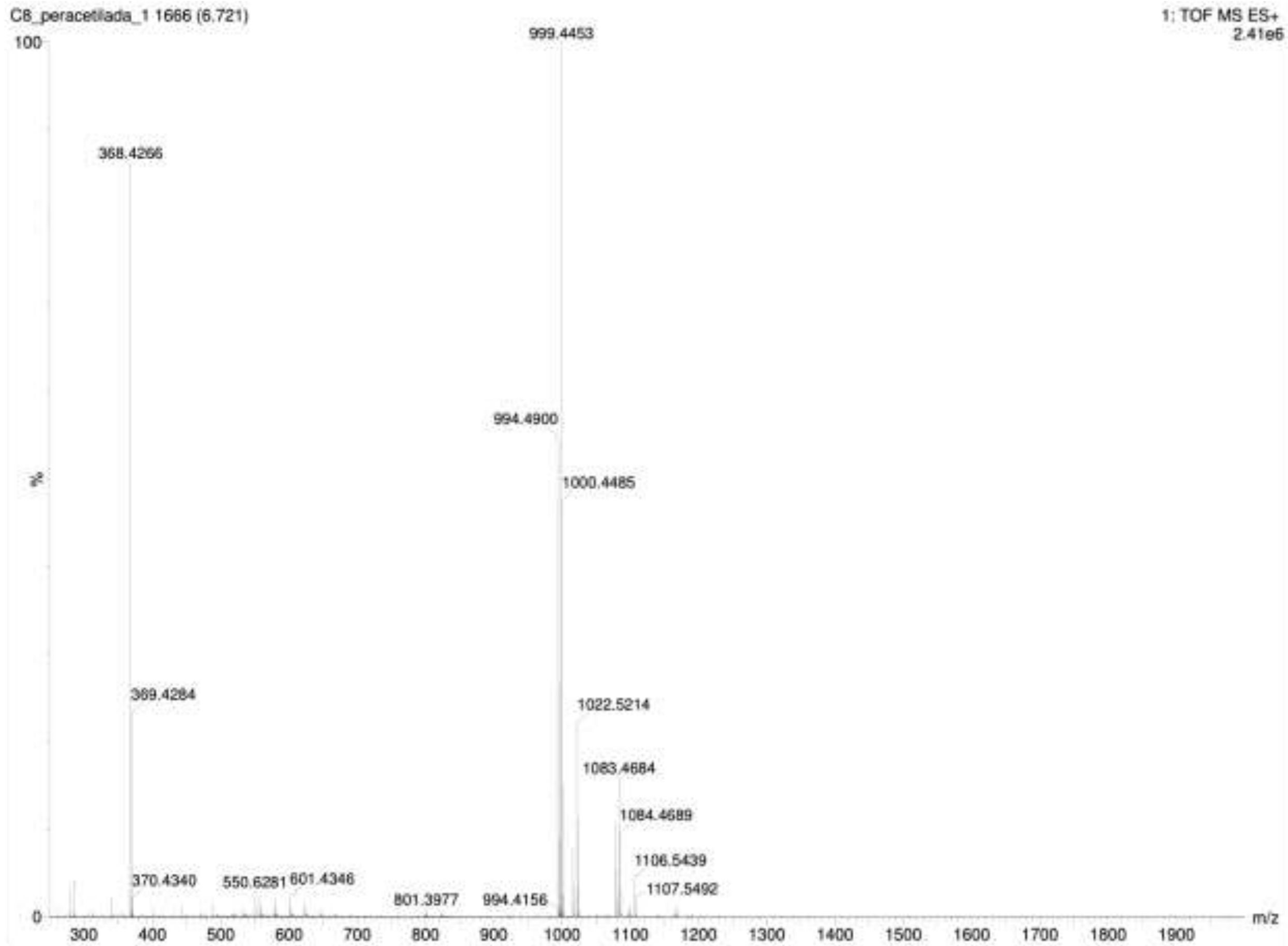

Figure S38 ^{1}H NMR spectrum of testacoside B peracetate (**6**) ($CDCl_3$; 600 MHz)

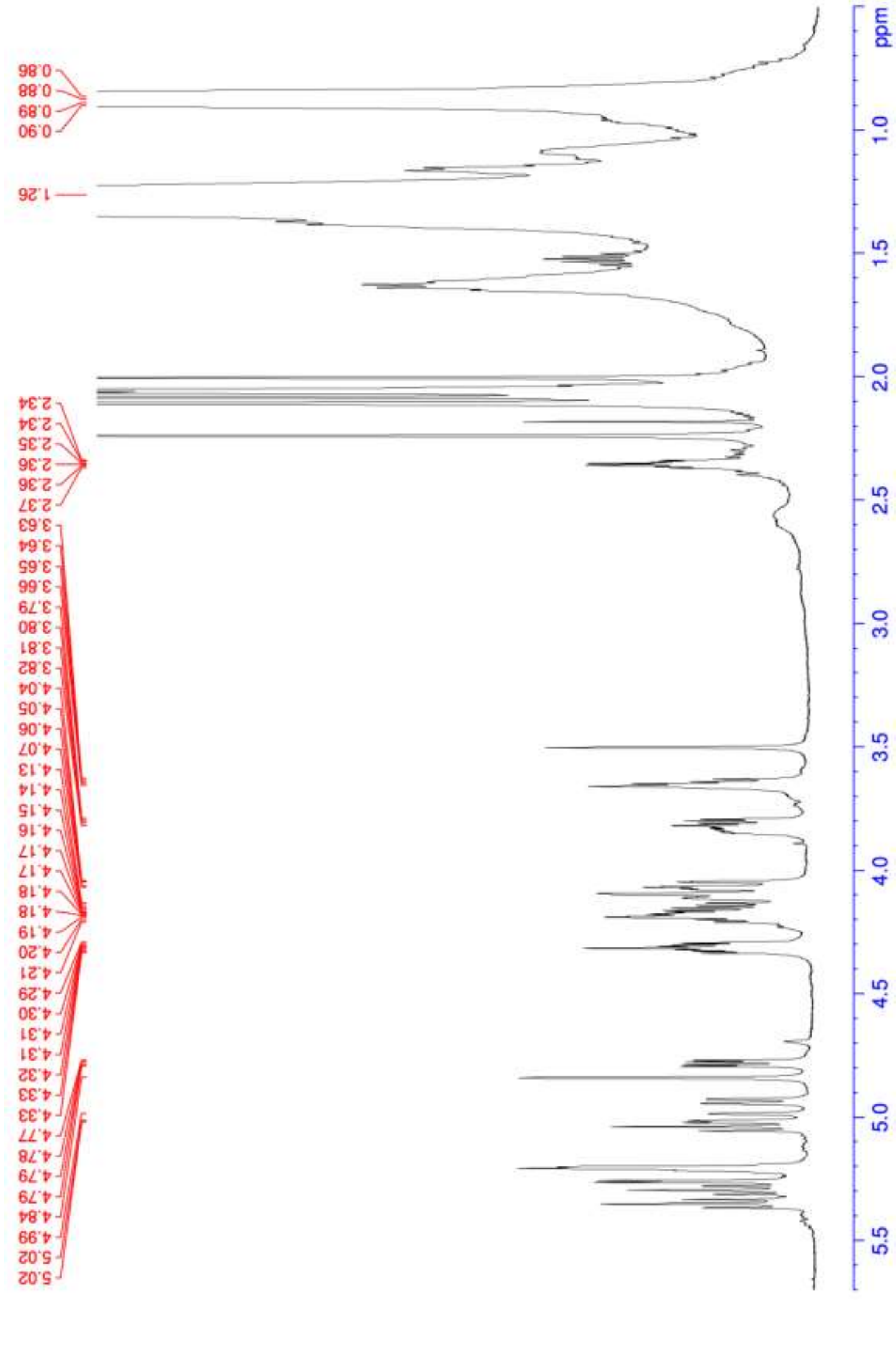

Figure S39 ^{13}C NMR spectrum of **6** ($CDCl_3$; 150 MHz)

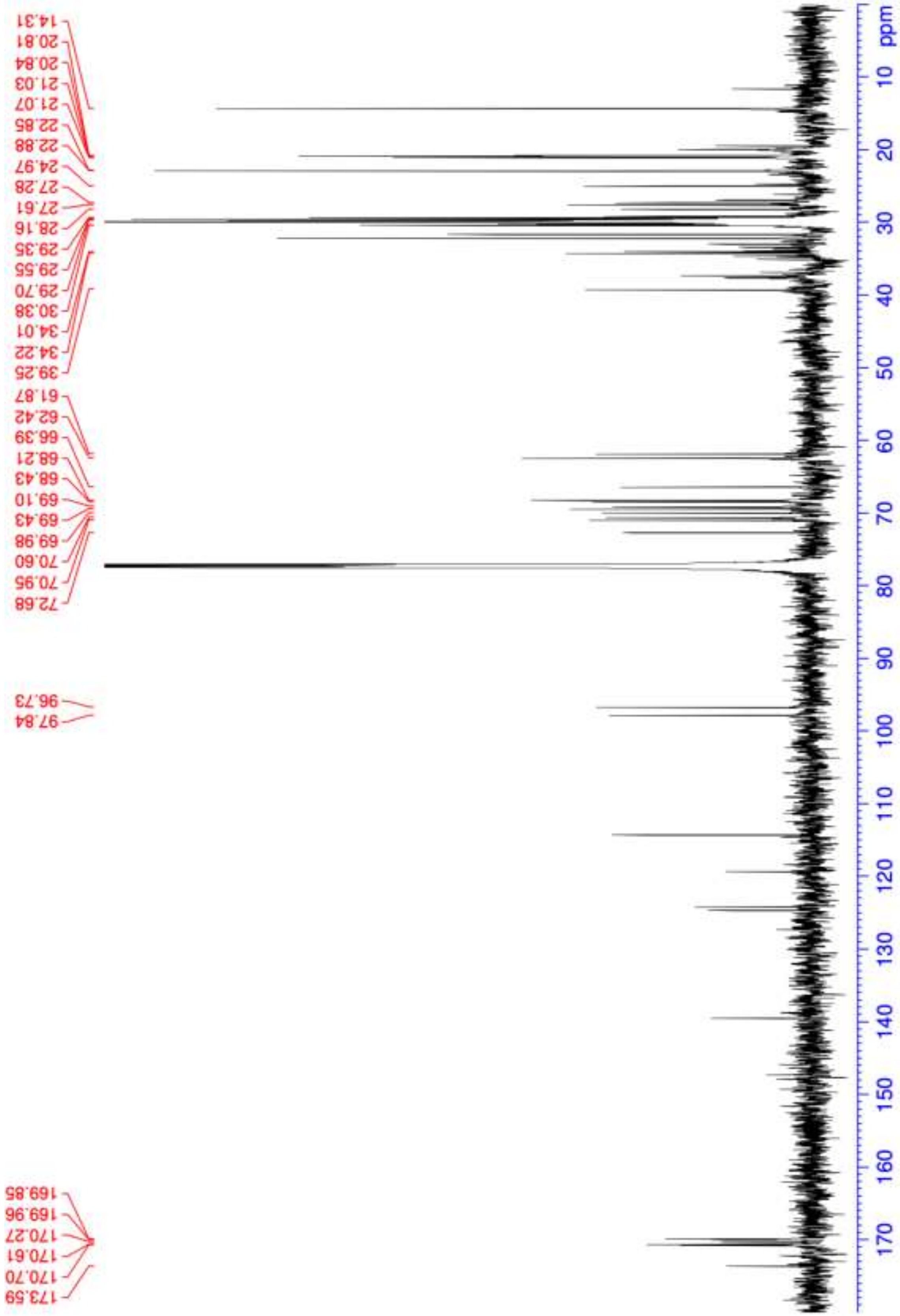

Figure S40 DEPT-135 spectrum of **6** ($CDCl_3$; 150 MHz)

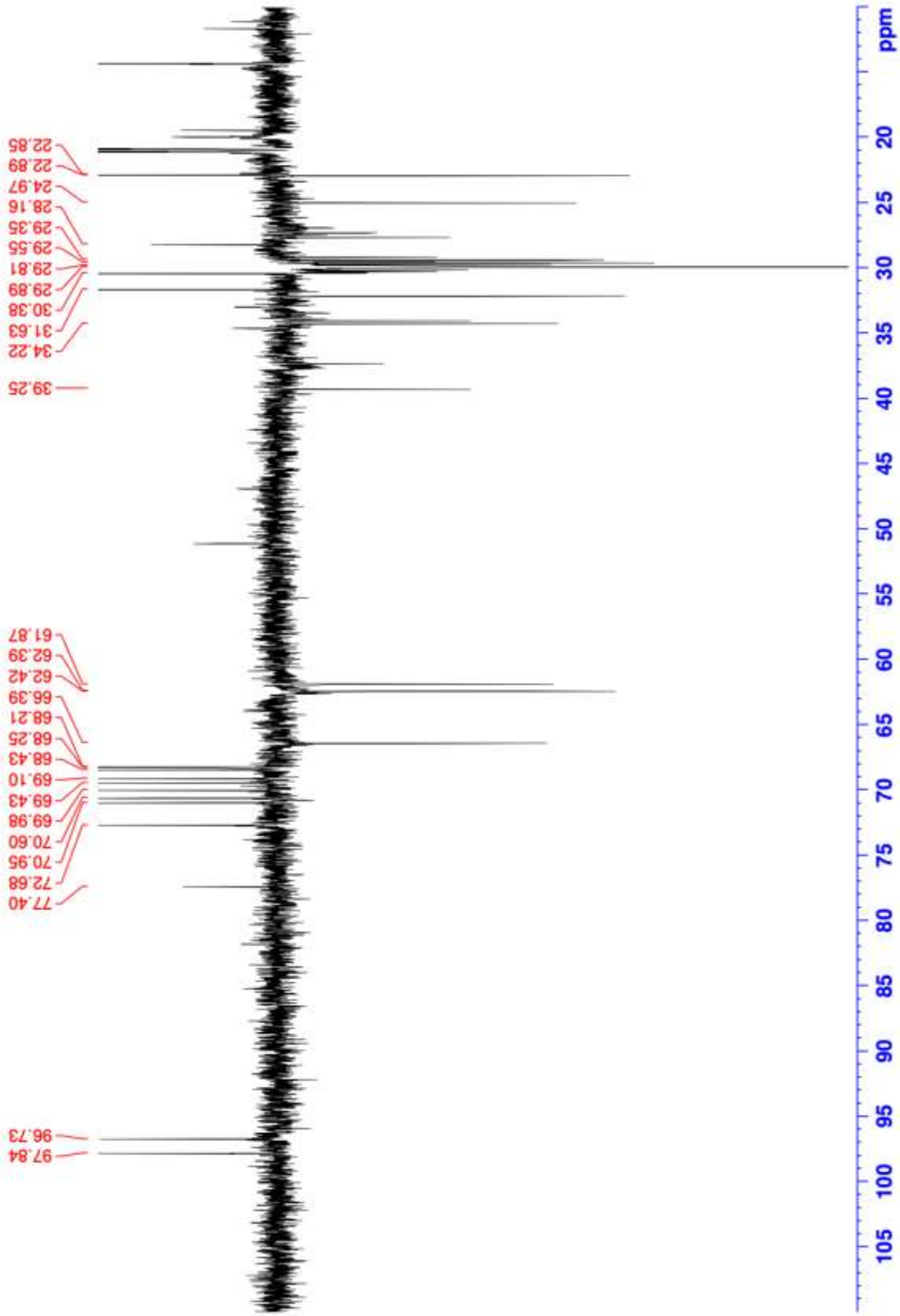

Figure S41 COSY spectrum of **6** ($CDCl_3$; 600 MHz)

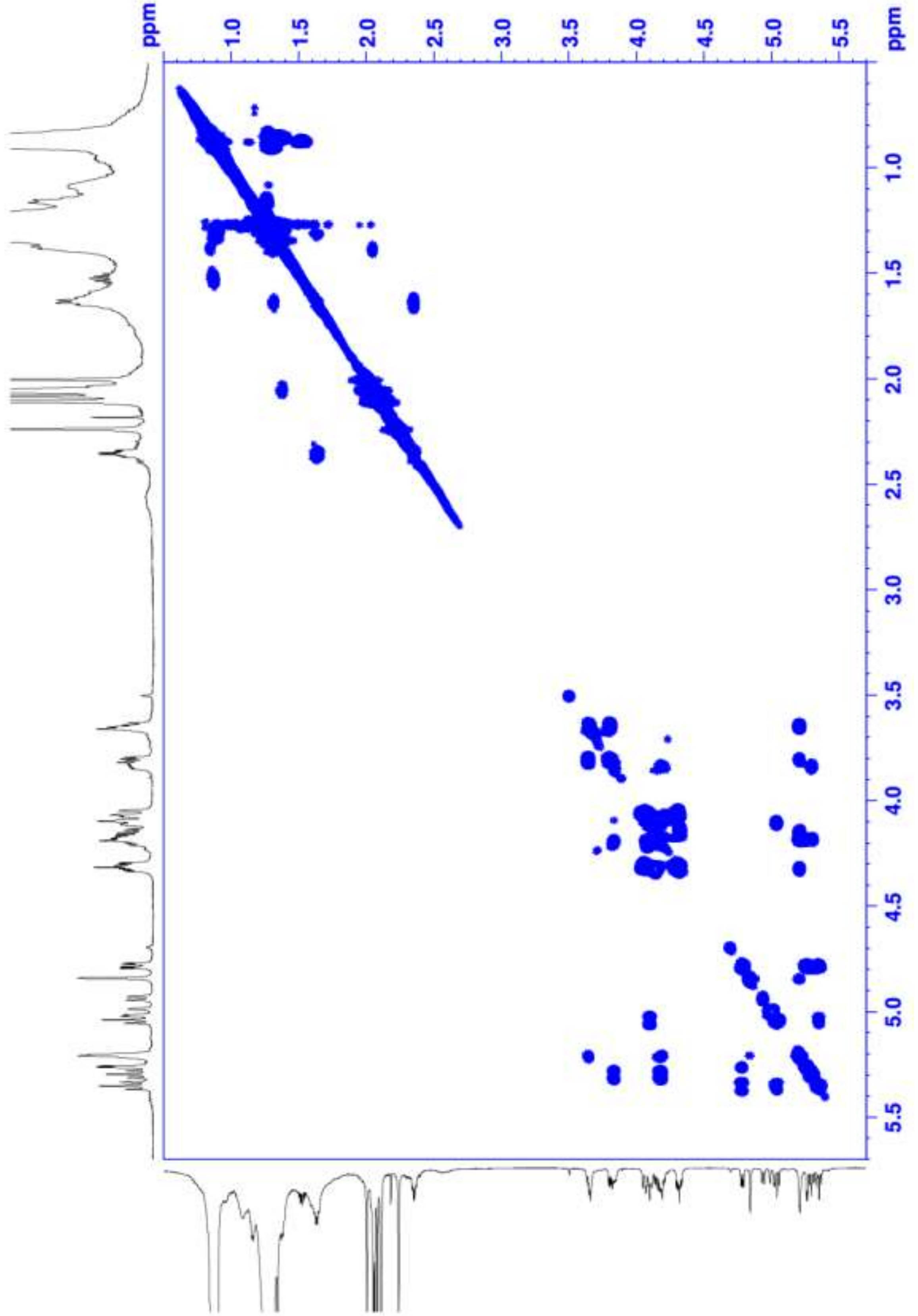

Figure S42 HSQC spectrum of **6** ($CDCl_3$; 600 MHz)

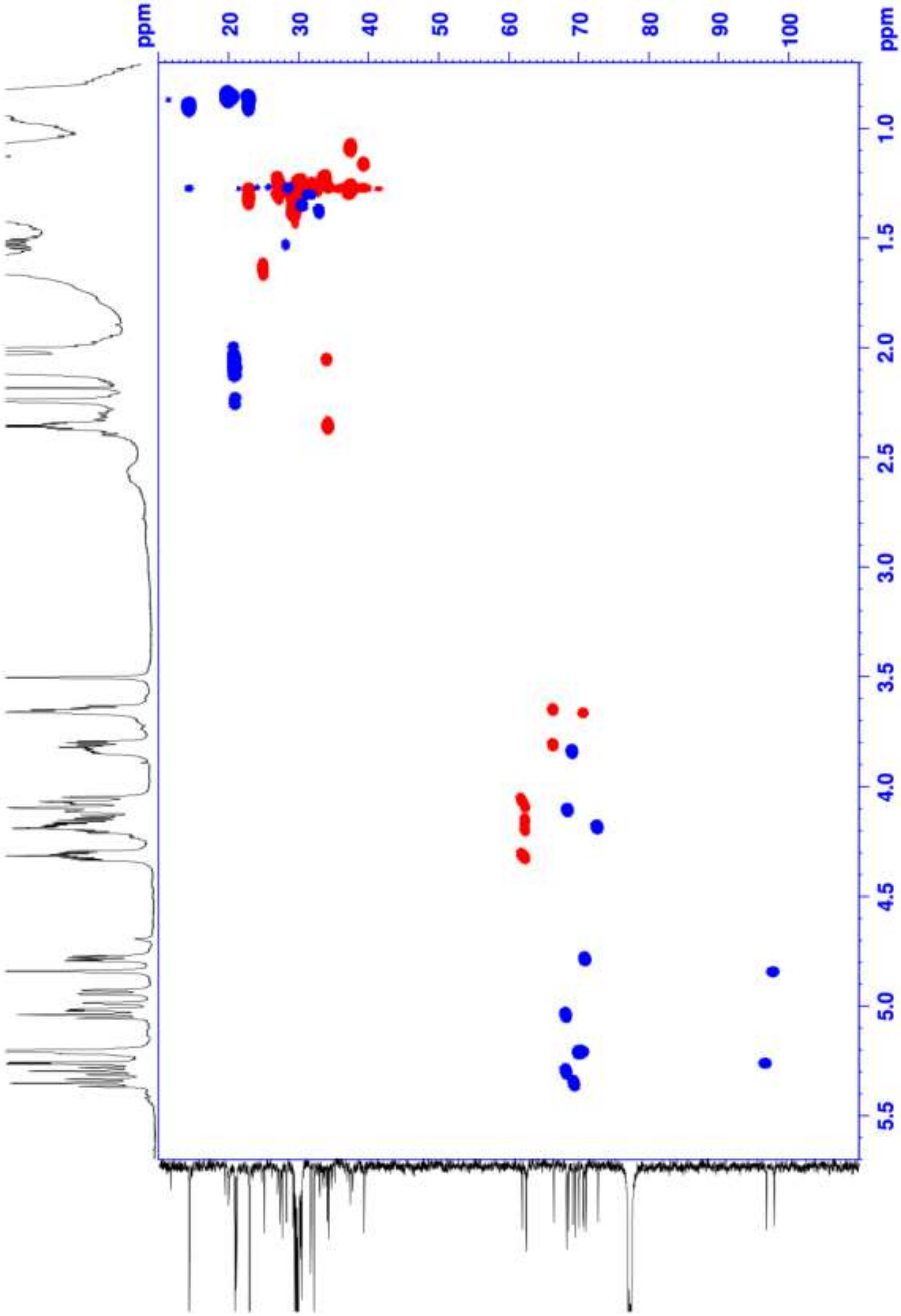

Figure S43 HMBC spectrum of **6** ($CDCl_3$; 600 MHz)

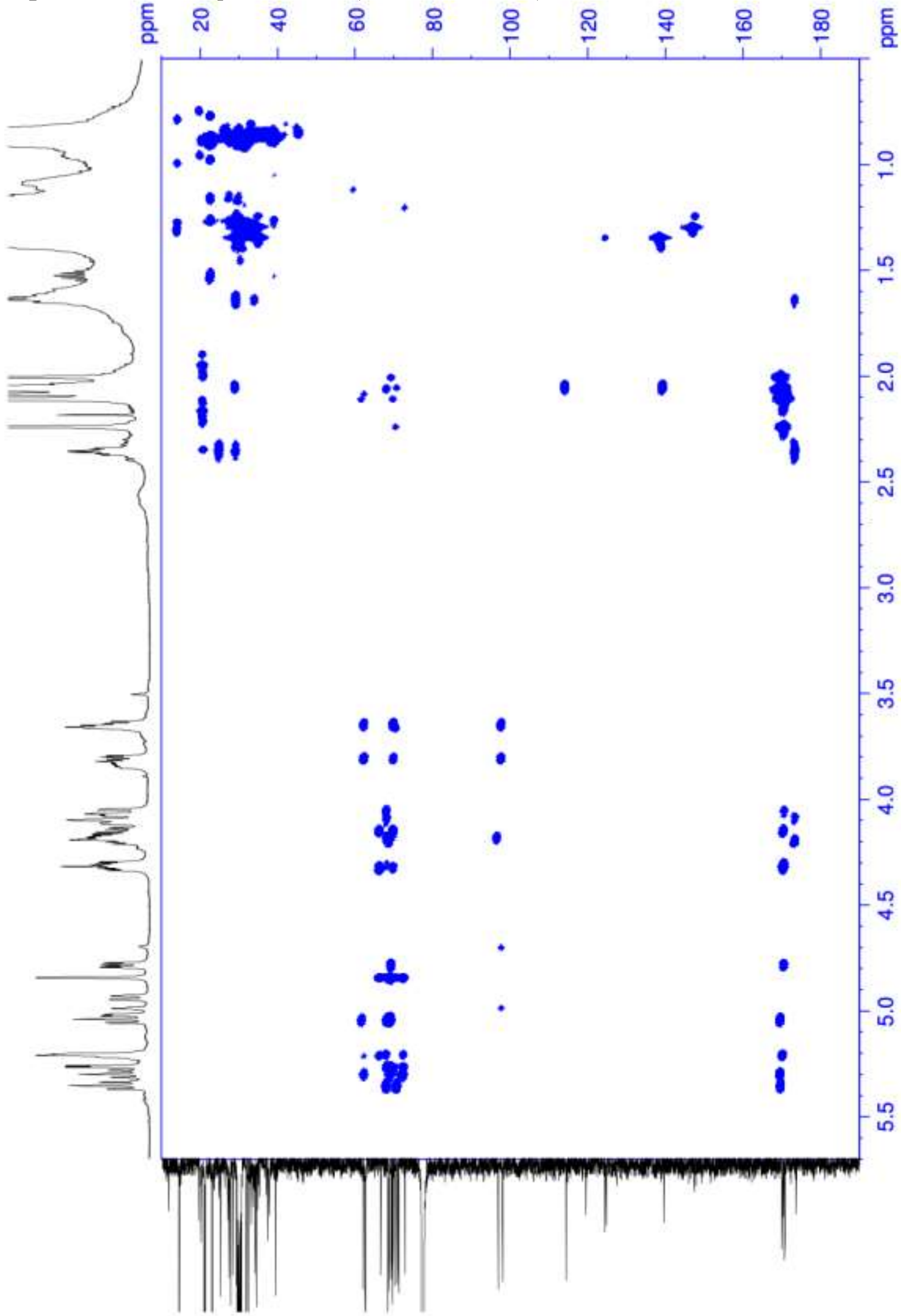

Figure S44 ^{1}H NMR spectrum of testacoside C peracetate (**7**) ($CDCl_3$; 600 MHz)

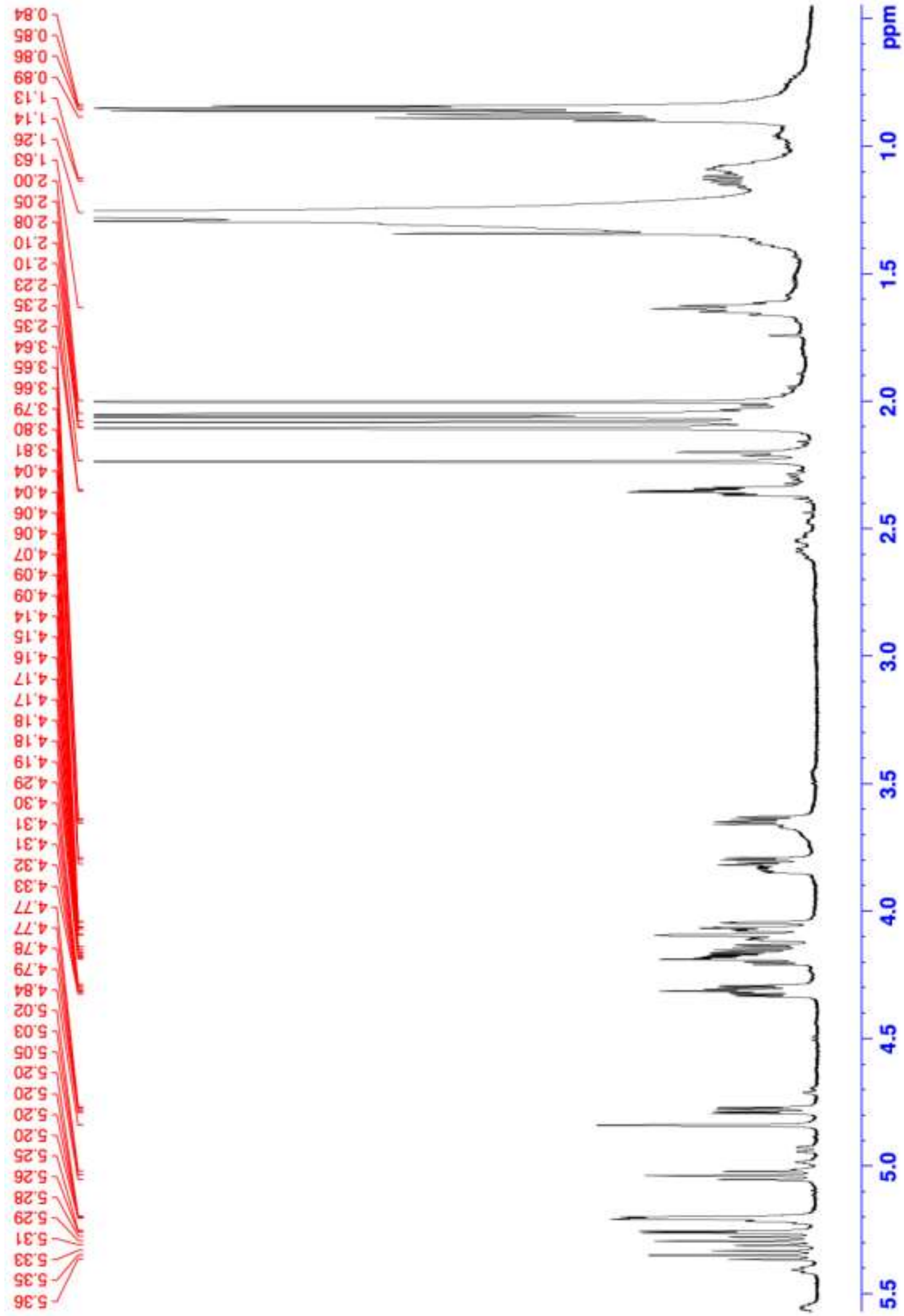

Figure S45 ^{13}C NMR spectrum of **7** ($CDCl_3$; 150 MHz)

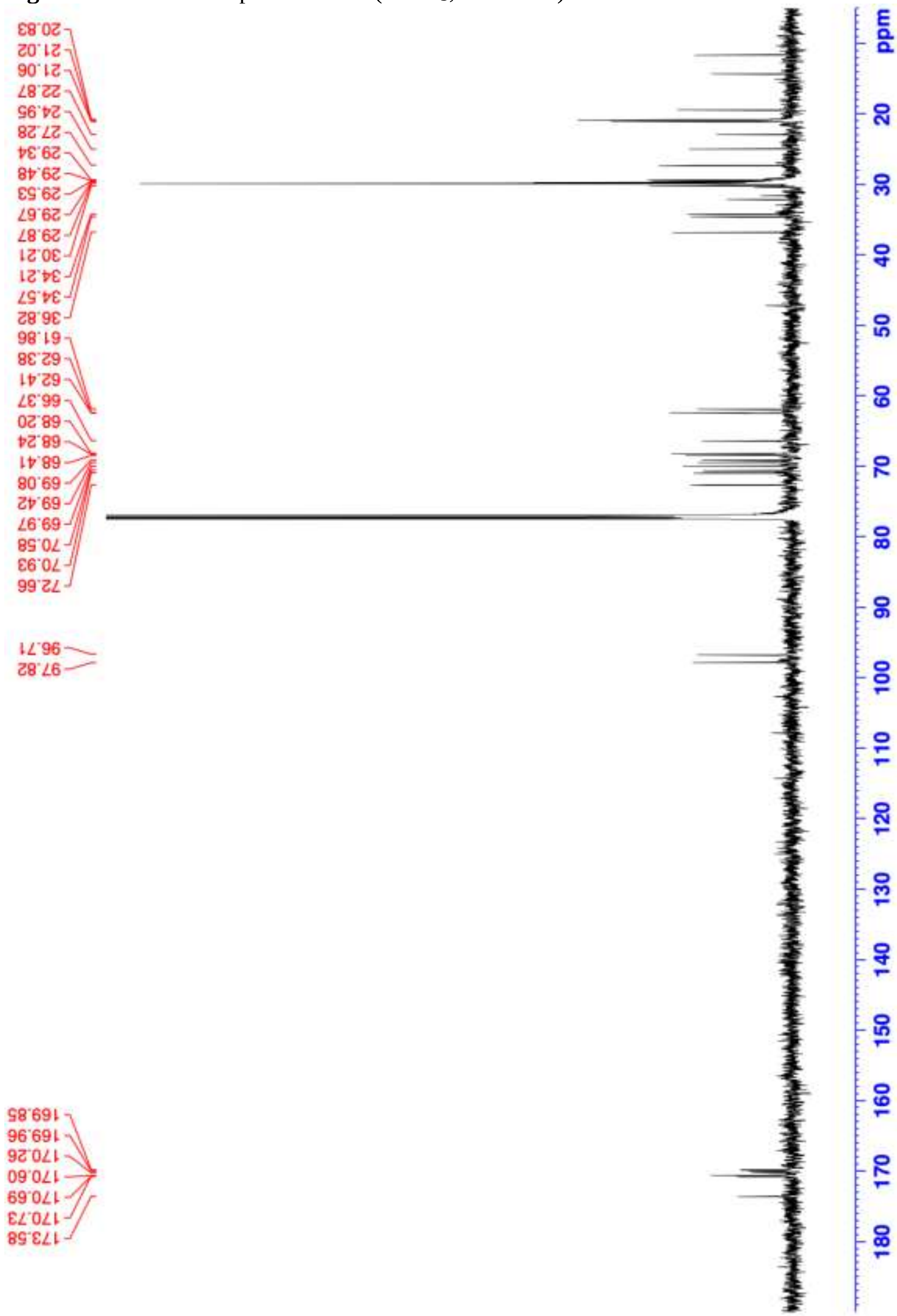

Figure S46 DEPT-135 spectrum of **7** ($CDCl_3$; 150 MHz)

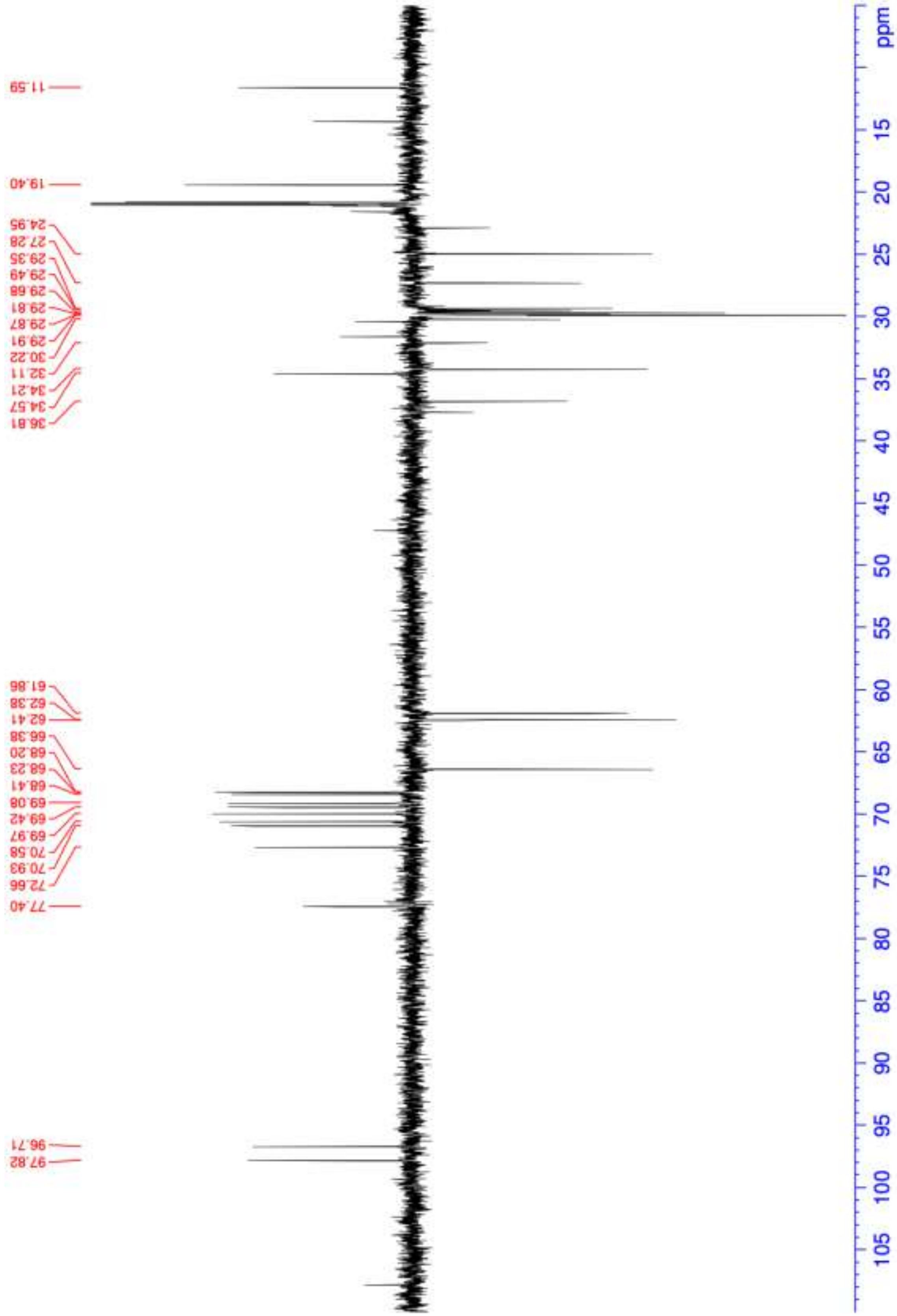

Figure S47 COSY spectrum of **7** ($CDCl_3$; 600 MHz)

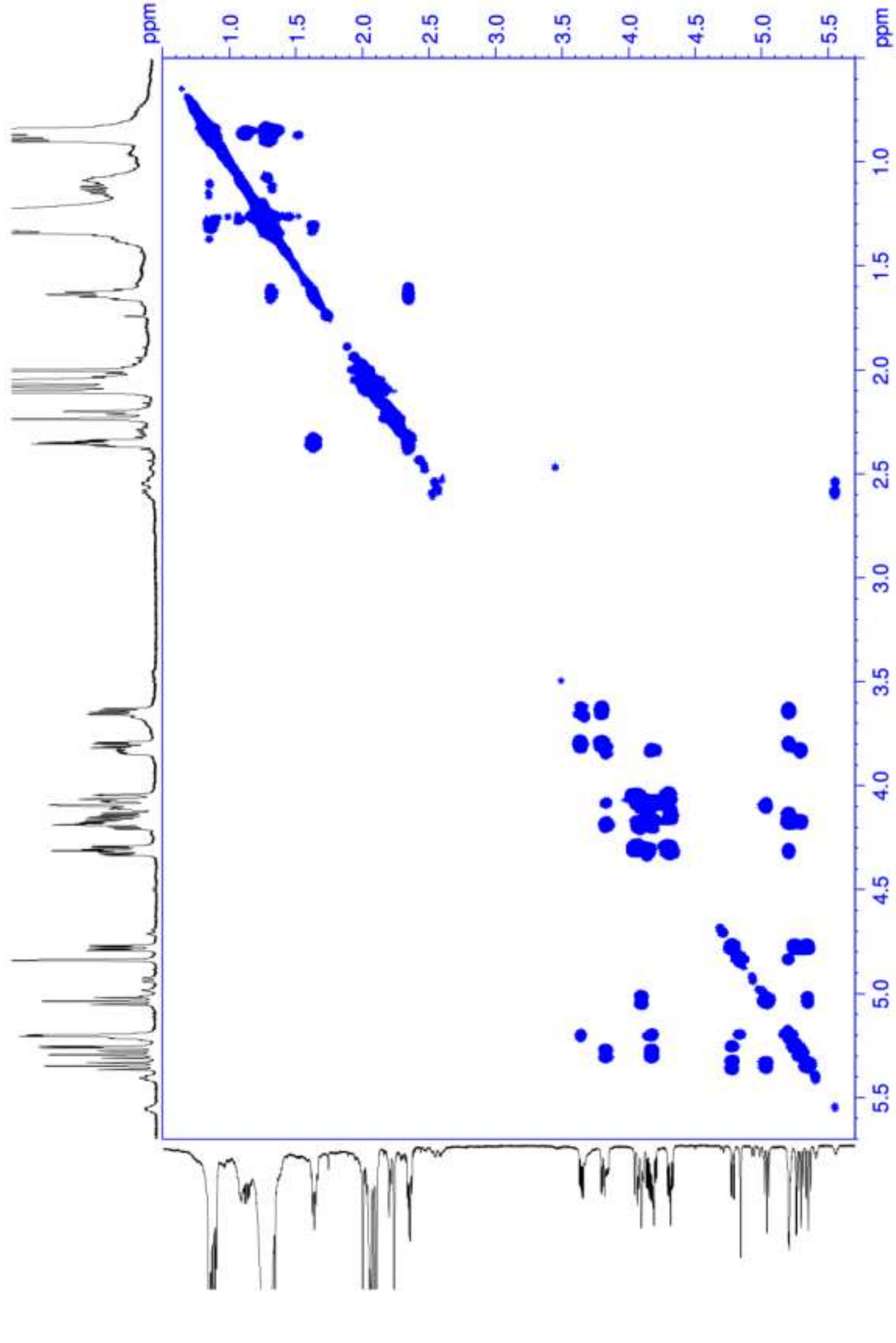

Figure S48 HSQC spectrum of **7** ($CDCl_3$; 600 MHz)

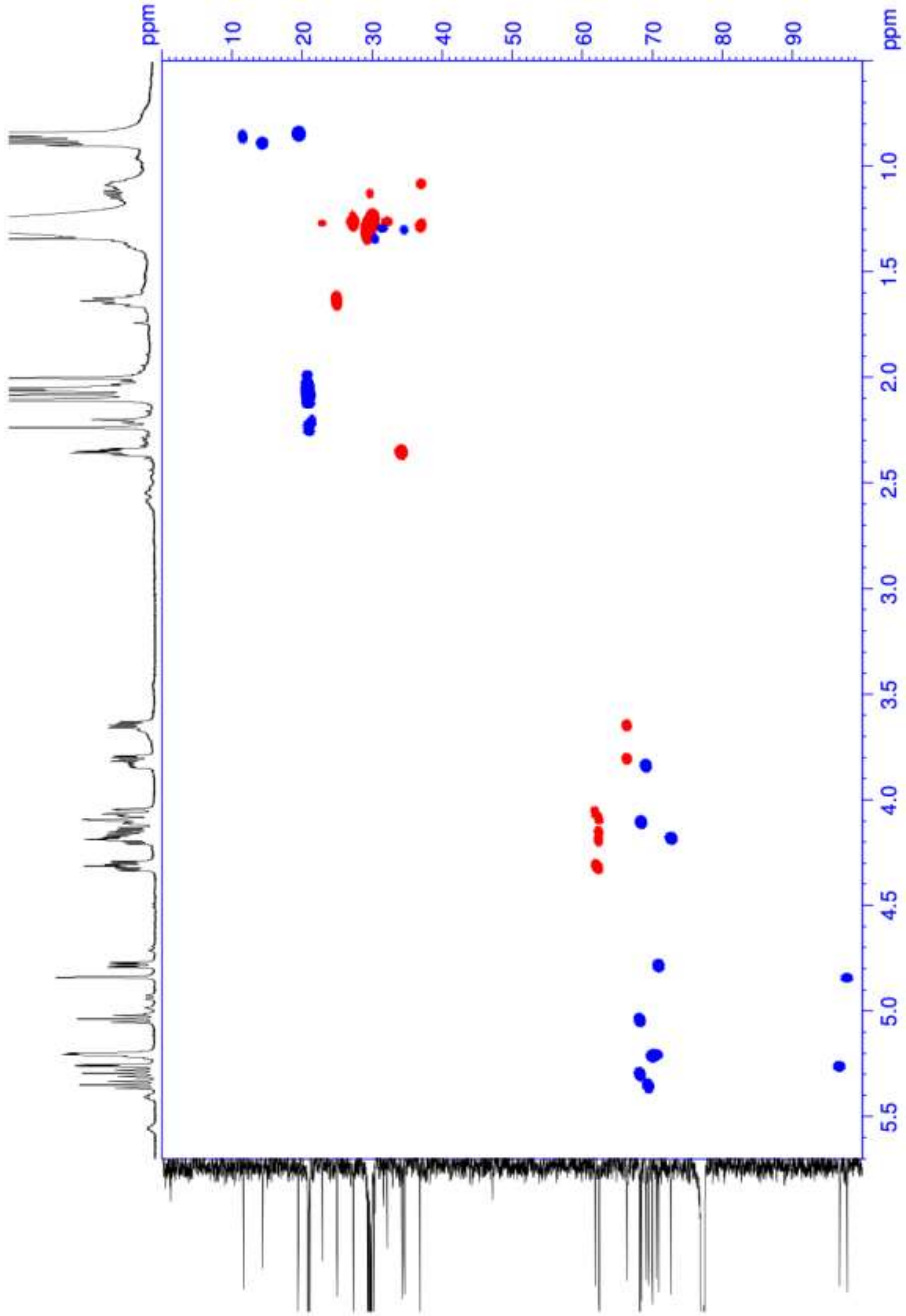

Figure S49 HMBC spectrum of **7** ($CDCl_3$; 600 MHz)

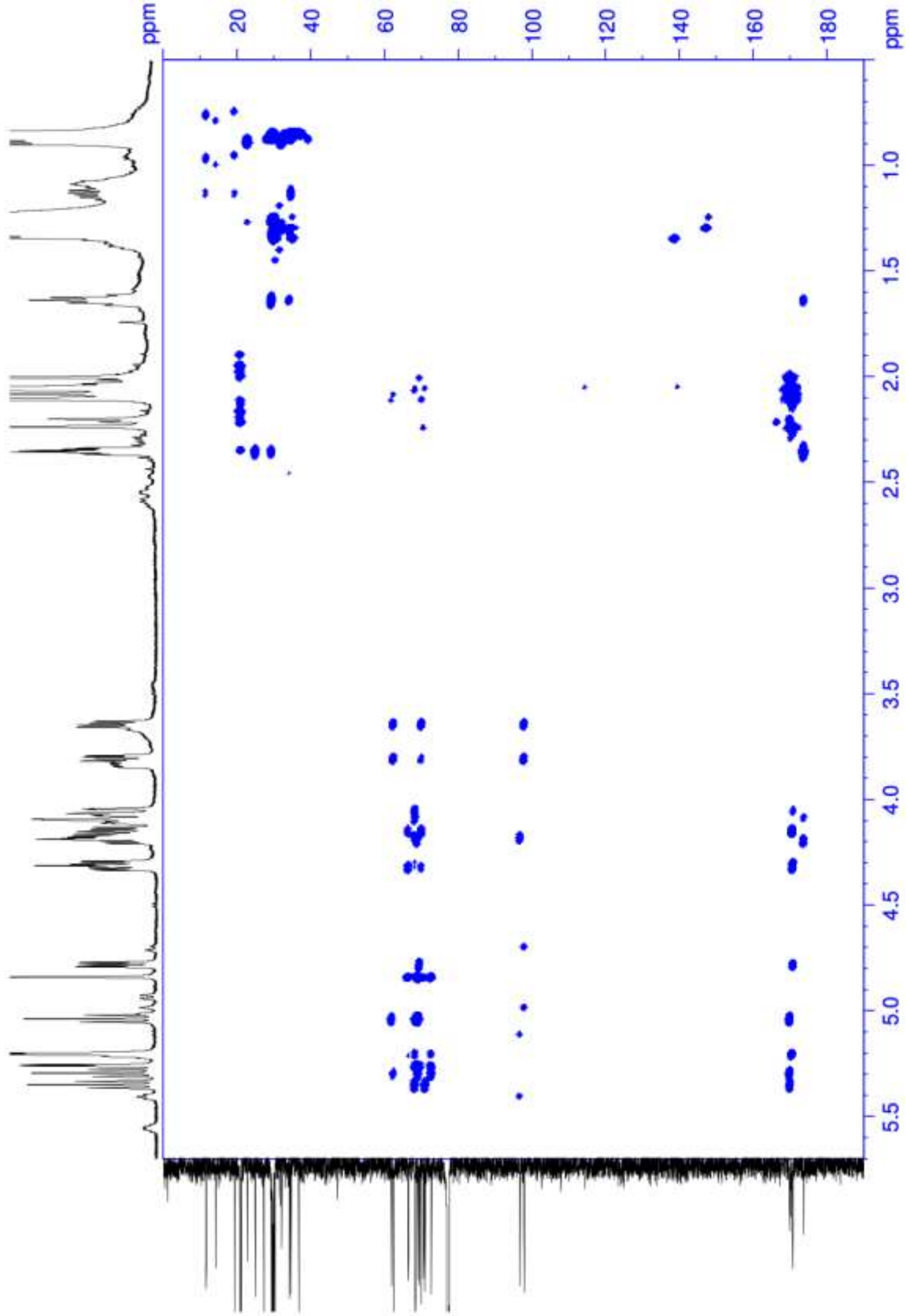

Figure S50 HRESIMS spectrum of **7**

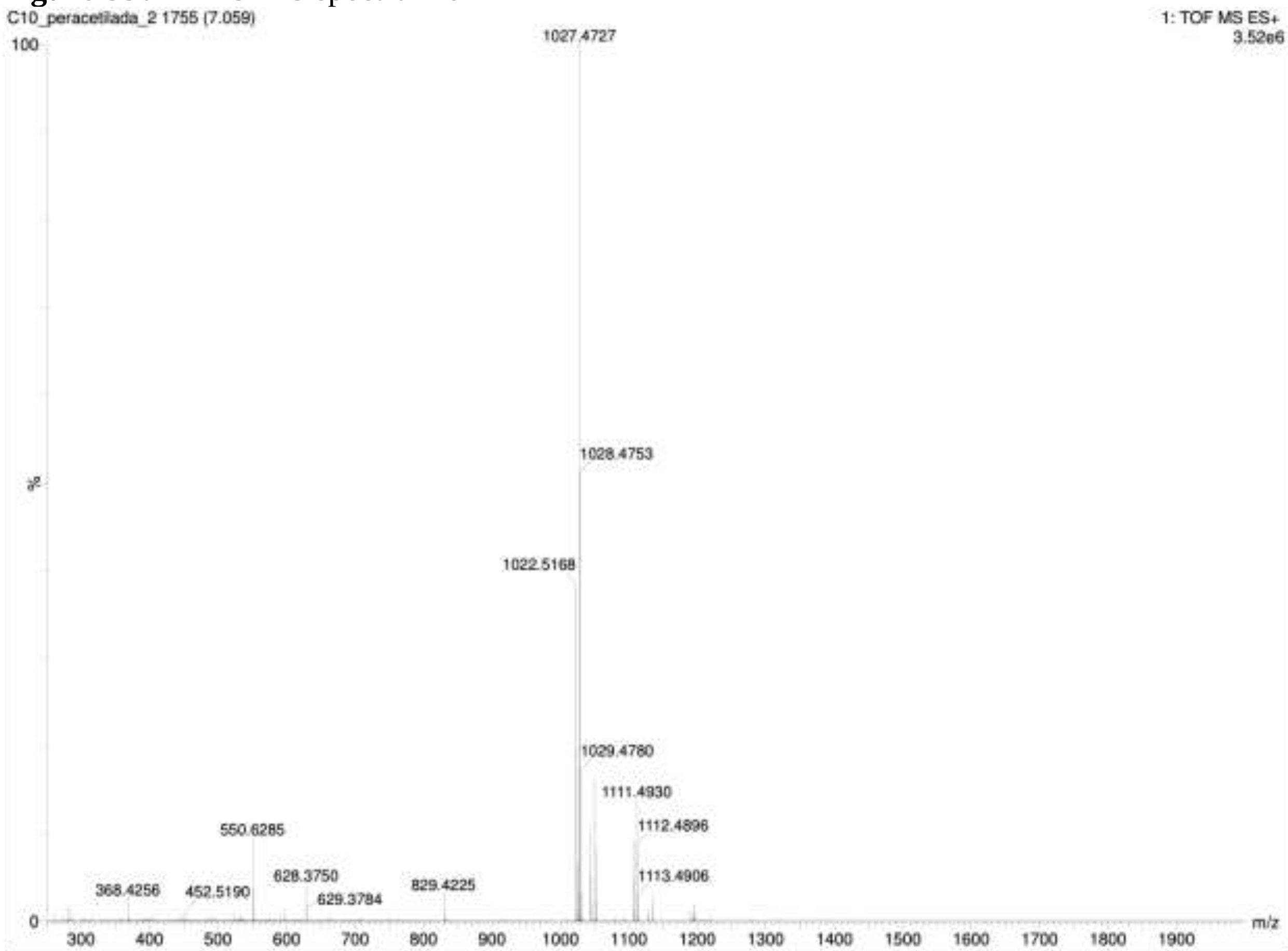

Figure S51 ^{1}H NMR spectrum of testacoside D peracetate (**8**) ($CDCl_3$; 600 MHz)

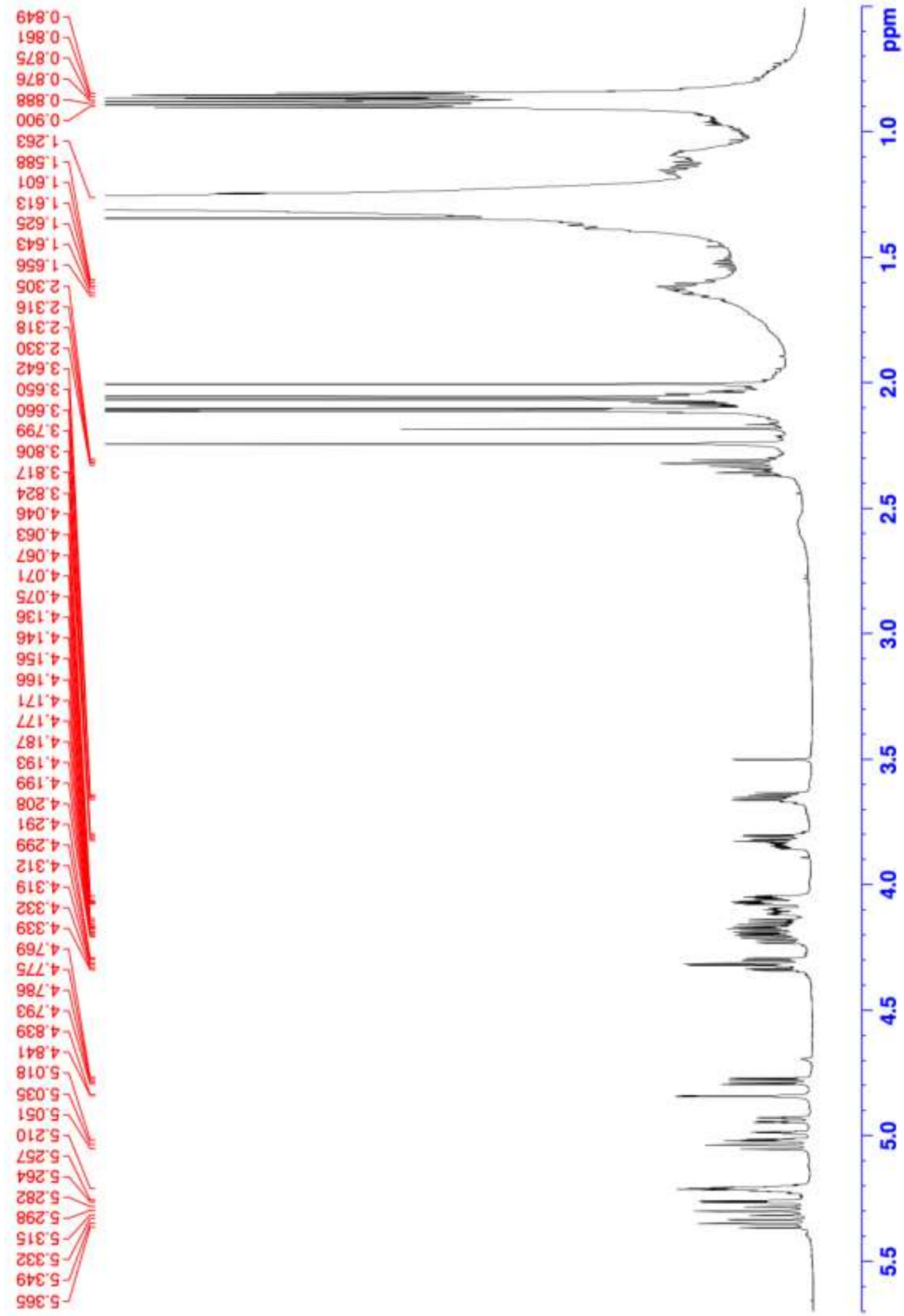

Figure S52 ^{13}C NMR spectrum of **8** ($CDCl_3$; 150 MHz)

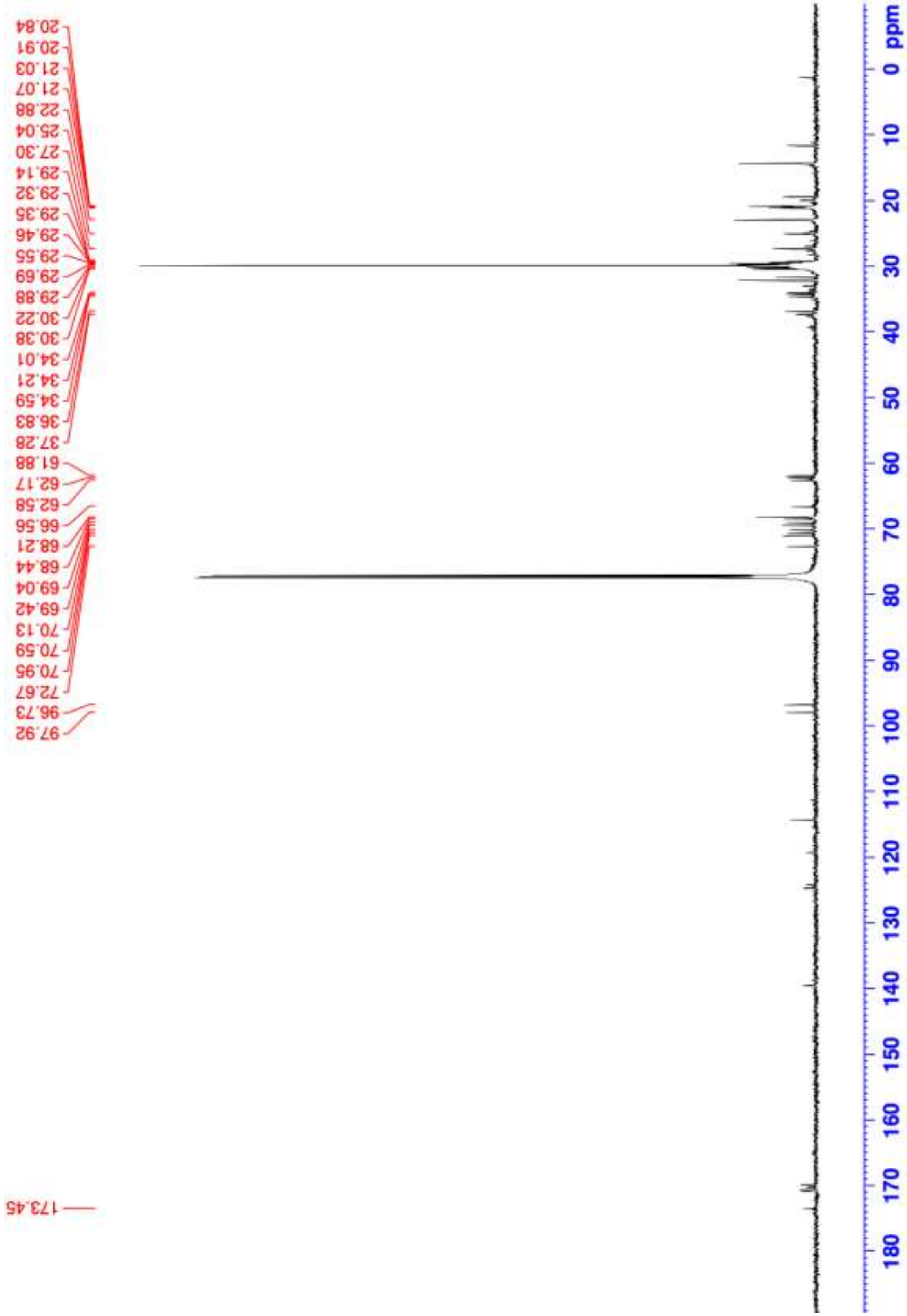

Figure S53 DEPT-135 spectrum of **8** ($CDCl_3$; 150 MHz)

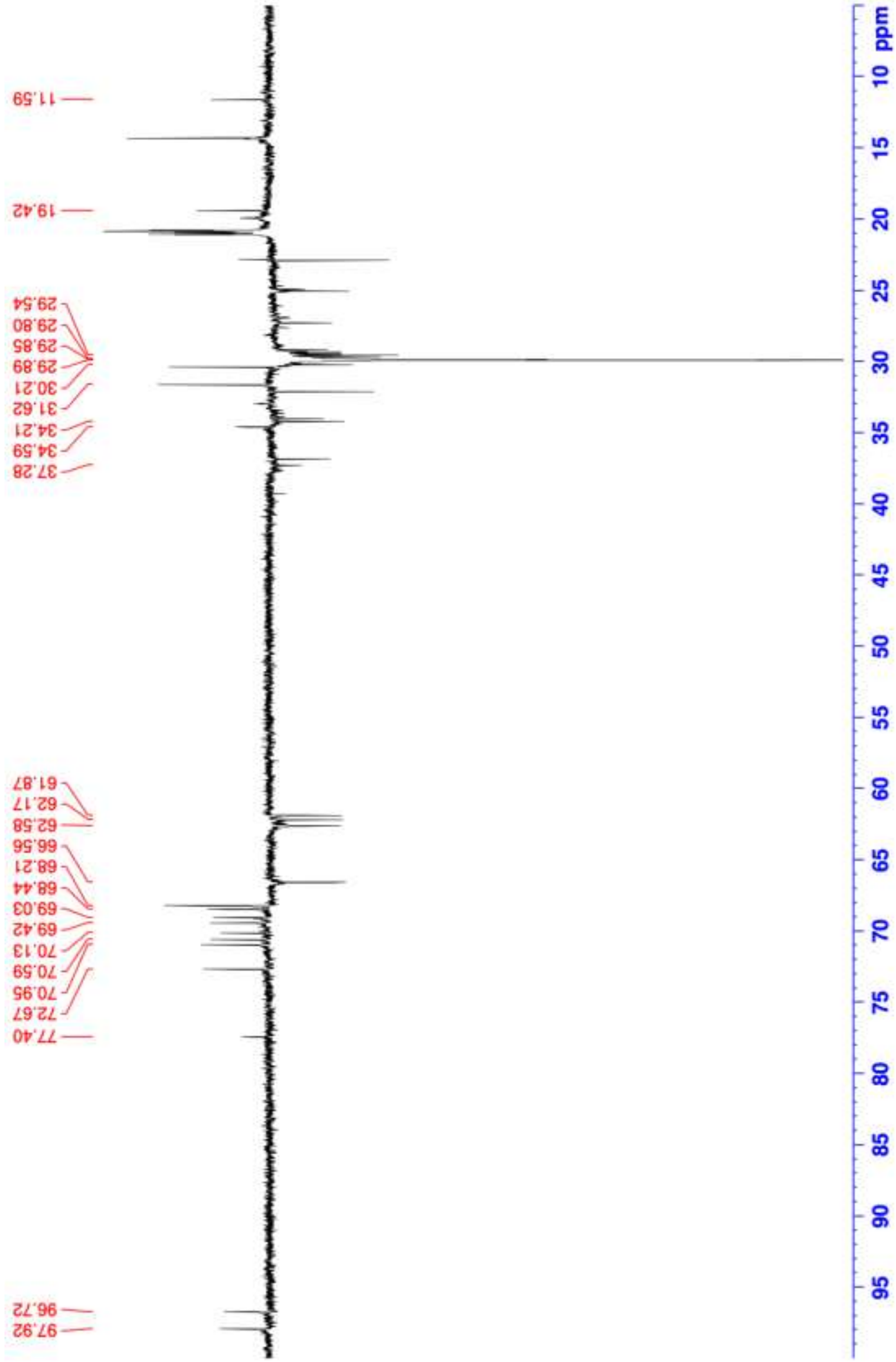

Figure S54 COSY spectrum of **8** ($CDCl_3$; 600 MHz)

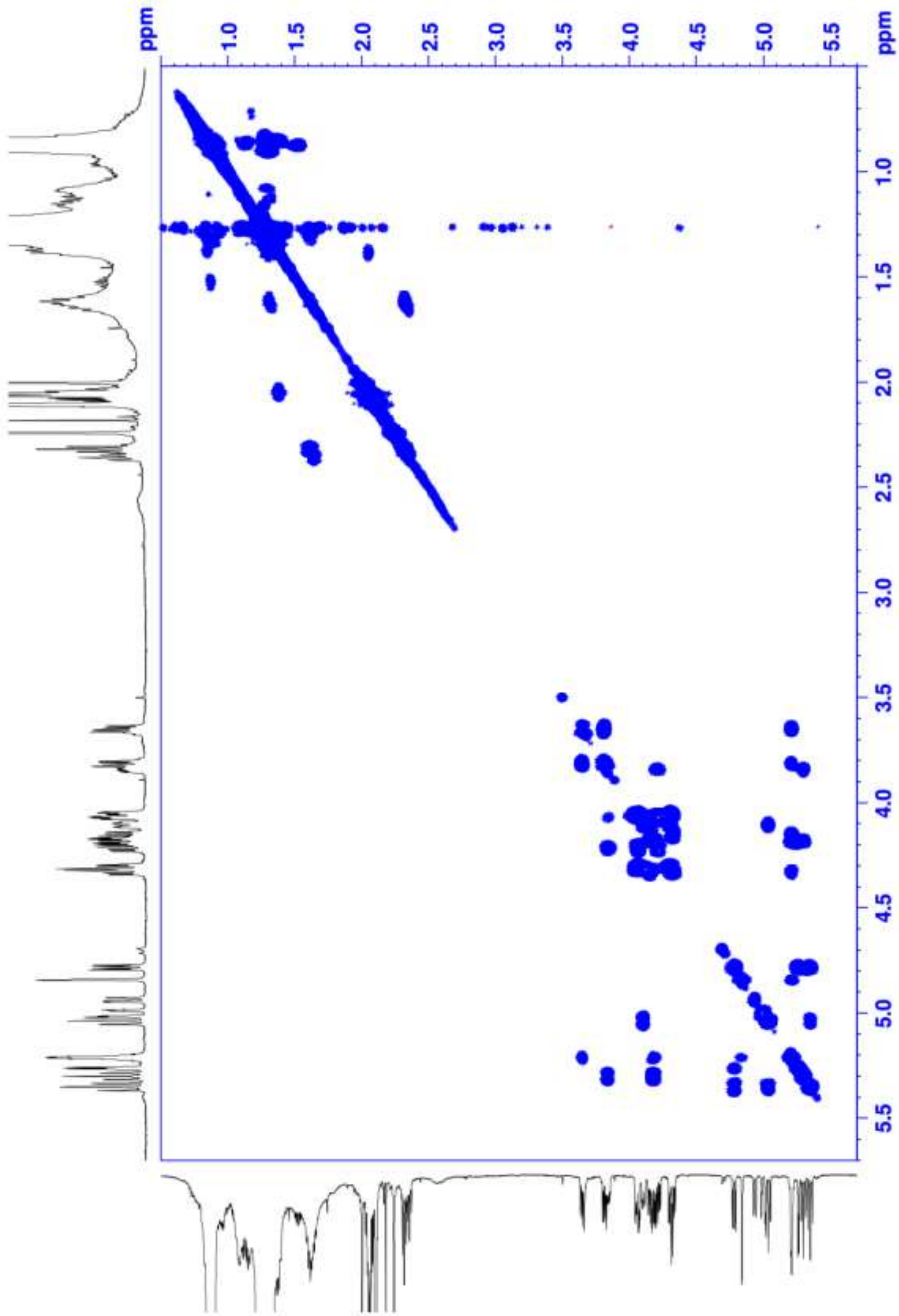

Figure S55 HSQC spectrum of **8** ($CDCl_3$; 600 MHz)

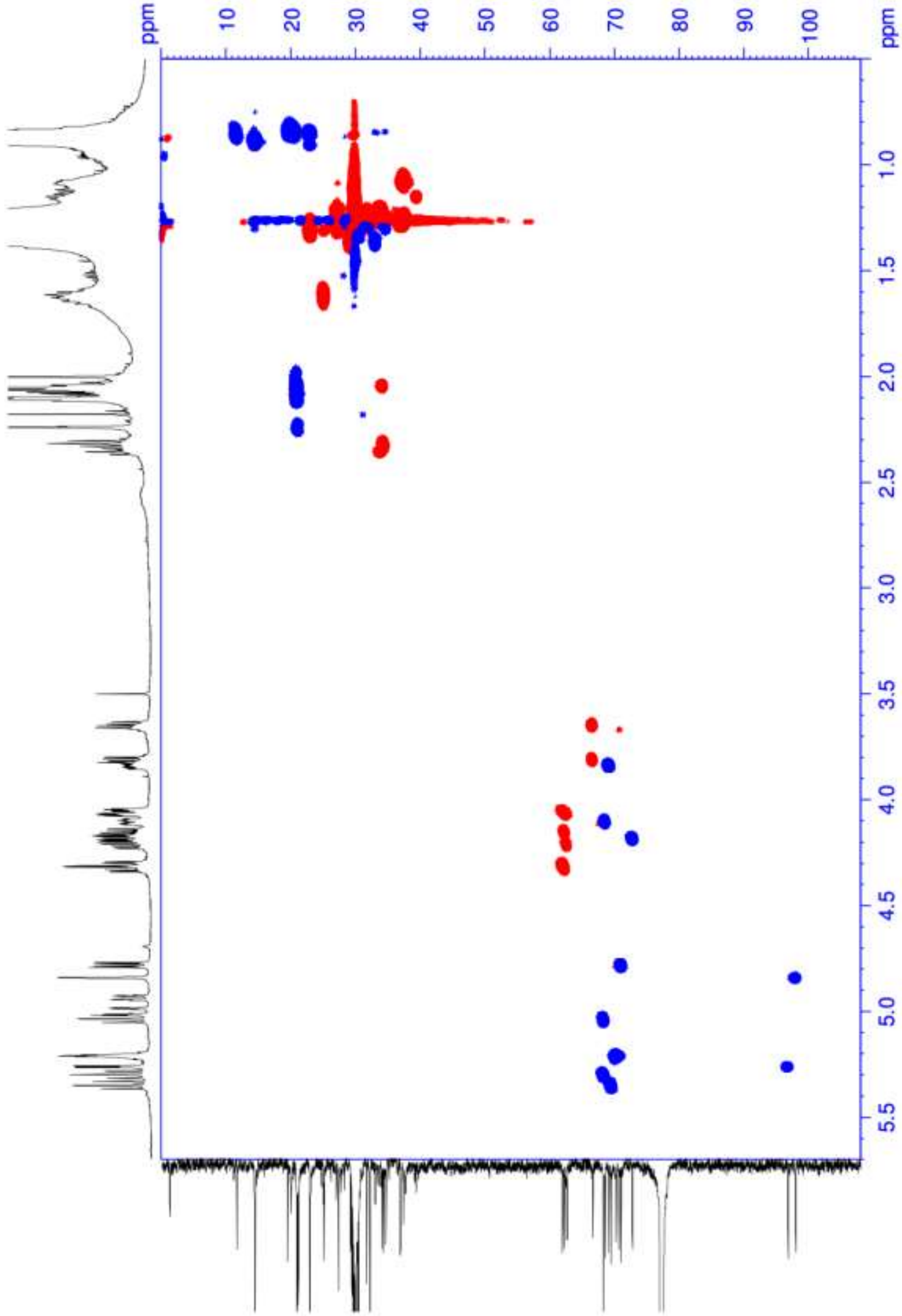

Figure S56 HMBC spectrum of **8** ($CDCl_3$; 600 MHz)

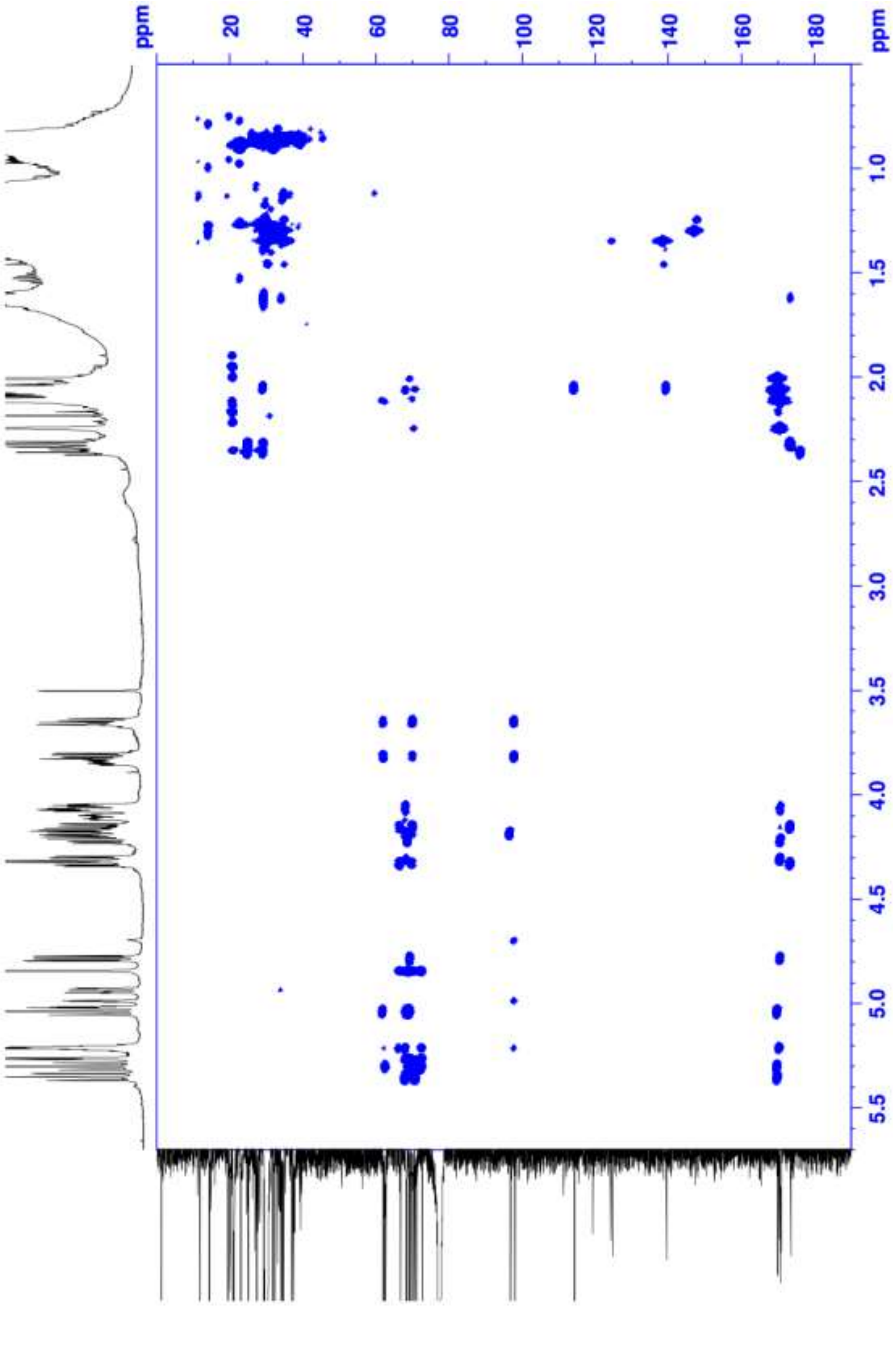

Figure S57. Neighbour-joining tree based on partial 16S rRNA gene sequences (1063 nt) showing relationships between isolate **J55** *Microbacterium testaceum* and closely related *Microbacterium* species. Asterisks indicate branches of the tree that were also found using the maximum-parsimony and minimum-evolution tree-making algorithms. Numbers at the nodes are percentage bootstrap values based on 1,000 resampled datasets. Bar 0.002 substitutions per nucleotide position.

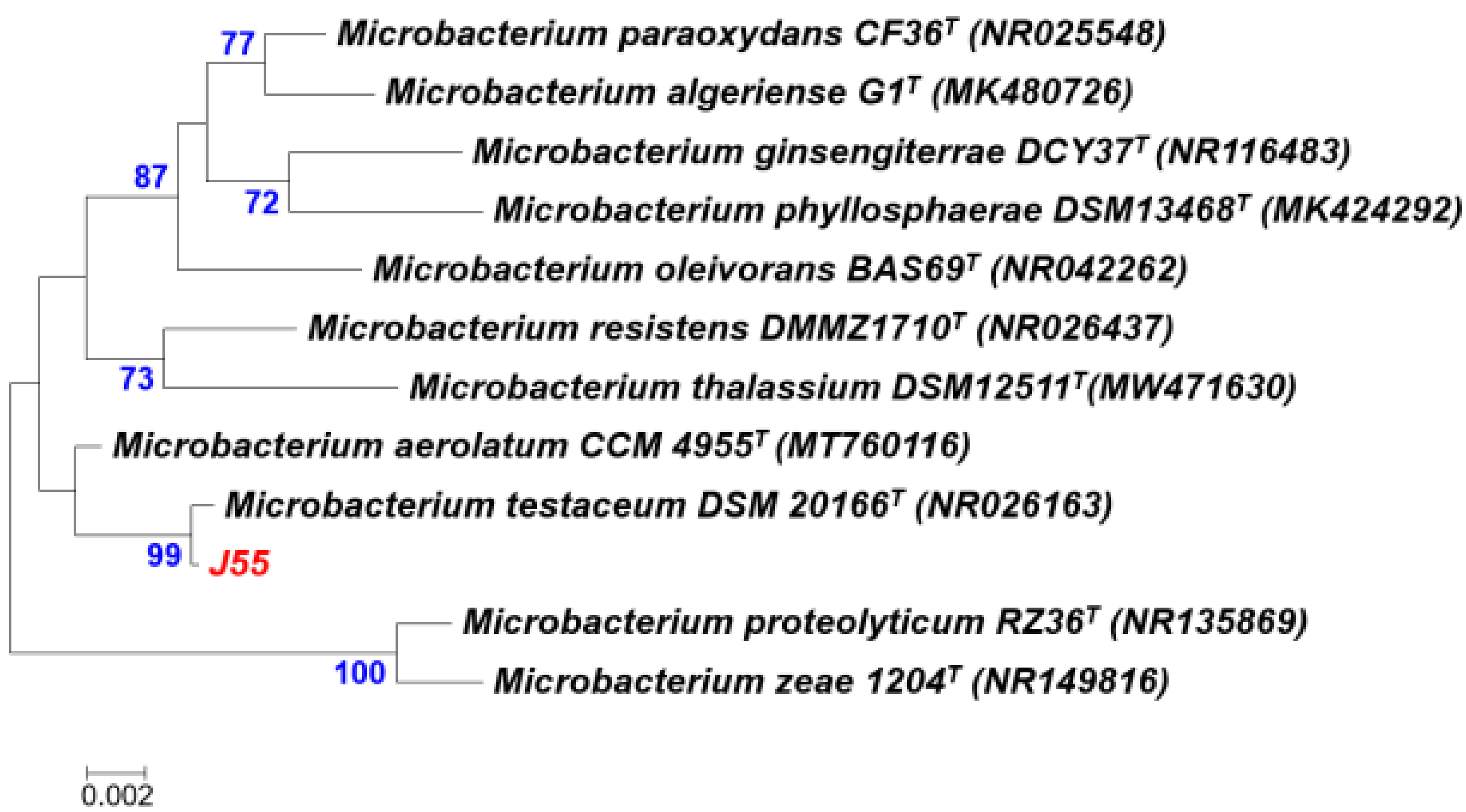

Comparison of partial 16S rRNA gene sequence of the isolate with corresponding sequences of phylogenetically related species with validly published names showed that it formed a branch in the *Microbacterium* 16S rRNA gene tree with the type strain of *M. testaceum.* A relationship supported by all of the tree- making algorithms and a bootstrap value of 70 % can be visualized. The 16S rRNA gene similarity between **J55** and **DSM** 20166^{T}organisms was 99.91%. Isolate **J55** showed 98.40% sequence similarity with the type strain of *M. aerolatum.*
